# Determining the effects of trastuzumab, cetuximab and afatinib by phosphoprotein, gene expression and phenotypic analysis in gastric cancer cell lines

**DOI:** 10.1186/s12885-020-07540-7

**Published:** 2020-10-28

**Authors:** Karolin Ebert, Gwen Zwingenberger, Elena Barbaria, Simone Keller, Corinna Heck, Rouven Arnold, Vanessa Hollerieth, Julian Mattes, Robert Geffers, Elba Raimúndez, Jan Hasenauer, Birgit Luber

**Affiliations:** 1 Fakultät für Medizin, Technische Universität München, Klinikum rechts der Isar, Institut für Allgemeine Pathologie und Pathologische Anatomie, 81675 München, Germany; 2 MATTES Medical Imaging GmbH, A-4232, Hagenberg, Austria; 3 Helmholtz Zentrum für Infektionsforschung, 38124 Braunschweig, Germany; 4 Helmholtz Zentrum München-German Research Center for Environmental Health, Institute of Computational Biology, 85764 Neuherberg, Germany; 5 Center for Mathematics, Technische Universität München, 85748 Garching, Germany; 6 Faculty of Mathematics and Natural Sciences, University of Bonn, 53113 Bonn, Germany

**Keywords:** Trastuzumab, Cetuximab, Afatinib, Gastric cancer, Motility, Phosphoprotein, Gene expression

## Abstract

**Background:**

Gastric cancer is the fifth most frequently diagnosed cancer and the third leading cause of cancer death worldwide. The molecular mechanisms of action for anti-HER-family drugs in gastric cancer cells are incompletely understood. We compared the molecular effects of trastuzumab and the other HER-family targeting drugs cetuximab and afatinib on phosphoprotein and gene expression level to gain insights into the regulated pathways. Moreover, we intended to identify genes involved in phenotypic effects of anti-HER therapies.

**Methods:**

A time-resolved analysis of downstream intracellular kinases following EGF, cetuximab, trastuzumab and afatinib treatment was performed by Luminex analysis in the gastric cancer cell lines Hs746T, MKN1, MKN7 and NCI-N87. The changes in gene expression after treatment of the gastric cancer cell lines with EGF, cetuximab, trastuzumab or afatinib for 4 or 24 h were analyzed by RNA sequencing. Significantly enriched pathways and gene ontology terms were identified by functional enrichment analysis. Furthermore, effects of trastuzumab and afatinib on cell motility and apoptosis were analyzed by time-lapse microscopy and western blot for cleaved caspase 3.

**Results:**

The Luminex analysis of kinase activity revealed no effects of trastuzumab, while alterations of AKT1, MAPK3, MEK1 and p70S6K1 activations were observed under cetuximab and afatinib treatment. On gene expression level, cetuximab mainly affected the signaling pathways, whereas afatinib had an effect on both signaling and cell cycle pathways. In contrast, trastuzumab had little effects on gene expression. Afatinib reduced average speed in MKN1 and MKN7 cells and induced apoptosis in NCI-N87 cells. Following treatment with afatinib, a list of 14 genes that might be involved in the decrease of cell motility and a list of 44 genes that might have a potential role in induction of apoptosis was suggested. The importance of one of these genes (*HBEGF*) as regulator of motility was confirmed by knockdown experiments.

**Conclusions:**

Taken together, we described the different molecular effects of trastuzumab, cetuximab and afatinib on kinase activity and gene expression. The phenotypic changes following afatinib treatment were reflected by altered biological functions indicated by overrepresentation of gene ontology terms. The importance of identified genes for cell motility was validated in case of *HBEGF*.

**Supplementary information:**

**Supplementary information** accompanies this paper at 10.1186/s12885-020-07540-7.

## Background

Gastric cancer is still a major health concern with worldwide 1,033,701 new cases and 782,685 deaths in 2018 [1]. Patients with HER2-positive recurrent or metastatic gastric cancer benefit from treatment with trastuzumab in combination with platin-fluoropyrimidine chemotherapy [2]. Since not all patients respond or develop resistance during treatment with the HER2-antibody trastuzumab, alternative treatment options are needed [3]. The survival benefit following therapy with the EGFR-antibody cetuximab in combination with chemotherapy in advanced esophago-gastric cancer was investigated in the EXPAND study. The addition of cetuximab did not improve progression-free survival in these patients [4]. However, the identification of biomarkers could help to identify patients which may benefit from cetuximab treatment. Currently ongoing clinical trials are investigating first or second line therapies with the pan-HER tyrosine kinase inhibitor afatinib [5–7].

The molecular mechanisms of action and resistance mechanisms of these targeted therapies are only partly understood. Several mutations in HER2 were reported, which might be associated with trastuzumab resistance [8–10]. Various RTKs, such as AXL [11], EGFR [12], HER3 [13, 14] and MET [15–17] can compensate the inhibition of signaling pathways by anti-HER therapies. Moreover, the downstream signaling pathway can be maintained by genetic alterations, such as mutations of the PI3K catalytic subunit (PIK3CA) or low PTEN expression [18–20]. We previously investigated the effects of trastuzumab and afatinib on the signaling network in gastric cancer cell lines with one time point of treatment. Even though HER2 activation was significantly reduced in NCI-N87 cells, no effect on the downstream kinase activity was observed under trastuzumab treatment [21]. Therefore, we now analyzed the time-resolved effects of the different anti-HER treatments on the downstream signaling pathways that were not addressed by earlier investigations.

Besides the effects on cellular signaling [21] and gene expression [22–24], anti-HER therapies are known to influence the phenotypic behavior of cancer cells. For example, afatinib is known to reduce cell migration and induce apoptosis in various cancer entities [25–34]. It is currently unknown which genes contribute to the altered phenotypic behavior after anti-HER-family therapies. The identification of molecular processes underlying these phenotypic responses could help to deepen the understanding of the mechanism of action of these targeted therapies and may help selecting the appropriate treatment.

During this work, we aimed to compare the molecular and phenotypic effects of the anti-HER-family drugs trastuzumab, cetuximab and afatinib. Additionally, we intended to understand the molecular mechanisms of response to different drugs and we aimed to identify genes involved in phenotypic effects of anti-HER therapies.

## Methods

### Cell culture

The gastric cancer cell lines MKN1 (Cell Bank RIKEN BioResource Center), MKN7 (Cell Bank RIKEN BioResource Center), NCI-N87 (ATCC Cell Biology Collection) and Hs746T (ATCC Cell Biology Collection) were cultured as described earlier [21, 35]. All four cell lines were established from metastatic sites of gastric carcinomas (Additional file 1).

### Luminex analyses

Luminex as high throughput method was used to analyze the activation of multiple phosphoproteins. Cells were treated with EGF (5 ng/ml, Sigma Aldrich), cetuximab (Cet, 1 μg/ml, Merck), trastuzumab (Tra, 5 μg/ml, Roche) or afatinib (Afa, 0.5 μM, Biozol) for 3, 5, 15, 30, 60 and 240 min. We used untreated instead of DMSO-treated cells as control for afatinib since we previously showed minor effects of DMSO compared to afatinib treatment in western blot experiments [21]. Trastuzumab and cetuximab are solved according to the solutions used in patients (trastuzumab solvent (3.36 mg L-Histidine HCl, 2.16 mg L-Histidine, 136.2 mg trehalose, dihydrate, and 0.6 mg polysorbate 20 solved in 7.2 ml sterile water) [21], cetuximab solvent (8.48 mg/ml NaCl, 1.88 mg/ml Na_2_HPO_4_x7H_2_O, 0.41 mg/ml NaH_2_PO_4_xH_2_O)). Thirteen different phosphoproteins (EGFR (Y1068), FAK1 (Y397), SRC (Y419), AKT1 (S473), GSK3A (S21), MAPK3 (T202/Y204), MEK1 (S217/S221), NFκB (S536), IκBA (S32/S36), p70S6K1 (T389), MAPK12 (T180/Y182), STAT3 (Y705), STAT5 (Y694)) were chosen. Luminex data were log2-transformed and analyzed using the MATLAB statistical toolbox. Batch effects were corrected by fitting a linear mixed effect model for each protein and subtracting the inferred batch-specific contribution. The batch-corrected data were used to compute the median fold changes between pairs of experimental conditions. We considered only changes with an absolute median fold change > 1.5. The statistical significance of changes was assessed using the Student’s t-test. In the cluster analyses the *p*-values were adjusted using Bonferroni-Holm method. For visualization purposes, the adjusted *p* values were grouped (≤0.001; 0.001–0.01; 0.01–0.05). For direct comparison of Luminex data to western blot results, the antilogarithm of batch-corrected Luminex dataset was taken and the untreated samples was set to 100%, exactly as it was done for the samples analyzed by western blot. Pearson correlation coefficients with respective significance were calculated comparing the protein activation between Luminex and western blot.

### RNA extraction

Cells were seeded in 10 cm dishes 1 day before treatment. MKN1, MKN7 and Hs746T cells were plated at a density of 1.7 × 10^4^ cells/cm^2^ and NCI-N87 at 2 × 10^4^ cells/cm^2^. Medium was changed 2 h before treatment. Cells were treated with EGF (5 ng/ml, Sigma Aldrich), cetuximab (Cet, 1 μg/ml, Merck), trastuzumab (Tra, 5 μg/ml, Roche), afatinib (Afa, 0.5 μM, Biozol) or dimethylsulfoxid (DMSO, 0.05%, afatinib solvent control) for 4 h or 24 h. RNA and micro RNA were isolated using the mirVana™ miRNA Isolation Kit (Thermo Fisher Scientific), according to manufacturer’s instructions. The RNA was eluted in nuclease-free water. DNase digestion was performed using the DNA-free™ DNA Removal Kit (Thermo Fisher Scientific) according to manufacturer’s instructions.

### Next generation sequencing

Quality and integrity of total RNA was controlled on Agilent Technologies 2100 Bioanalyzer (Agilent Technologies). The RNA sequencing library was generated from 500 ng total RNA using Dynabeads® mRNA DIRECT™ Micro Purification Kit (Thermo Fisher Scientific) for mRNA purification followed by NEBNext® Ultra™ II Directional RNA Library Prep Kit (New England BioLabs) according to manufacturer’s protocols. The libraries were sequenced on Illumina NovaSeq 6000 using NovaSeq 6000 S2 Reagent Kit (200 cycles, paired end run) with an average of 3 × 10^7^ reads per RNA sample. Primary data analysis was performed as indicated in Additional file 1.

### Functional enrichment analysis

Functional analysis was performed by R package “clusterProfiler 3.5.6” [36]. The GeneRatio is defined as the number of differentially expressed genes within the geneset divided by the total number of differentially expressed genes. As an example, a GeneRatio of 6/43 means that 6 out of 43 differentially expressed genes belong to this pathway. The BgRatio is defined as the number of genes within this geneset divided by the number of genes within the collection of genesets. As an example, a BgRatio of 70/5844 means that 70 out of 5844 genes belong to this pathway.

### Time-lapse microscopy

Plastic culture dishes (TPP, growth area 9.2 cm^2^) were coated with laminin (1.65 μg/cm^2^, Sigma-Aldrich) while glass-bottom culture dishes (MatTek Corporation, growth area 9.2 cm^2^) were coated with collagen I (16.3 μg/cm^2^, VWR). MKN7 cells were plated onto laminin-coated dishes at a density of 1.1 × 10^5^ cells/dish. MKN1, Hs746T and NCI-N87 cells were plated onto collagen-coated dishes at densities of 2 × 10^5^ cells/dish, 1.5 × 10^5^ cells/dish and 2.5 × 10^5^ cells/dish, respectively. MKN7 cells were plated 24 h before filming was started. MKN1, Hs746T and NCI-N87 cells were plated 1 h before filming was started. Time-lapse microscopy and determination of *motile cells* and *average speed* were carried out as described earlier [35, 37]: Cells were cultivated in a microscope-coupled incubation chamber (5% CO_2_, 37 °C). Phase-contrast images were taken every 3 min for 7 h with an Axio Observer A1 microscope (Zeiss) with a 10×/0.3 Ph1 objective lens. The *percentage of motile cells* was determined by counting the cells in a microscopic field that moved completely out of the initial area within the observation time of 7 h. Only attached non-dividing cells that did not leave the observation field during the video were analysed. The *average cell speed* was calculated by dividing the manually determined pathway lengths of the cells by the observation time of 7 h.

### Computational cellular motility analysis

Only cells present in all frames t of a video of moving cells under time-lapse microscopy have been considered. For each such cell the position (x, y) in the center of its nucleus in a frame t has been determined and recorded as (x, y, t). This has been done for any frame t. This leads to a sequence of vectors (x, y, t), the trajectory of the cell, where t is varying from 1 to N, with N the total number of frames in the video. Thereby, the first point (x_1_, y_1_) of the trajectory, represented in the sequence by (x_1_, y_1_, 1), can either be calculated by an algorithm [38] or placed manually. In this paper manual placing was used. The positions in each successive frame have been computed by the algorithm of Debeir [39] with manual correction where necessary. In difficult cases the points are placed manually also in successive frames. A cell is considered to be present in a frame if its nucleus is completely visible. Only cells not undergoing cell division are considered. For a trajectory (x, y, t), 1 ≤ t ≤ N, its *approximate average speed* is calculated as described earlier [17]: the approximate average speed is defined as the approximate trajectory length divided by the total elapsed time of the video sequence.

### Western blot

Western blot analyses were performed as described earlier [40, 41]. MKN1, MKN7, Hs746T and NCI-N87 cells were treated for 24 h with EGF (5 ng/ml, Sigma Aldrich), cetuximab (1 μg/ml, Merck), trastuzumab (5 μg/ml, Roche) or afatinib (0.5 μM, Biozol). Antibodies against pAKT (1:2000 in 5% BSA TBST,# 9271, Cell Signaling Technology), pMEK1/2 (1:1000 in 5% BSA TBST, # 9154, Cell Signaling Technology), ppMAPK3 (1:2000 in 5% milk TBS-T,# 9101, Cell Signaling Technology), pp70S6K (1:1000 in 5% BSA TBST,# 9205, Cell Signaling Technology), cleaved caspase 3 (Asp 175) (1:1000 in 5% skimmed milk TBST, #9664, Cell Signaling Technology), α-tubulin (1:10000 in 5% skimmed milk TBST, #T9026, Sigma-Aldrich), anti-rabbit IgG (1:2000 in TBST, #7047, Cell Signaling Technologies) and anti-mouse IgG (1:10000 in 5% skimmed milk TBST, #NA931, GE healthcare, distributed by VWR) were used.

### Transfection with siRNA and RNA extraction

Medium was exchanged to antibiotic-free medium 1 day after plating of MKN1 cells at a density of 1.7 × 10^4^ cells/cm^2^. Cells were transfected using Lipofectamine 2000 (Thermo Fisher Scientific) and HBEGF siRNA (Flexi Tube Gene Solution (pool of 4 different siRNAs), Qiagen) 2 h after medium replacement. The unlabeled and labeled (AF 488) All Star Negative Control siRNA (Qiagen) were used as controls. RNA was extracted on day 1 after transfection (RNeasy Mini Kit, Qiagen) to check the knockdown efficiency by qPCR. The efficiency was assessed with AF 488-labeled negative control siRNA 1 day after transfection. More than 90% of MKN1 cells resulted successfully transfected.

### Quantitative PCR

RNA was transcribed into cDNA using the High-Capacity cDNA Reverse Transcription Kit (Thermo Fisher Scientific). Candidate gene expression was measured using the TaqMan Gene Expression Assays for HBEGF (Hs00181813_m1), CD274 (Hs01125296_m1) or ACTB (Hs01060665_g1, reference) and the TaqMan Universal PCR Master Mix (Thermo Fisher Scientific). All procedures were carried out according to manufacturer’s instructions. The LightCycler® 480 instrument and software (Roche) were used to determine the relative gene expression.

### Statistical analyses for in vitro experiments

Data are presented as mean of at least three independent experiments with standard deviation. One-sample or two-sample t-test was calculated using SPSS Statistics (IBM). Statistically significant differences are represented by *p < 0.05, **p < 0.01 or ***p < 0.001. Gene expression data are presented with log2-transformed fold-change (log2FC) and *p*-value adjusted according to Benjamini-Hochberg (FDR, p.adjust).

### Database search for genetic alterations

A database search was performed for genetic alterations in genes encoding the 13 different investigated phosphoproteins (EGFR (Y1068): *EGFR* gene; FAK1 (Y397): *PTK2* gene; SRC (Y419): *SRC* gene; AKT1 (S473): *AKT1* gene; GSK3A (S21): *GSK3A* gene; MAPK3 (T202/Y204): *MAPK3* gene, MEK1 (S217/S221): *MAP 2 K1* gene; NFκB (S536): *NFkB* gene; IκBA (S32/S36): *IkBA* gene; p70S6K1 (T389): *RPS6KB1* gene; MAPK12 (T180/Y182): *MAPK12* gene; STAT3 (Y705): *STAT3* gene, STAT5 (Y694): *STAT5* gene) in cell lines MKN1, MKN7, NCI-N87 and Hs746T. Mutational data were derived from the last public COSMIC version (v90). The database search was performed on March 28, 2020.

## Results

### Workflow for phosphoprotein, gene expression and phenotypic analysis

The cell lines MKN1, MKN7, NCI-N87 and Hs746T were established from metastatic sites of gastric carcinomas (Additional file 1).

**Fig. 1:**
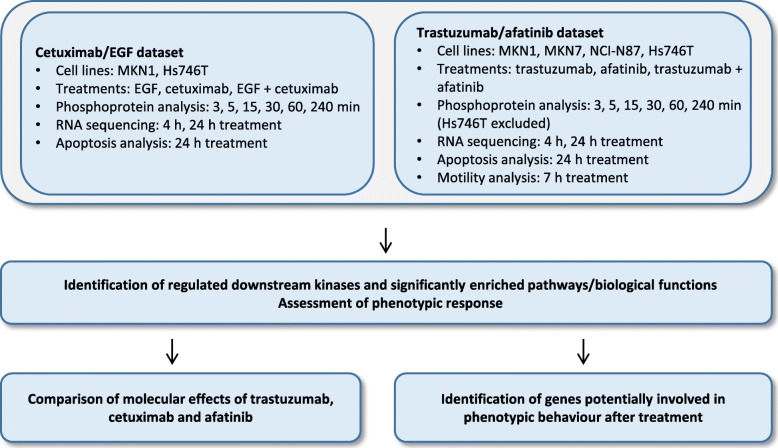
Workflow for phosphoprotein, gene expression and phenotypic analysis. MKN1 and Hs746T cells were treated with EGF, cetuximab or the combination of both. MKN1 cells are cetuximab responder and Hs746T cells are cetuximab non-responder [35, 41]. MKN1, MKN7, NCI-N87 and Hs746T cells were treated with trastuzumab, afatinib or the combination of both. NCI-N87 cells are trastuzumab responder and MKN7 and MKN1 are trastuzumab non-responder [21, 42]. NCI-N87, MKN1 and MKN7 cells are afatinib responder and Hs746T cells are afatinib non-responder [21]. Phosphoprotein analysis of selected downstream kinases, RNA sequencing, apoptosis and motility analysis were performed to compare the molecular effects of cetuximab, trastuzumab and afatinib and to identify genes involved in the phenotypic effects of the treatments

MKN1 and Hs746T cells were treated with EGF, cetuximab or the combination of both. MKN1, MKN7, NCI-N87 and Hs746T cells were treated with trastuzumab, afatinib or the combination of both. Cell lines were selected according to the previously published response characterization. MKN1 cells are responsive to cetuximab treatment whereas Hs746T cells are not [35, 41]. In case of trastuzumab, NCI-N87 cells were described as responder and MKN7 and MKN1 cells as non-responder. NCI-N87, MKN1 and MKN7 cells were characterized as afatinib responder while Hs746T cells were described as afatinib non-responder [21, 42]. The phosphoprotein analysis of selected downstream kinases was performed by Luminex analysis. RNA sequencing was used to determine differentially expressed genes. Significantly enriched pathways and biological function (gene ontology) were determined by functional enrichment analysis of gene expression data. Additionally, cell motility and apoptosis were assessed by time-lapse microscopy and western blot, respectively. The molecular effects of trastuzumab, cetuximab and afatinib were compared on the level of intracellular signaling, gene expression and phenotype. Moreover, genes potentially involved on phenotypic behavior after afatinib treatment were identified (Fig. 1).

For a comprehensive characterization of the cell lines, a database search was performed for genetic alterations in genes encoding the 13 different investigated phosphoproteins. We found mutations in the *PTK2* gene in MKN1 (c.1332 + 44A > G, c.1464 + 44A > G, c.216 + 44A > G) and NCI-N87 cells (c.1093 + 120 T > C, c.1225 + 120 T > C). In MKN7 cells, we found mutations in the *EGFR* gene (c.2769C > T, c.2634C > T, c.*759C > T) and in the *GSK3A* gene (c.798-21C > T, c.552-21C > T). Type of mutation was substitution-coding silent for *EGFR* gene (c.2769C > T and c.2634C > T) and unknown for all other mutations. None of these mutations changes the amino acid sequence of the phosphoproteins. Thus, these informations were not considered for interpretation.

### Analysis of intracellular signaling

#### Cetuximab

Based on the results of the Luminex analysis, a hierarchical cluster analysis was performed, comparing all different treatment conditions to each other. Increasing phosphorylation/activation of proteins is indicated in red. Blue indicates decreasing phosphorylation/activation of proteins.

**Fig. 2:**
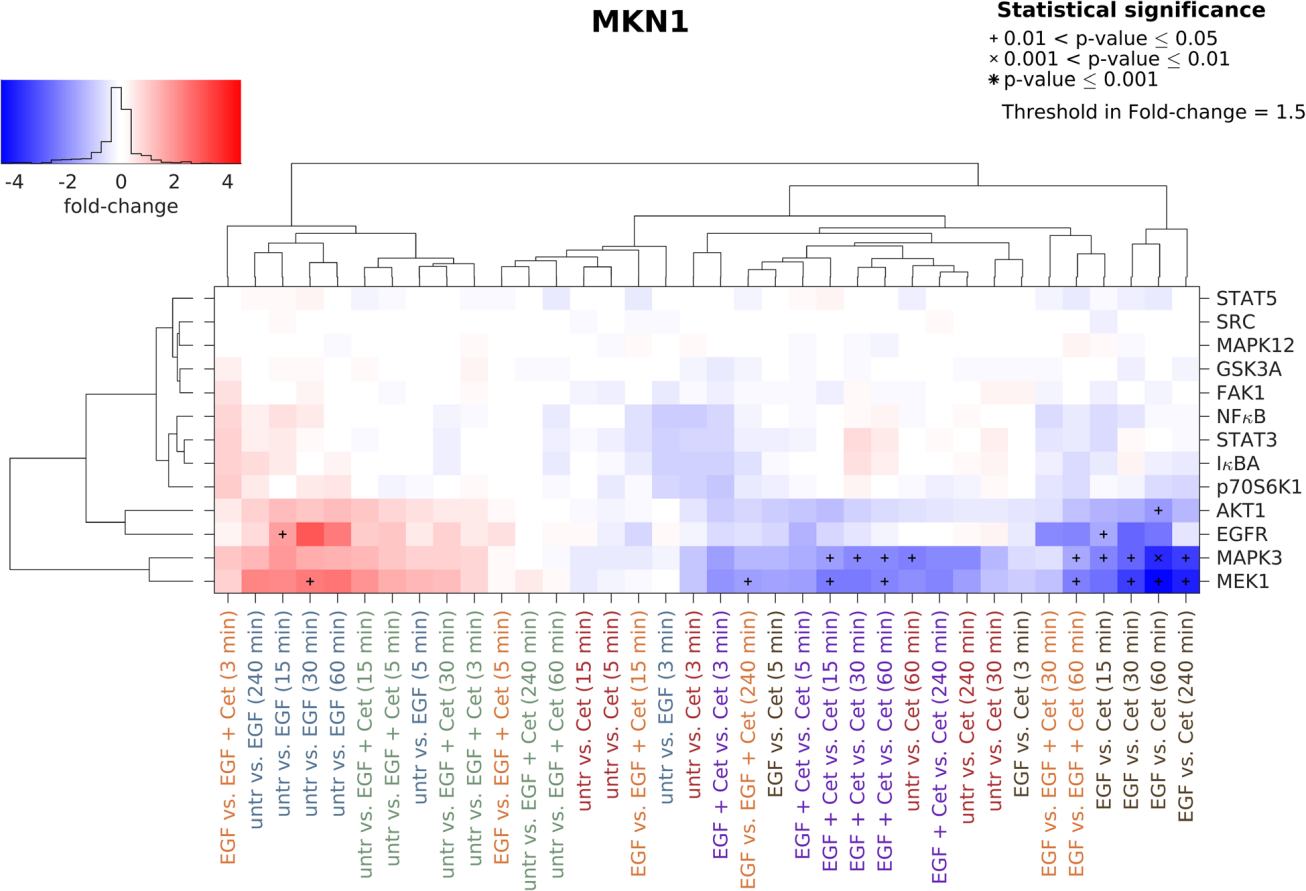
Effects of EGF and cetuximab on kinase phosphorylation in MKN1 cells. Luminex analysis was performed to detect the effects on protein tyrosine kinases in MKN1 cells induced by EGF and/or cetuximab. Cells were treated for 3, 5, 15, 30, 60 and 240 min with 5 ng/ml EGF, 1 μg/ml cetuximab or the combination of both. In the batch-corrected cluster analysis, the x-fold change of each activated protein is shown. Samples were clustered based on to the similarity of the activated proteins and treatment conditions. Significant effects between different treatment conditions are indicated by (*) with increasing size (0.01 < *p*-value ≤0.05, 0.001 < *p*-value ≤0.01 and *p*-value ≤0.001). Increasing protein phosphorylation/activation is indicated in red. Blue indicates decreasing protein phosphorylation/activation. Abbreviations: Cet = cetuximab, untr = untreated

Cetuximab and EGF treatment mainly regulated phosphorylation of MEK1, MAPK3, EGFR and AKT1 in MKN1 cells. MAPK3 and MEK1 cluster apart from all other proteins. By comparing untreated vs. EGF strongest increase was observed after 30 min treatment whereas the comparisons untreated vs. cetuximab and EGF vs. EGF + cetuximab revealed strongest reduction following 60 min treatment. Slight effects of cetuximab on EGFR activation were observed in presence of EGF only (Fig. 2). In contrast, no effects of the different treatments on kinase phosphorylation were observed in Hs746T cells (Fig. S1, Additional file 2).

#### Trastuzumab and afatinib

**Fig. 3:**
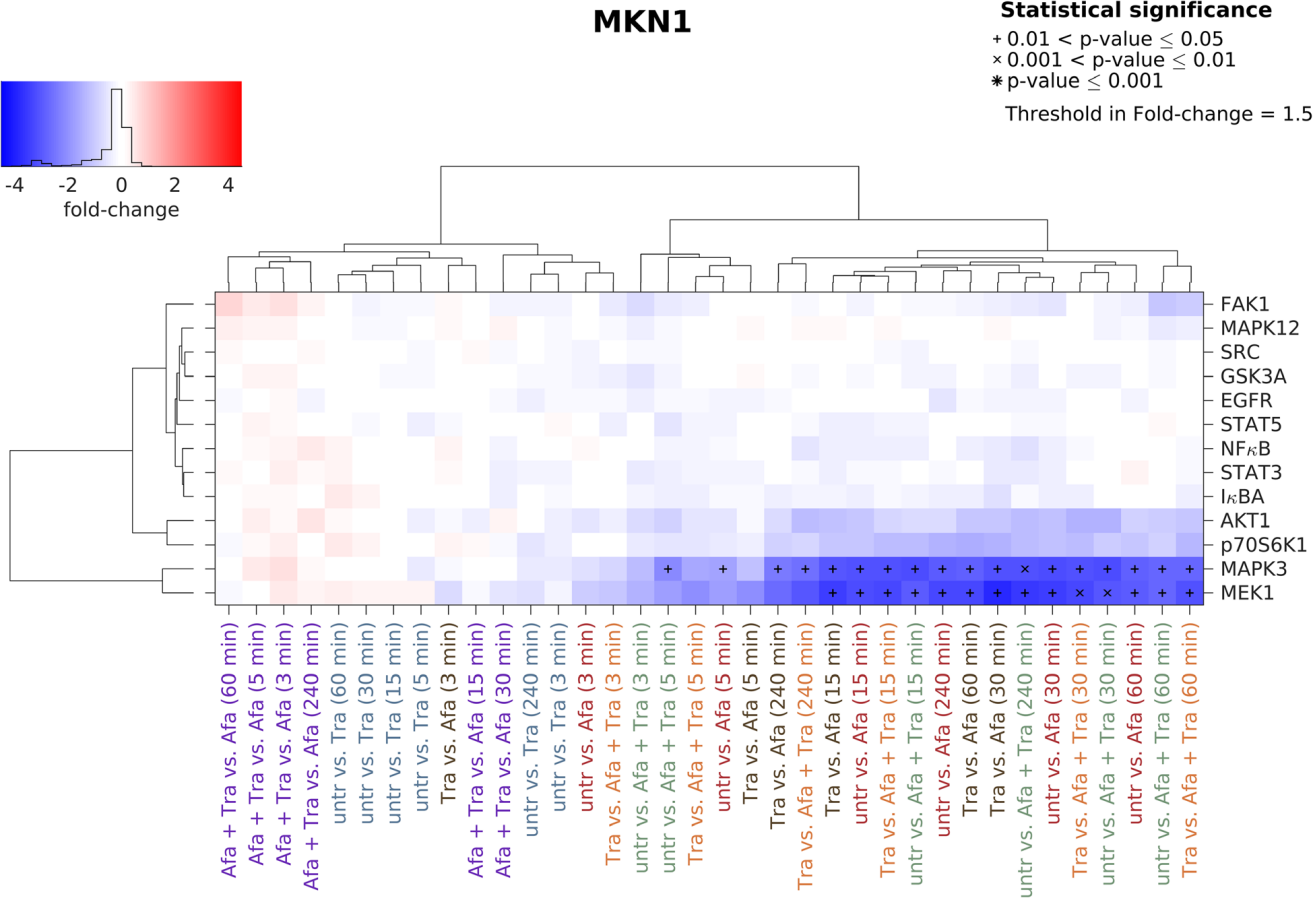
Effects of trastuzumab and afatinib on kinase phosphorylation in MKN1 cells. Luminex analysis was performed to detect the effects on protein tyrosine kinases in MKN1 cells induced by trastuzumab and/or afatinib. Cells were treated for 3, 5, 15, 30, 60 and 240 min with 0.5 μM afatinib, 5 μg/ml trastuzumab or the combination of both. In the batch-corrected cluster analysis, the x-fold change of each activated protein is shown. Samples were clustered based on to the similarity of the activated proteins and treatment conditions. Significant effects between different treatment conditions are indicated by (*) with increasing size (0.01 < *p*-value ≤0.05, 0.001 < *p*-value ≤0.01 and *p*-value ≤0.001). Increasing protein phosphorylation/activation is indicated in red. Blue indicates decreasing protein phosphorylation/activation. Abbreviations: Afa = afatinib, Tra = trastuzumab, untr = untreated

Trastuzumab treatment did not result in altered kinase phosphorylation regardless of treatment time in MKN1 cells. Afatinib treatment reduced the phosphorylation of MEK1, MAPK3, p70S6K1 and AKT1. Significant effects were observed for MAPK3 and MEK1, which cluster apart from all other proteins. By comparing untreated vs. afatinib strongest effects were observed following 15–240 min treatment. The combination of trastuzumab and afatinib resulted in a similar reduction in phosphorylated kinases when compared to afatinib alone (Fig. 3).

**Fig. 4:**
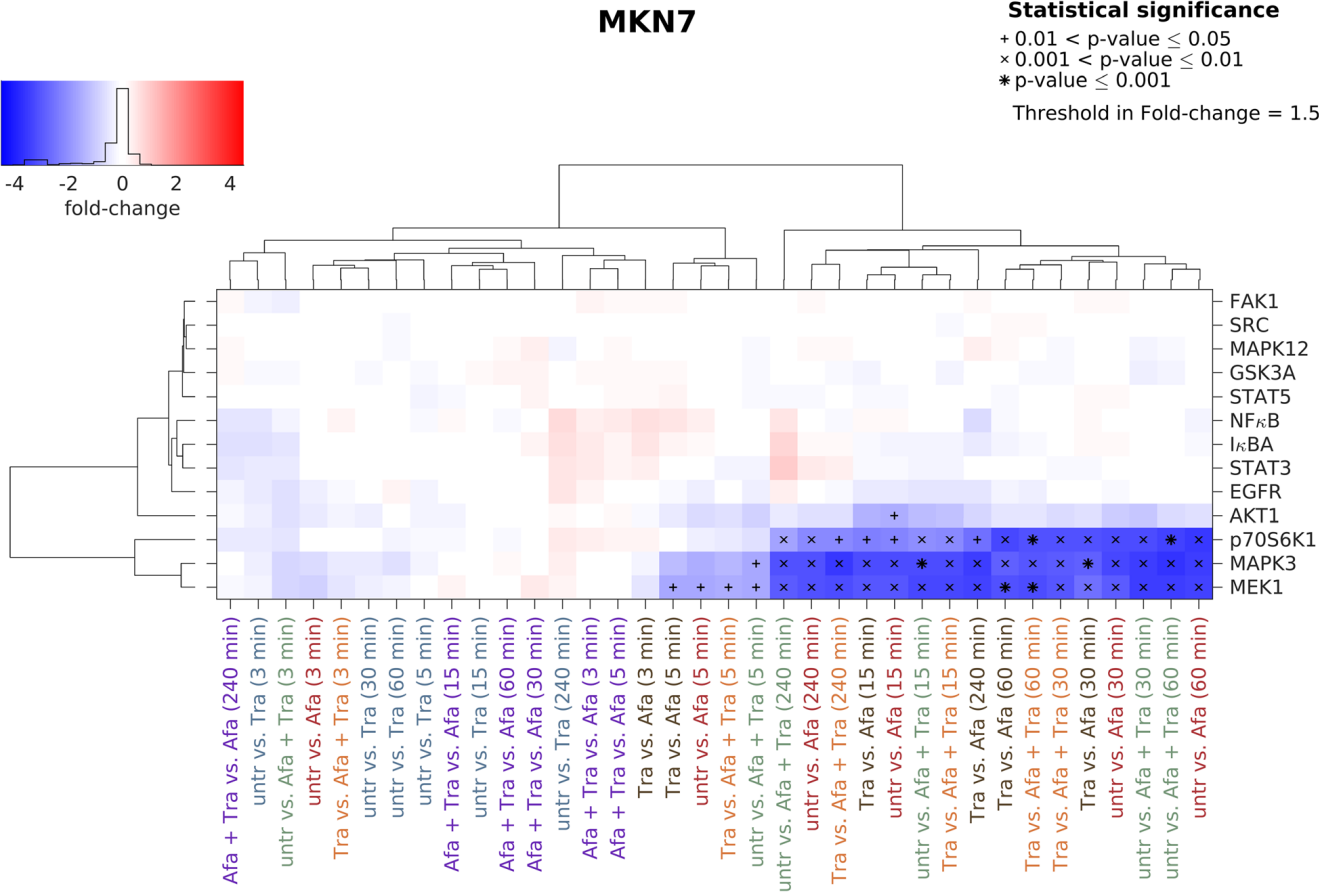
Effects of trastuzumab and afatinib on kinase phosphorylation in MKN7 cells. Luminex analysis was utilized to detect the effect of trastuzumab and/or afatinib on several protein tyrosine kinases in MKN7 cells. Cells were treated for 3, 5, 15, 30, 60 and 240 min with 0.5 μM afatinib, 5 μg/ml trastuzumab or the combination of both. In the batch-corrected cluster analysis, the x-fold change of each activated protein is shown. Samples were clustered based on the similarity of the activated proteins and treatment conditions. Significant effects between different treatment conditions are indicated by (*) with increasing size (0.01 < *p*-value ≤0.05, 0.001 < *p*-value ≤0.01 and *p*-value ≤0.001). Increasing protein phosphorylation/activation is indicated in red. Blue indicates decreasing protein phosphorylation/activation. Abbreviations: Afa = afatinib, Tra = trastuzumab, untr = untreated

In MKN7 cells, trastuzumab did not alter the phosphorylation of investigated kinases regardless of treatment time. Afatinib treatment decreased the MEK1, MAPK3, p70S6K1 and AKT1 phosphorylation. Significant effects were shown for MEK1, MAPK3 and p70S6K1, which cluster apart from all other proteins. The comparison untreated vs. afatinib revealed strongest effects following 15–240 min treatment. Analogue to MKN1 cells, the combination of trastuzumab and afatinib resulted in a similar reduction in phosphorylated kinases when compared to afatinib alone (Fig. 4).

**Fig. 5:**
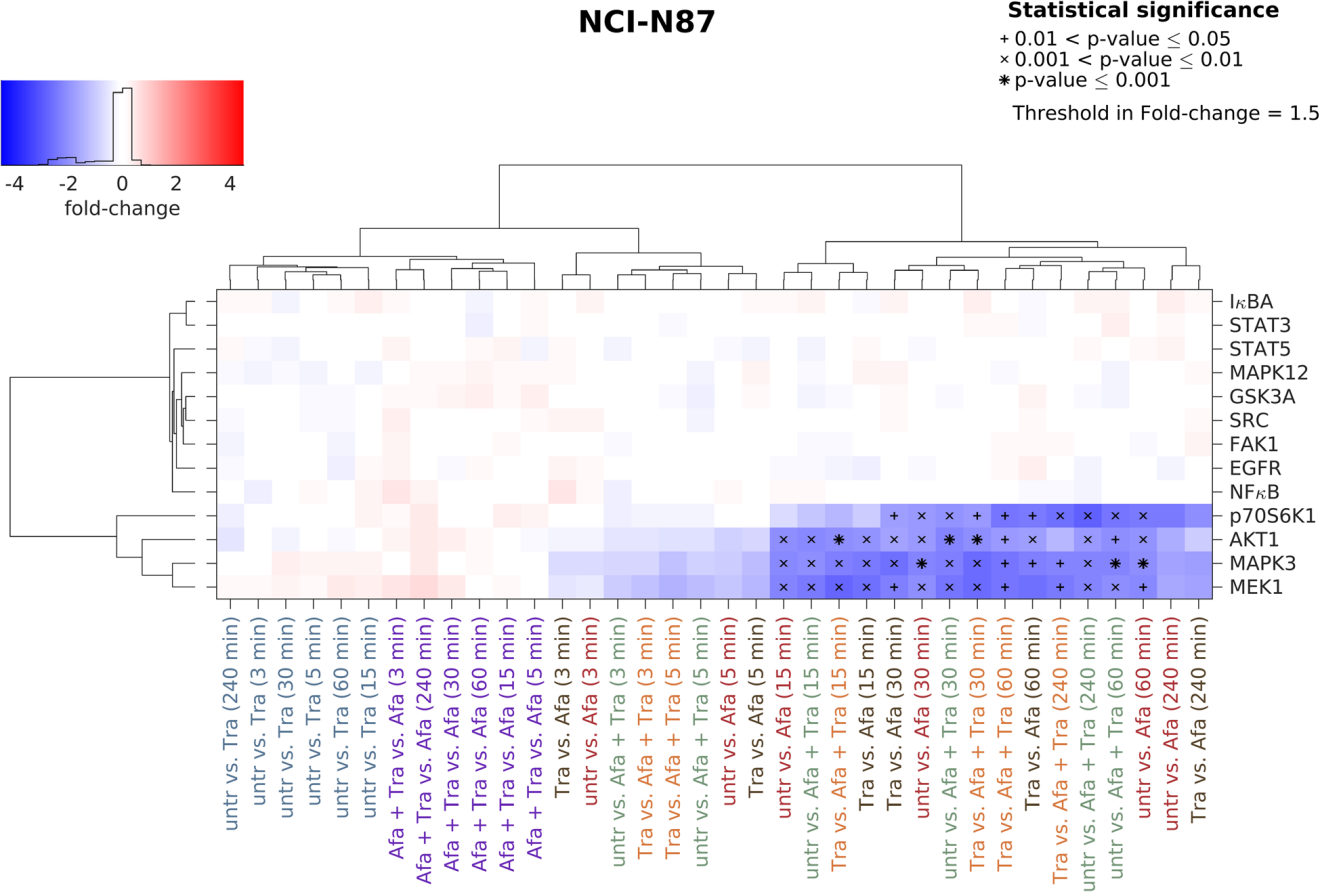
Effects of trastuzumab and afatinib on kinase phosphorylation in NCI-N87 cells. The effect of trastuzumab and/or afatinib on several protein tyrosine kinases in NCI-N87 cells was analyzed by Luminex technology. Cells were treated for 3, 5, 15, 30, 60 and 240 min with 0.5 μM afatinib, 5 μg/ml trastuzumab or the combination of both. In the batch-corrected cluster analysis, the x-fold change of each activated protein is shown. Samples were clustered based on the similarity of the activated proteins and treatment conditions. Significant effects between different treatment conditions are indicated by (*) with increasing size (0.01 < *p*-value ≤0.05, 0.001 < *p*-value ≤0.01 and *p*-value ≤0.001). Increasing protein phosphorylation/activation is indicated in red. Blue indicates decreasing protein phosphorylation/activation. Abbreviations: Afa = afatinib, Tra = trastuzumab, untr = untreated

As observed for MKN1 and MKN7 cells, trastuzumab did not change the phosphorylation of investigated kinases regardless of treatment time in NCI-N87 cells. Afatinib treatment resulted in significantly reduced phosphorylation of MEK1, MAPK3, AKT1 and p70S6K1, which cluster apart from all other proteins. By comparing untreated vs. afatinib strongest effects were observed following 15–60 min treatment. As observed for MKN1 and MKN7 cells, the combination of trastuzumab and afatinib was equally effective than afatinib alone (Fig. 5).

Decreasing effects of afatinib were observed in all three cell lines for AKT1, MAPK3, MEK1 and p70S6K phosphorylation. The cell line MKN1 showed the weakest effects, while MKN7 and NCI-N87 cells showed stronger effects following afatinib treatment. In MKN1 cells, MEK1 and MAPK3 were significantly reduced after afatinib treatment whereas MEK1, MAPK3, p70S6K1 were reduced in MKN7 cells and MEK1, MAPK3, p70S6K1 and AKT1 were reduced in NCI-N87 cells.

To confirm the results obtained by Luminex analysis the activation of AKT1, MAPK3, MEK1 and p70S6K1 in NCI-N87 cells was analyzed by western blot. The datasets were processed similar, thus a direct comparison of the two analyses is possible (Fig. S2, Fig. S3, Additional file 2). The blots were incubated with three antibodies at the same time. The antibodies were tested before with other samples to exclude unspecific bands. We chose this procedure since the amount of samples already used for Luminex analysis was limited.

In the Pearson correlation analysis, the time course effects observed by the two different methods were compared to each other (Table 1). Significant correlations were obtained for the activation of AKT1, MAPK3 and p70S6K. For the activation of MEK1, no significant correlation was observed.

**Table 1:** Pearson correlation analyses of Luminex and western blot results

Protein	pAKT1	pMAPK3	pMEK1	pp70S6K
Pearson correlation	0.8901	0.9862	0.6938	0.9288
p-value	0.007	0.00004	0.08	0.002

Especially for pAKT1 and pMEK1, the reduction after afatinib treatment was less pronounced in western blot analyses. This discrepancy may be explained by lower precision and reproducibility of western blot compared to Luminex analysis. However, the results were confirmed qualitatively.

### Cluster profiler analysis and significantly enriched pathways

Based on the differentially expressed genes after EGF, cetuximab, trastuzumab and afatinib treatment, the primary data analysis was performed as described below. Selected genes were validated by qPCR and DMSO was included as control (Figs. S4-S7 of Additional file 2, Additional file 3 and Table S1 Additional file 4).

The enriched pathways (Kyoto Encyclopedia of Genes and Genomes (KEGG), Reactome) and gene ontology terms (GO-terms) were determined for the differentially expressed genes after each treatment. Because no gene was regulated after trastuzumab treatment according to the selection criteria (log2-transformed fold-change > 1 or < − 1 and false discovery rate < 0.05), the functional enrichment analysis could not be performed. This analysis was used as basis for interpretation of drug effects in this manuscript. Additionally, a Cluster Profiler analysis, which compares the regulation of top 500 genes over all treatments in one cell line, was performed. The top 500 genes for each cell line and data set were selected according to fold-change and *p*-value. Subsequently, a Cluster Profiler analysis with functional enrichment analysis of the identified gene clusters was performed. Since this analysis is not mandatory in the context of this manuscript but may be of interest for some readers it is presented in Figs. S8-S11 of Additional file 2 and in Additional file 3.

#### Cetuximab

The functional enrichment analysis of differentially expressed genes for each treatment revealed important KEGG and Reactome pathways. Some KEGG signaling pathways (IL17, TNF, MAPK, chemokines) were significantly (p.adjust< 0.05) enriched after 4 h cetuximab treatment (Table S2, Additional file 4). The pathway transcriptional misregulation in cancer was enriched after 4 h and 24 h cetuximab treatment (Table S3, Additional file 4). The Reactome Pathways Chemokine receptor bind chemokines and Peptide ligand-binding receptors were enriched after 4 h cetuximab treatment (Table S4, Additional file 4). After 24 h of treatment, the analysis revealed no enriched Reactome pathways.

Some KEGG signaling pathways (IL17, TNF, Chemokines, ErbB) as well as cancer-associated pathways (Proteoglycans in cancer, Pathways in cancer, Bladder cancer, Breast cancer, Basal cell carcinoma) were enriched after 4 h EGF treatment (Table S5, Additional file 4). After 24 h EGF treatment, the pathways Transcriptional misregulation in cancer, Jak-STAT signaling pathway, PI3K-Akt signaling pathway and Proteoglycans in cancer were enriched (Table S6, Additional file 4). Regarding Reactome pathways, two G-protein related pathways (Signaling by GPCR, GPCR ligand binding) and the pathways Chemokine receptor bind chemokines and Peptide ligand-binding receptors were enriched after 4 h EGF treatment (Table S7, Additional file 4). The two G-protein related pathways were also enriched after 24 h EGF treatment. Furthermore, some pathways involving the extracellular matrix were enriched after 24 h EGF treatment (Degradation of the extracellular matrix, Collagen degradation, ECM proteoglycans, Extracellular matrix organization, Collagen formation) (Table S8, Additional file 4).

To summarize, following cetuximab treatment in MKN1 cells we identified important pathways based on the KEGG and Reactome databases. Amongst others, IL17, TNF, MAPK and chemokine signaling pathways were significantly enriched in response to cetuximab.

#### Afatinib

The functional enrichment analysis of differentially expressed genes revealed important KEGG and Reactome pathways in case of afatinib. The KEGG pathways Cytokine-cytokine receptor interaction, TNF signaling pathway and salmonella infection were significantly enriched in NCI-N87 cells after 4 h afatinib treatment (p.adjust < 0.05) (Table S9, Additional file 4). After 24 h afatinib treatment, pathways related to DNA/RNA and cell cycle were significantly enriched (e.g. RNA transport, Cell cycle, purine/pyrimidine metabolism, DNA replication, RNA polymerase) (Table S10, Additional file 4). No Reactome pathways were enriched after 4 h afatinib treatment in NCI-N87 cells. After 24 h afatinib treatment, 179 Reactome pathways were significantly enriched. The Top 20 of them were all cell cycle-related pathways (e.g. Cell Cycle, Mitotic G1-G1/S phases, Mitotic Metaphase and Anaphase, DNA Replication) (Table S11, Additional file 4).

In MKN1 cells, more KEGG pathways were enriched after 4 h than after 24 h. After 4 h treatment, these were some signaling pathways (TNF signaling pathway, IL-17 signaling pathway, MAPK signaling pathway, NF-kappa B signaling pathway, chemokine signaling pathway) and cancer-associated pathways (Pathways in cancer, Transcriptional misregulation in cancer, MicroRNAs in cancer) (Table S12, Additional file 4). After 24 h afatinib treatment only the pathway Hematopoietic cell lineage was enriched (Table S13, Additional file 4). After 4 h afatinib treatment in MKN1 cells some Reactome signaling pathways, especially Toll-like receptor signaling, were enriched (e.g. ERK/MAPK targets, Toll-like receptor Cascades, MyD88:Mal cascade initiated on plasma membrane) (Table S14, Additional file 4). The significance of the Toll-like receptor cascades is mainly related to the regulation of DUSPs (dual specificity phosphatases DUSP4, DUSP6, DUSP7), but also TLR4. After 24 h only the pathway PPARA activates gene expression was enriched (Table S15, Additional file 4).

Two KEGG signaling pathways (Jak-STAT signaling pathway, TNF signaling pathway) were enriched after 4 h afatinib treatment in MKN7 cells (Table S16, Additional file 4). After 24 h afatinib treatment cell cycle pathways (Cell cycle, DNA replication) and the pathway Ribosome biogenesis in eukaryotes were enriched (Table S17, Additional file 4). No Reactome pathways were enriched in MKN7 cells after 4 h afatinib treatment. After 24 h of treatment cell cycle pathways (e.g. Cell cycle, DNA Replication, G1/S transition, S Phase) were enriched (Table S18, Additional file 4).

#### Comparison of cetuximab and afatinib

The IL17, TNF, MAPK and chemokine signaling pathways were enriched in MKN1 cells after 4 h of cetuximab and afatinib treatment. NF-kappa B, TLR and MyD88 signaling were enriched after afatinib, but not cetuximab treatment. Moreover, cell cycle pathways were enriched in response to afatinib treatment in NCI-N87 and MKN7 but not in MKN1 cells. Table 2 summarizes a selection of KEGG and Reactome signaling and cell cycle pathways for all treatments and cell lines whereas all significantly enriched pathways are shown in Tables S2-S18 (Additional file 4).

**Table 2:** Selected significantly enriched signaling and cell cycle pathways (KEGG and Reactome)

ID	Description	GeneRatio	BgRatio	p.adjust
MKN1 4 h Cet vs. MKN1 4 h untr				
hsa04657	IL-17 signaling pathway	6/43	79/5844	0.00121677
hsa04668	TNF signaling pathway	6/43	102/5844	0.00259938
hsa04010	MAPK signaling pathway	8/43	272/5844	0.01366521
hsa04062	Chemokine signaling pathway	6/43	145/5844	0.01366521
NCI-N87 4 h Afa vs. NCI-N87 4 h untr				
hsa04668	TNF signaling pathway	11/147	102/5844	0.00964085
NCI-N87 24 h Afa vs. NCI-N87 24 h untr				
1,640,170	Cell Cycle	262/1891	462/5594	4.5934E-23
69,278	Cell Cycle, Mitotic	221/1891	380/5594	3.1704E-21
453,279	Mitotic G1-G1/S phases	81/1891	114/5594	8.6401E-14
69,242	S Phase	78/1891	110/5594	2.8328E-13
69,306	DNA Replication	68/1891	92/5594	5.238E-13
MKN1 4 h Afa vs. MKN1 4 h untr				
hsa04668	TNF signaling pathway	10/66	102/5844	2.3777E-05
hsa04657	IL-17 signaling pathway	8/66	79/5844	0.00011952
hsa04010	MAPK signaling pathway	11/66	272/5844	0.00427566
hsa04064	NF-kappa B signaling pathway	5/66	82/5844	0.02329389
hsa04621	NOD-like receptor signaling pathway	6/66	140/5844	0.0343651
hsa04062	Chemokine signaling pathway	6/66	145/5844	0.03858289
168,898	Toll-Like Receptors Cascades	6/60	105/5594	0.0159905
975,155	MyD88 dependent cascade initiated on endosome	4/60	65/5594	0.04380237
975,871	MyD88 cascade initiated on plasma membrane	4/60	65/5594	0.04380237
MKN7 4 h Afa vs. MKN7 4 h untr				
hsa04668	TNF signaling pathway	10/157	102/5844	0.02646242
hsa04630	Jak-STAT signaling pathway	14/157	125/5844	0.00112593
MKN7 24 h Afa vs. MKN7 24 h untr				
453,279	Mitotic G1-G1/S phases	40/764	114/5594	3.6681E-06
69,278	Cell Cycle, Mitotic	91/764	380/5594	6.4007E-06
69,206	G1/S Transition	34/764	94/5594	7.6198E-06
113,510	E2F mediated regulation of DNA replication	16/764	28/5594	1.4565E-05
1,640,170	Cell Cycle	103/764	462/5594	1.4565E-05

### Phenotypic treatment effects and related biological functions

As we were interested in the relation between phenotypic effects and altered biological functions following treatment, we aimed to identify genes whose changes in gene expression correlated with an altered phenotypic behavior. Therefore, we assessed the influence of trastuzumab and afatinib treatment on cell motility and apoptosis and we investigated the effect of cetuximab on apoptosis. The influence of cetuximab on cell motility was published elsewhere [35].

**Fig. 6:**
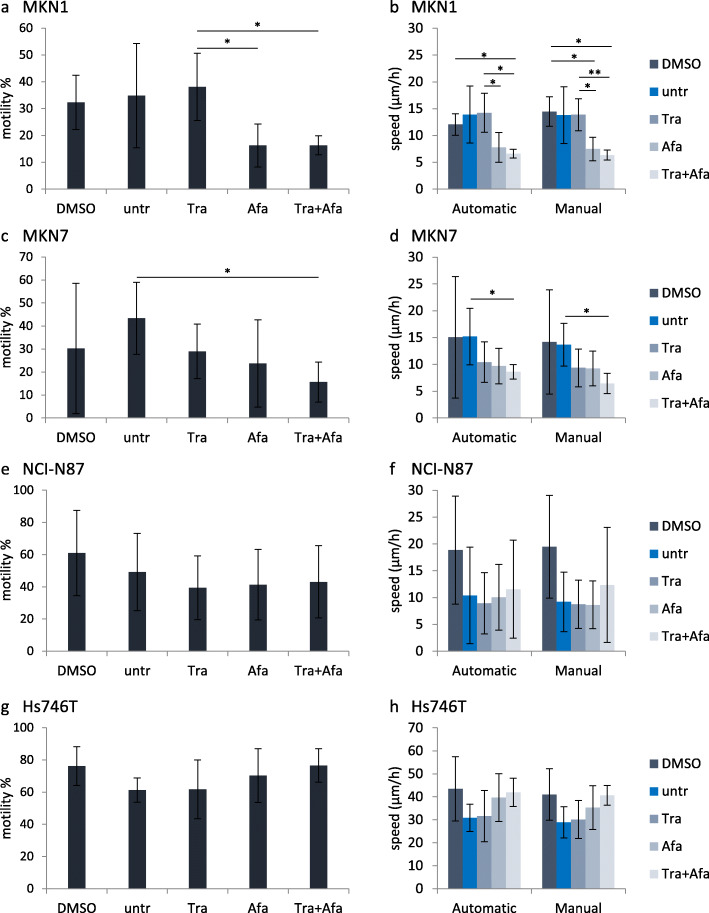
Effects of trastuzumab and afatinib on cell motility and average speed. MKN1 (**a, b**), MKN7 (**c, d**) NCI-N87 (**e, f**) and Hs746T (**g, h**) cells were treated with 5 μg/ml trastuzumab (Tra), 0.5 μM afatinib (Afa), 5 μg/ml trastuzumab + 0.5 μM afatinib (Tra + Afa) or afatinib solvent DMSO (0.05%). Untreated (untr) cells were used as control. Time-lapse microscopy was carried out for 7 h. Cell movement was tracked to assess motility (**a, c, e, g**) and average speed (**b, d, f, h**). Shown are the mean values of three to six experiments with standard deviation. Statistically significant effects are indicated by *p < 0.05 or **p < 0.01 (two-sample t-test)

**Fig. 7:**
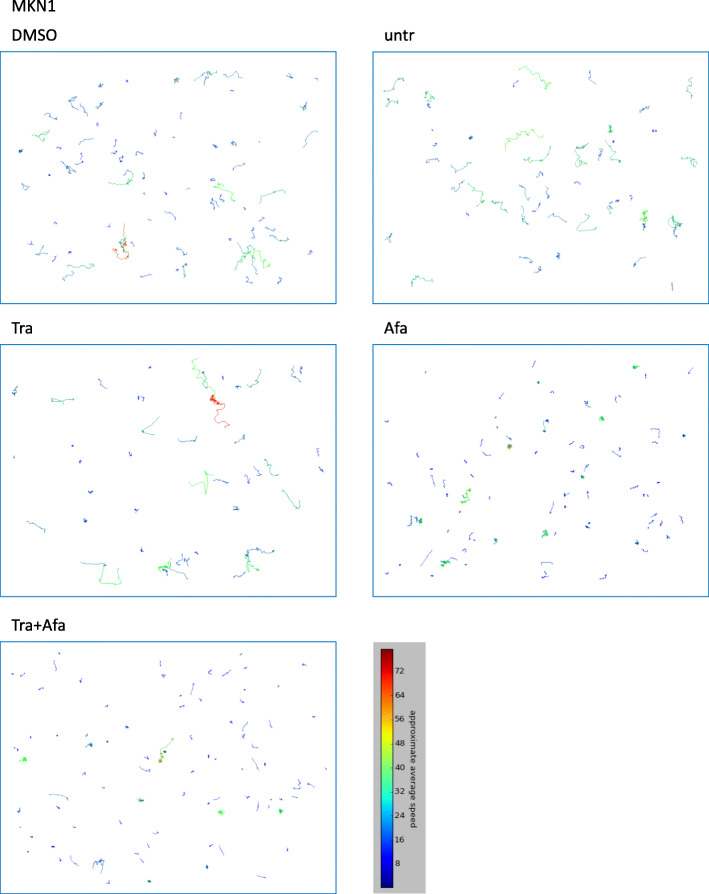
Trajectories of MKN1 cells treated with trastuzumab or afatinib. MKN1 cells were treated with 5 μg/ml trastuzumab (Tra), 0.5 μM afatinib (Afa), 5 μg/ml trastuzumab + 0.5 μM afatinib (Tra + Afa) or afatinib solvent DMSO (0.05%). Untreated (untr) cells were used as control. Cell movement was tracked for 7 h to assess approximate average speed. The trajectories of one exemplary film for each condition are shown. The trajectories were color-coded for approximate average speed

A cell was defined as motile when it completely left the field previously occupied by the cell itself. Significant effects of the different treatments on motility and average speed were observed in MKN1 and MKN7 cells, but not in Hs746T and NCI-N87 cells. The percentage of motile MKN1 cells was slightly reduced after afatinib and trastuzumab + afatinib treatment compared to untreated, DMSO-treated and trastuzumab-treated (Fig. 6a). DMSO, trastuzumab, afatinib and trastuzumab + afatinib treatments slightly reduced the number of motile MKN7 cells compared to untreated (Fig. 6c). No significant effect on motility was observed in NCI-N87 (Fig. 6e) and Hs746T cells (Fig. 6g). By measuring the average speed, the manual and the automatic assessments were compared. As indicated in Fig. 6, the two analysis methods resulted in similar levels of average speed. The treatments afatinib and trastuzumab + afatinib reduced the average speed in MKN1 cells compared to untreated, DMSO-treated or trastuzumab-treated cells, as assessed with both methods. However, significance equal to *p* < 0.05 was not reached in every case (Fig. 6b). Trastuzumab, afatinib and trastuzumab + afatinib slightly reduced the average speed compared to untreated MKN7 cells. Only the comparison between untreated and trastuzumab + afatinib reached significance with both methods (Fig. 6d). The average speed of NCI-N87 was not significantly influenced by any of the treatments. This might be due to high standard deviations in all conditions, questioning the suitability of NCI-N87 cells for motility analyses (Fig. 6f). In Hs746T cells, the DMSO-containing treatments (DMSO, afatinib and trastuzumab + afatinib) slightly increased average speed (Fig. 6h). To visualize the cell movement of one representative film per condition, the trajectory of each cell was color-coded according to its approximate average speed. The results for MKN1 cells are depicted exemplarily (Fig. 7) while the results for MKN7, NCI-N87 and Hs746T are presented in Additional file 2 (Figs. S12 - S14).

In order to identify genes that are potentially involved in the regulation of motility by afatinib, the genes that were regulated after 4 h afatinib or trastuzumab + afatinib treatment in MKN1, MKN7 and NCI-N87 cells and were assigned to the gene ontology biological function “positive regulation of cell motility” were isolated. The 4 h treatment was chosen because of the film length of 7 h. This biological function was significantly enriched in MKN1 cells after afatinib and trastuzmab + afatinib treatment, whereas adjusted *p*-values (p.adjust) of 0.0548 (Afa) and 0.0747 (Tra + Afa) for MKN7 cells and 0.223 (Afa) and 0.0767 (Tra + Afa) for NCI-N87 cells were observed (Table 3).

**Table 3:** Regulation of biological function “positive regulation of cell motility” by afatinib and trastuzumab + afatinib

Condition	ID	Description	GeneRatio	BgRatio	p.adjust
MKN1 4 h Afa vs. MKN1 4 h untr	GO:2000147	positive regulation of cell motility	18/112	406/14021	3.63E-07
MKN1 4 h Tra + Afa vs. MKN1 4 h untr	GO:2000147	positive regulation of cell motility	15/112	406/14021	6.64E-05
MKN7 4 h Afa vs. MKN7 4 h untr	GO:2000147	positive regulation of cell motility	21/365	406/14021	5.48E-02
MKN7 4 h Tra + Afa vs. MKN7 4 h untr	GO:2000147	positive regulation of cell motility	22/410	406/14021	7.47E-02
NCI-N87 4 h Afa vs. NCI-N87 4 h untr	GO:2000147	positive regulation of cell motility	17/337	406/14021	2.23E-01
NCI-N87 4 h Tra + Afa vs. NCI-N87 4 h untr	GO:2000147	positive regulation of cell motility	19/326	406/14021	7.67E-02

**Fig. 8:**
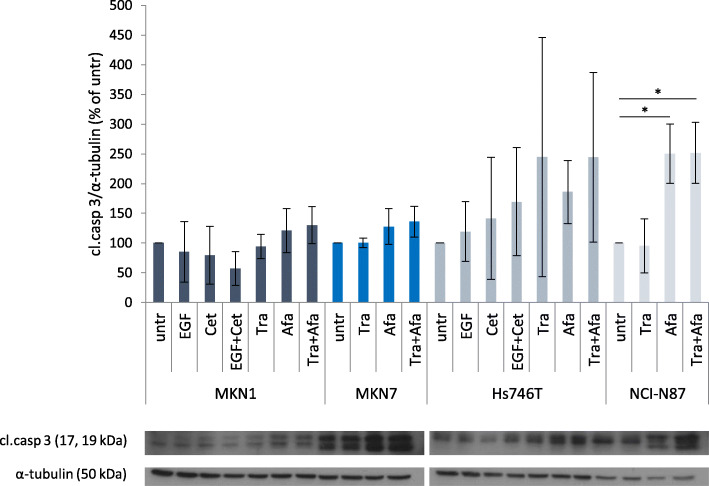
Effects of EGF, cetuximab, trastuzumab and afatinib treatment on caspase 3 activation. MKN1 and Hs746T cells were treated with EGF, EGF + cetuximab (EGF + Cet), cetuximab (Cet), trastuzumab (Tra), afatinib (Afa) or trastuzumab + afatinib (Tra + Afa) for 24 h. MKN7 and NCI-N87 were treated with trastuzumab (Tra), afatinib (Afa) or trastuzumab + afatinib (Tra + Afa) for 24 h. Cell lysates were analyzed by western blot with antibodies against cleaved (cl.) caspase 3 (Asp175). The mean of three biological experiments with standard deviation is shown. Significant effects compared to untreated are indicated by *p < 0.05 (one-sample t-test). Full-length blots are presented in Additional file 5

The activation of Caspase 3 was measured as marker for apoptosis. Treatment with afatinib and trastuzumab + afatinib for 24 h increased Caspase 3 activation in NCI-N87 but not in MKN1, MKN7 and Hs746T cells (Fig. 8). Thus, afatinib induced apoptosis only in NCI-N87 cells. The pathway “positive regulation of apoptotic signaling pathway” was significantly enriched in NCI-N87 cells following trastuzumab + afatinib treatment. This pathway was not significantly enriched in case of 24 h afatinib treatment in NCI-N87 cells, but the p-value was with 0.141 much lower than for MKN1 and MKN7 cells (Table 4).

**Table 4:** Regulation of biological function “positive regulation of apoptotic signaling pathway” by cetuximab, afatinib and trastuzumab + afatinib

Condition	ID	Description	GeneRatio	BgRatio	p.adjust
MKN1 24 h Cet vs. MKN1 24 h untr	GO:2001235	positive regulation of apoptotic signaling pathway	2/162	156/14021	6.82E-01
MKN1 24 h Afa vs. MKN1 24 h untr	GO:2001235	positive regulation of apoptotic signaling pathway	5/463	156/14021	7.57E-01
MKN1 24 h Tra + Afa vs. MKN1 24 h untr	GO:2001235	positive regulation of apoptotic signaling pathway	5/483	156/14021	8.11E-01
MKN7 24 h Afa vs. MKN7 24 h untr	GO:2001235	positive regulation of apoptotic signaling pathway	15/1744	156/14021	9.87E-01
MKN7 24 h Tra + Afa vs. MKN7 24 h untr	GO:2001235	positive regulation of apoptotic signaling pathway	17/1781	156/14021	9.59E-01
NCI-N87 24 h Afa vs. NCI-N87 24 h untr	GO:2001235	positive regulation of apoptotic signaling pathway	62/4359	156/14021	1.41E-01
NCI-N87 24 h Tra + Afa vs. NCI-N87 24 h untr	GO:2001235	positive regulation of apoptotic signaling pathway	72/4772	156/14021	1.75E-02

Next, the genes regulated after 4 h afatinib and trastuzumab + afatinib treatment in MKN1 cells assigned to “positive regulation of cell motility” were compared. MKN1 cells were chosen for this analysis because of the clear effects of afatinib in motility analysis. The genes *SERPINE1*, *F3*, *CXCL8*, *PLPP3*, *F2RL1*, *PTGS2*, *CYR61*, *CXCL1*, *SEMA6D*, *ETS1*, *HBEGF*, *ITGA2*, *HAS2* and *SPRY2* were regulated in MKN1 cells after afatinib and trastuzumab + afatinib treatment (Table 5, Table S19, Additional file 4). Forty-four genes, such as BAX, BBC3 or FAS, which were assigned to the “positive regulation of apoptotic signaling pathway” were regulated by afatinib and trastuzumab + afatinib in NCI-N87 cells, but not in MKN1 or MKN7 cells (Table 5, Table S20, Additional file 4).

**Table 5:** Candidate genes involved in phenotypic response to afatinib treatment

**a) Genes potentially involved in reduction of motility after afatinib treatment**									
*SERPINE1* *HBEGF*	*F3* *ITGA2*	*CXCL8* *HAS2*	*PLPP3* *SPRY2*	*F2RL1*	*PTGS2*	*CYR61*	*CXCL1*	*SEMA6D*	*ETS1*
**b) Genes potentially involved in induction of apoptosis after afatinib treatment**									
*BAX*	*BBC3*	*BCLAF1*	*CAV1*	*E2F1*	*FADD*	*FAF1*	*FAS*	*GSN*	*HYAL2*
*IL19*	*IL20RA*	*INHBB*	*LCK*	*LGALS9*	*NACC2*	*NF1*	*NFATC4*	*NKX3-1*	*PAK2*
*PARK7*	*PDCD5*	*PDIA3*	*PEA15*	*PPIF*	*PPP2R1B*	*PPP3CC*	*PRKRA*	*SFN*	*SFPQ*
*SKIL*	*SLC9A3R1*	*SMAD3*	*STK4*	*TGFBR1*	*TP73*	*TPD52L1*	*YWHAB*	*YWHAE*	*YWHAG*
*YWHAH*	*YWHAQ*	*YWHAZ*	*ZNF205*						

Genes that were regulated in MKN1 cells after 4 h afatinib and trastuzumab + afatinib treatment and were assigned to the biological function “positive regulation of cell motility” were selected (a). Genes that were regulated in NCI-N87 but not in MKN1 and MKN7 cells after 24 h afatinib and trastuzumab + afatinib treatment and were assigned to the biological function “positive regulation of apoptotic signaling pathway” were selected (b). Green indicates downregulation and red upregulation

**Fig. 9:**
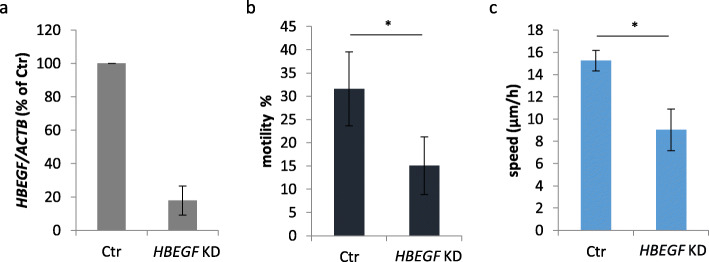
Effect of *HBEGF* knockdown on motility and average speed. MKN1 cells were transfected with negative-control (Ctr) or *HBEGF* (*HBEGF* KD) siRNA. The knockdown was checked on RNA level on day 1 after transfection (**a**). Time-lapse microscopy was carried out for 7 h. Cell movement was tracked to assess motility (**b**) and average speed (manual) (**c**). Shown are the mean values of four experiments with standard deviation. Statistically significant effects are indicated by *p < 0.05 (two-sample t-test) (**b, c**)

To verify the hypothesis that the genes listed in Table 5 are involved in regulation of motility following afatinib treatment, we tested the effect of *HBEGF* knockdown on motility parameters in MKN1 cells. Off-target effects of the used siRNAs cannot be excluded but are minimized by the use of 4 different siRNAs and Qiagens innovative siRNA design. HBEGF was chosen because of its function as EGFR-family ligand and research interests of our group. Since *HBEGF* was downregulated by afatinib treatment we expected similar effects on motility after *HBEG*F knockdown. *HBEGF* gene expression was reduced to 18% (Fig. 9a). The percentage of motile cells and average speed were significantly decreased in *HBEGF* KD compared to control cells (Fig. 9b and c).

In summary, we demonstrated reduced average speed and motility in MKN1 and MKN7 cells and induction of apoptosis in NCI-N87 cells after afatinib-containing treatments. Following afatinib treatment we identified genes that might be involved in the regulation of motility and apoptosis. *HBEGF* was confirmed as regulator of cell motility by knockdown experiments.

## Discussion

### Intracellular kinases were regulated by cetuximab and afatinib treatment

We previously observed effects of trastuzumab on HER2 activation but not on intracellular kinases [21]. We hypothesized that effects on intracellular kinases may be visible after prolonged or shortened treatment with trastuzumab. However, none of the 12 analyzed kinases was altered at any time point. Other receptor tyrosine kinases, such as HER3 or EGFR, may compensate the reduced activation of HER2 and sustain activation of intracellular kinases [12–14, 43].

The EGFR antibody cetuximab and the pan-HER tyrosine kinase inhibitor afatinib showed stronger effects on intracellular signaling. Cetuximab mainly affected MAPK3 and MEK1 whereas afatinib affected additionally AKT1 and p70S6K1, depending on the cell line. MAPK3 and MEK1 are part of the RAS/MAPK pathway and AKT01 and p70S6K are important kinases of the PI3K/AKT pathway. These two pathways regulate proliferation, survival, differentiation and motility of cancer cells and are known to be affected by anti-HER therapies [44].

### Effects of trastuzumab, cetuximab and afatinib on signaling and cell cycle pathways

Significantly enriched KEGG and Reactome pathways were identified based on the gene expression analysis with following functional enrichment analysis. During this work we focused on signaling and cell cycle pathways. In case of trastuzumab the functional enrichment analysis was not performed since no gene was regulated according to the selection criteria. Amongst others, the IL17, TNF, MAPK and chemokine signaling pathways were enriched after cetuximab treatment in MKN1 cells. Following afatinib treatment, the TNF signaling pathway was enriched in NCI-N87, MKN1 and MKN7 cells. The Jak-STAT signaling was exclusively enriched in MKN7 cells. IL17, MAPK, NF-kappa B, NOD-like receptor, chemokine, TLR and MyD88 signaling pathways were enriched in MKN1 cells after afatinib treatment. The cell line MKN1 can be used to directly compare the effects of cetuximab and afatinib. IL17, TNF, MAPK and chemokine signaling pathways were enriched in MKN1 cells after cetuximab and afatinib treatment, whereas NF-kappa B, TLR and MyD88 signaling were enriched after afatinib treatment only. Thus, pathways related to the immune system are important for cetuximab and afatinib response in MKN1 cells. Processes related to immune response or inflammation were also important in head and neck squamous-cell carcinoma (HNSCC) cell lines after 48 h erlotinib (EGFR inhibitor) treatment. The functional analysis suggested an activation of inflammatory pathways that might be mediated by MyD88. Further experiments suggested the IL-1α/IL-1R/MYD88/IL-6 pathway as responsible for the reduced anti-tumor efficacy of erlotinib and other EGFR inhibitors [23]. However, in HNSCC cells these processes were upregulated, whereas in our experiments after 24 h treatment most genes involved in these pathways were downregulated. The upregulation may be part of a resistance mechanism starting after 48 h of treatment.

Both, KEGG and Reactome pathway analyses showed a cell cycle regulation after 24 h afatinib treatment in NCI-N87 and MKN7 cells, but not in MKN1 cells. When the list of genes within the Reactome Pathway Cell Cycle in MKN7 (103 genes) and NCI-N87 (262 genes) cells was compared with the list of all afatinib-regulated genes in MKN1 cells, only two genes were present in both lists: *CCND1, TERT*. Afatinib may have another, cell cycle-independent mechanism of action in MKN1 cells. However, it is also possible that the effect on the cell cycle in MKN1 cells is visible only after prolonged afatinib treatment. Afatinib-induced cell cycle arrest was measured by flow cytometry in various cell lines including HNSCC, pancreatic carcinoma, colorectal carcinoma, esophageal squamous carcinoma, gastric carcinoma, ovarian carcinoma and oral squamous carcinoma [25, 28, 30, 32, 45–49]. In gastric carcinoma cells a sub-G1/G1 cell cycle arrest was detected in NCI-N87 and SNU216 after afatinib treatment, whereas SNU668 cells showed no changes in cell cycle distribution [32]. Hence, the enrichment of cell cycle pathways identified by functional enrichment analysis might be an indicator for cell cycle arrest.

In summary, we identified important signaling pathways for cetuximab and afatinib response and showed involvement of immune-related pathways by gene expression analysis. Furthermore, we demonstrated gene expression changes in cell cycle pathways in MKN7 and NCI-N87, but not in MKN1 cells.

### Regulation of cell motility and apoptosis after afatinib and trastuzumab treatment

Treatment with afatinib as well as with the combination of trastuzumab + afatinib reduced the motility and average speed of MKN1 cells. These effects were less clear in MKN7 cells. Motility was slightly reduced after trastuzumab, afatinib, trastuzumab + afatinib and DMSO treatment. The interpretation is difficult since high standard deviations were observed in DMSO- and afatinib-treated cells. Treatment with trastuzumab, afatinib and the combination of both slightly reduced the average speed of MKN7 cells. Taken together, these data show that afatinib-containing treatments reduced average speed and motility in MKN1 and MKN7 cells. From gene expression analysis and GO annotations we extracted those genes that were regulated by afatinib-containing treatments in MKN1 cells and were related to the biological function “positive regulation of cell motility”. As a result, the genes *SERPINE1*, *F3*, *CXCL8*, *PLPP3*, *F2RL1*, *PTGS2*, *CYR61*, *CXCL1*, *SEMA6D*, *ETS1*, *HBEGF*, *ITGA2*, *HAS2* and *SPRY2* could be involved in the regulation of cell motility after afatinib treatment. To confirm this hypothesis, we analyzed cell motility after knockdown of one of those genes. Indeed, average speed was reduced after knockdown of *HBEGF* in MKN1 cells.

Motility and average speed were reduced after trastuzumab treatment in MKN7 cells only. However, there was no gene expression modulation under this condition. Therefore, in case of trastuzumab, the altered motility parameters cannot be explained by changes in gene expression. We hypothesize that the effect of trastuzumab on cell motility is caused by gene expression independent mechanisms. Reduced cell migration after afatinib treatment was observed in ovarian, pancreatic and breast cancer cell lines [25–27]. To our knowledge, we were the first to use time-lapse microscopy to show reduced motility and average speed following afatinib treatment.

Apoptosis was induced by afatinib-containing treatments in NCI-N87 cells only. From the gene expression analysis and GO annotations we isolated genes that were regulated by afatinib-containing treatments in NCI-N87 cells and were related to the biological function “positive regulation of apoptotic signaling pathway”. Although this pathway was only significantly enriched following trastzuzumab + afatinib and not afatinib treatment, the *p*-value was the lowest in NCI-N87 cells after afatinib and trastuzumab + afatinib treatment. Thus, the 44 extracted genes might be involved in induction of apoptosis after afatinib treatment in NCI-N87 cells**.** The lacking significance of the biological function “positive regulation of apoptotic signaling pathway” after afatinib treatment is of minor importance for this approach since this analysis was mainly used to identify genes involved in apoptosis. The apoptotic effect of afatinib is well known from experiments with various cancer cell lines, including head and neck squamous cell carcinoma, lung carcinoma, esophageal squamous carcinoma, neuroblastoma, gastric carcinoma, bladder carcinoma and intrahepatic cholangiocarcinoma [28–34].

Overall, the phenotypic changes observed after afatinib treatment were reflected by altered biological functions of the gene ontology database. We suggested a list of 14 genes that might be involved in reduction of motility after afatinib treatment. The importance of one of these genes, *HBEGF*, was validated by means of motility analysis after knockdown. We also identified 44 genes that might have a role in the induction of apoptosis following afatinib treatment.

### Different effects of anti-HER therapies on intracellular signaling, gene expression and phenotype

Trastuzumab treatment did not affect intracellular signaling, cell motility and apoptosis and showed only small effects on gene expression in the analyzed cell lines. Following cetuximab treatment, the RAS/MAPK pathway was altered on phosphoprotein level, the IL-17, TNF, MAPK and chemokine signaling pathways were influenced on gene expression level and the cell motility (described in [35]) but not apoptosis was affected. Strongest effects on all analyzed levels were observed after afatinib treatment. These include inhibition of the RAS/MAPK and PI3K/AKT signaling on phosphoprotein level, the changes in TNF, IL-17, MAPK, NF-kappaB, NOD-like receptor, chemokine, Toll-like receptor, MyD88 and Jak-STAT signaling and cell cycle pathways on gene expression level and the regulation of cell motility and apoptosis. Thus, from a molecular point of view, afatinib is most effective and therefore a promising candidate for therapy of gastric cancer patients. However, the well-known antibody-dependent cell-mediated cytotoxicity (ADCC) effects of trastuzumab and cetuximab, which are mediated by immune cells, are not considered in our analyses [50–52].

## Conclusions

The effects of cetuximab, trastuzumab and afatinib on activation of intracellular kinases and gene expression in gastric cancer cell lines were compared. Analysis of intracellular kinase activations revealed effects of cetuximab and afatinib on MAPK3, MEK1, AKT and p70S6K1 with differences regarding effect strength between cell lines and treatments. Based on gene expression analysis, we identified important signaling and immune-related pathways for cetuximab and afatinib response. Moreover, cell cycle pathways were identified as important for afatinib response. In order to identify genes that are involved in the regulation of motility and apoptosis after afatinib treatment, we analyzed the effects of afatinib on motility by time-lapse microscopy and on apoptosis by cleaved Caspase 3 staining. The phenotypic changes were reflected by altered biological functions of the gene ontology database. We suggested a list of 14 genes that might be involved in the reduction of motility, and a list of 44 genes that might be involved in the induction of apoptosis after afatinib treatment. Our findings were validated by assessment of motility parameters after *HBEGF* knockdown.

## Supplementary information

**Additional file 1.** Supplemental Method.

**Additional file 2.** Supplemental Figures.

**Additional file 3.** Supplemental Results.

**Additional file 4.** Supplemental Tables.

**Additional file 5.** Supplemental full-length blots corresponding to Fig. 8.

**Additional file 6.** Supplemental full-length blots corresponding to Additional file 2, Fig. S3.

## Data Availability

The datasets generated and analysed during the current study are available in the GEO repository (Accession GSE141352), https://www.ncbi.nlm.nih.gov/921geo/.

## Abbreviations

ADCC: Antibody-dependent cell-mediated cytotoxicity
Afa: Afatinib
Cet: Cetuximab
Cl.casp3: Cleaved caspase 3
DMSO: Dimethylsulfoxid
FDR: False discorey rate, adjusted *p*-value
GO-term: Gene ontology term
HNSCC: Head and neck squamous-cell carcinoma
KEGG: Kyoto Encyclopedia of Genes and Genomes
log2FC: Log2-transformed fold-change
p.adjust: Adjusted *p*-value
Tra: Trastuzumab
Untr: Untreated
