# Correction to: Determining the effects of trastuzumab, cetuximab and afatinib by phosphoprotein, gene expression and phenotypic analysis in gastric cancer cell lines

**DOI:** 10.1186/s12885-020-07642-2

**Published:** 2020-11-23

**Authors:** Karolin Ebert, Gwen Zwingenberger, Elena Barbaria, Simone Keller, Corinna Heck, Rouven Arnold, Vanessa Hollerieth, Julian Mattes, Robert Geffers, Elba Raimúndez, Jan Hasenauer, Birgit Luber

**Affiliations:** 1 Fakultät für Medizin, Technische Universität München, Klinikum rechts der Isar, Institut für Allgemeine Pathologie und Pathologische Anatomie, 81675 München, Germany; 2 MATTES Medical Imaging GmbH, A-4232 Hagenberg, Austria; 3 Helmholtz Zentrum für Infektionsforschung, 38124 Braunschweig, Germany; 4 Helmholtz Zentrum München-German Research Center for Environmental Health, Institute of Computational Biology, 85764 Neuherberg, Germany; 5 Center for Mathematics, Technische Universität München, 85748 Garching, Germany; 6 Faculty of Mathematics and Natural Sciences, University of Bonn, 53113 Bonn, Germany

**Correction to: BMC Cancer 20, 1039 (2020)**

**https://doi.org/10.1186/s12885-020-07540-7**

Following publication of the original article [1], the authors reported an error in the labeling of Table 5. The corrected Table 5 is given below.

**Table 5:** Candidate genes involved in phenotypic response to afatinib treatment

**a) Genes potentially involved in reduction of motility after afatinib treatment**									
*SERPINE1* *HBEGF*	*F3* *ITGA2*	*CXCL8* *HAS2*	*PLPP3* *SPRY2*	*F2RL1*	*PTGS2*	*CYR61*	*CXCL1*	**SEMA6D**	*ETS1*
**b) Genes potentially involved in induction of apoptosis after afatinib treatment**									
*BAX*	**BBC3**	*BCLAF1*	*CAV1*	*E2F1*	*FADD*	*FAF1*	*FAS*	**GSN**	*HYAL2*
*IL19*	*IL20RA*	**INHBB**	*LCK*	*LGALS9*	*NACC2*	*NF1*	**NFATC4**	*NKX3–1*	*PAK2*
*PARK7*	*PDCD5*	*PDIA3*	*PEA15*	*PPIF*	*PPP2R1B*	*PPP3CC*	*PRKRA*	*SFN*	*SFPQ*
*SKIL*	*SLC9A3R1*	*SMAD3*	*STK4*	*TGFBR1*	*TP73*	*TPD52L1*	*YWHAB*	*YWHAE*	*YWHAG*
*YWHAH*	*YWHAQ*	*YWHAZ*	**ZNF205**						

Genes that were regulated in MKN1 cells after 4 h afatinib and trastuzumab + afatinib treatment and were assigned to the biological function “positive regulation of cell motility” were selected (a). Genes that were regulated in NCI-N87 but not in MKN1 and MKN7 cells after 24 h afatinib and trastuzumab + afatinib treatment and were assigned to the biological function “positive regulation of apoptotic signaling pathway” were selected (b). *Italics typeface* indicates downregulation and **bold typeface** upregulation

The original article [1] has been corrected.
