# Supplementary material for: Determining the effects of trastuzumab, cetuximab and afatinib by phosphoprotein, gene expression and phenotypic analysis in gastric cancer cell lines
Source: BMC Cancer. 2020 Oct 28;20:1039. doi: 10.1186/s12885-020-07540-7 (PMC7594334; doi:10.1186/s12885-020-07540-7)
Supplement: Supplementary file 1 — Additional file 1. Supplemental Method. [file 12885_2020_7540_MOESM1_ESM.pdf]

Supplemental Methods

Cell lines

The cell lines MKN1, MKN7, NCI-N87 and Hs746T were established from metastatic sites of gastric carcinomas: cell line MKN1 was established from a lymph node metastasis of a gastric adenosquamous carcinoma; cell line MKN7 was derived from a lymph node metastasis of a gastric tubular adenocarcinoma; cell line NCI-N87 was established from a liver metastasis of a gastric tubular adenocarcinoma and cell line Hs746T was derived from a metastic site in the muscle of the left leg of a gastric adenocarcinoma.

Informations about cell lines MKN1 and MKN7 were obtained from Cell Bank RIKEN BioResource Center (https://cellbank.nibiohn.go.jp), about cell lines NCI-N87 and Hs746T from ATCC Cell Biology Collection (https://www.lgcstandards-atcc.org) and about all four cell lines from ExPASy Bioinformatics Resource Portal (https://www.expasy.org/). The internet research was performed on March 28, 2020.

Primary data analysis

Each FASTQ file gets a quality report generated by FASTQC tool (v. 0.11.3, default settings) (1). Before alignment to reference genome each sequence in the raw FASTQ files was trimmed on base call quality and sequencing adapter contamination using Trim Galore! wrapper tool (v.0.3.7, default settings) (2). Reads shorter than 20 bp were removed from the FASTQ file. Trimmed reads were aligned to the reference genome using open source short read aligner STAR (STAR_2.4.2a; RefGenome hg38 (UCSC-archive-2015-08-10), Illumina iGenome Reference; Transcriptome (genes.gtf, UCSC-archive-2015-08-10), Illumina iGenome Reference) (https://code.google.com/p/rna-star/) with settings according to log file (3). Feature counts were determined using R package "Rsubread" (4). Only genes showing counts greater 5 at least two times across all samples were considered for further analysis (data cleansing). Gene annotation was done by R package "bioMaRt" (5). Before starting the statistical analysis steps, expression data was log2 transformed and normalized according to 50th percentile (quartile normalization using edgeR). Differential gene expression was calculated by R package "edgeR" (6).

References

1. Andrews S. FastQC: a quality control tool for high throughput sequence data. http://www.bioinformatics.babraham.ac.uk/projects/fastqc. 2010.

2. Krueger F. Trim Galore!: A wrapper tool around Cutadapt and FastQC to consistently apply quality and adapter trimming to FastQ files, with some extra functionality for MspI-digested RRBS-type (Reduced Representation Bisufite-Seq) libraries. http://www.bioinformatics.babraham.ac.uk/projects/trim_galore/. 2012.
3. Dobin A, Davis CA, Schlesinger F, Drenkow J, Zaleski C, Jha S, et al. STAR: ultrafast universal RNA-seq aligner. Bioinformatics. 2013;29(1):15-21.
4. Liao Y, Smyth GK, Shi W. featureCounts: an efficient general purpose program for assigning sequence reads to genomic features. Bioinformatics. 2014;30(7):923-30.
5. Durinck S, Moreau Y, Kasprzyk A, Davis S, De Moor B, Brazma A, et al. BioMart and Bioconductor: a powerful link between biological databases and microarray data analysis. Bioinformatics. 2005;21(16):3439-40.
6. Robinson MD, McCarthy DJ, Smyth GK. edgeR: a Bioconductor package for differential expression analysis of digital gene expression data. Bioinformatics. 2010;26(1):139-40.
