# Supplementary material for: Determining the effects of trastuzumab, cetuximab and afatinib by phosphoprotein, gene expression and phenotypic analysis in gastric cancer cell lines
Source: BMC Cancer. 2020 Oct 28;20:1039. doi: 10.1186/s12885-020-07540-7 (PMC7594334; doi:10.1186/s12885-020-07540-7)
Supplement: Supplementary file 2 — Additional file 2. Supplemental Figures. [file 12885_2020_7540_MOESM2_ESM.pdf]

Figure S1

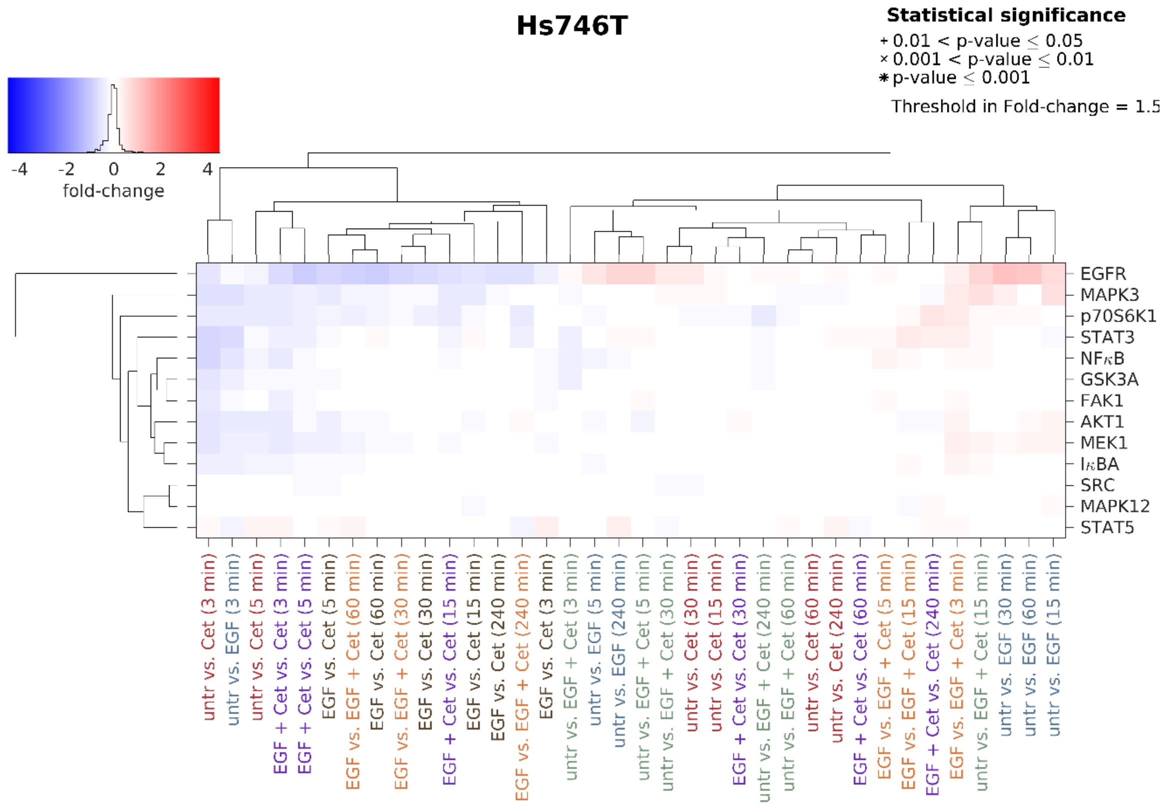

Figure S1. Effects of EGF and cetuximab on kinase phosphorylation in Hs746T cells.

Luminex analysis was performed to detect the effects on protein tyrosine kinases in Hs746T cells induced by EGF and/or cetuximab. Cells were treated for 3, 5, 15, 30, 60 and 240 minutes with 5 ng/ml EGF, 1 µg/ml cetuximab or the combination of both. In the batch-corrected cluster analysis, the x-fold change of each activated protein is shown. Samples were clustered based on to the similarity of the activated proteins and treatment conditions. Significant effects between different treatment conditions are indicated by (*) with increasing size (0.01 < p-value ≤ 0.05, 0.001 < p-value ≤ 0.01 and p-value ≤ 0.001). Increasing protein phosphorylation/activation is indicated in red. Blue indicates decreasing protein phosphorylation/activation. Abbreviations: Cet = cetuximab, untr = untreated.

Figure S2

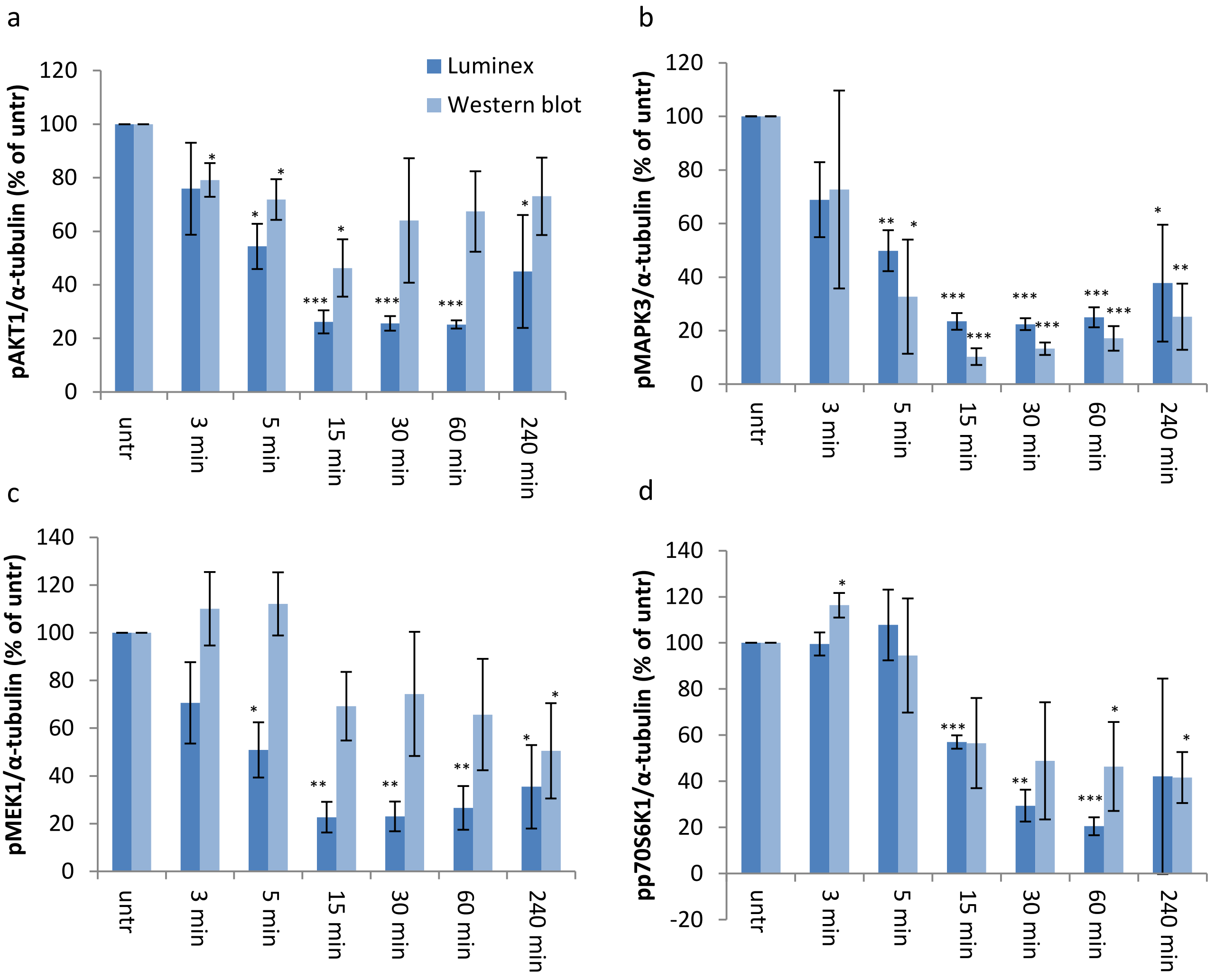

Figure S2: Validation of the Luminex analysis by Western blot.
The levels of activated tyrosine kinases pAKT1 (a), pMAPK3 (b), pMEK1 (c), pp70S6K1(d) were determined in NCI-N87 cells treated for 3, 5, 15, 30, 60 and 240 min with 0.5 µM afatinib (Afa). The mean values with standard deviation of three independent experiments are shown. Statistically significant effects compared to untreated are indicated by *p<0.05, **p<0.01 or ***p<0.01 (one-sample t-test).

Figure S3

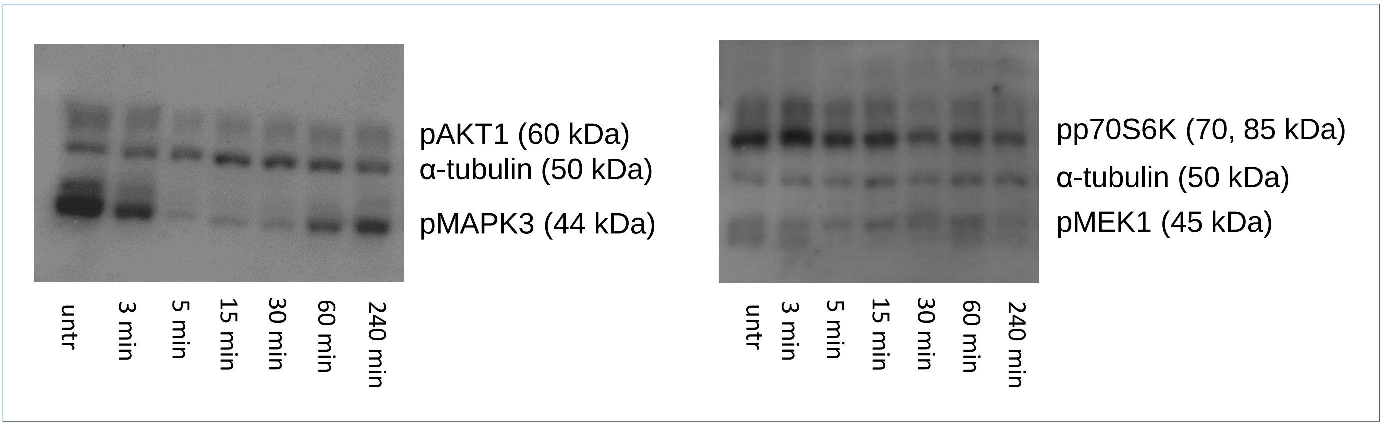

Figure S3: Western blot images for Luminex validation.
The levels of activated tyrosine kinases were determined in NCI-N87 cells treated for 3, 5, 15, 30, 60 and 240 min with 0.5 µM afatinib (Afa). The results of one representative experiment are shown. Full-length blots are presented in Additional file 6.

Figure S4

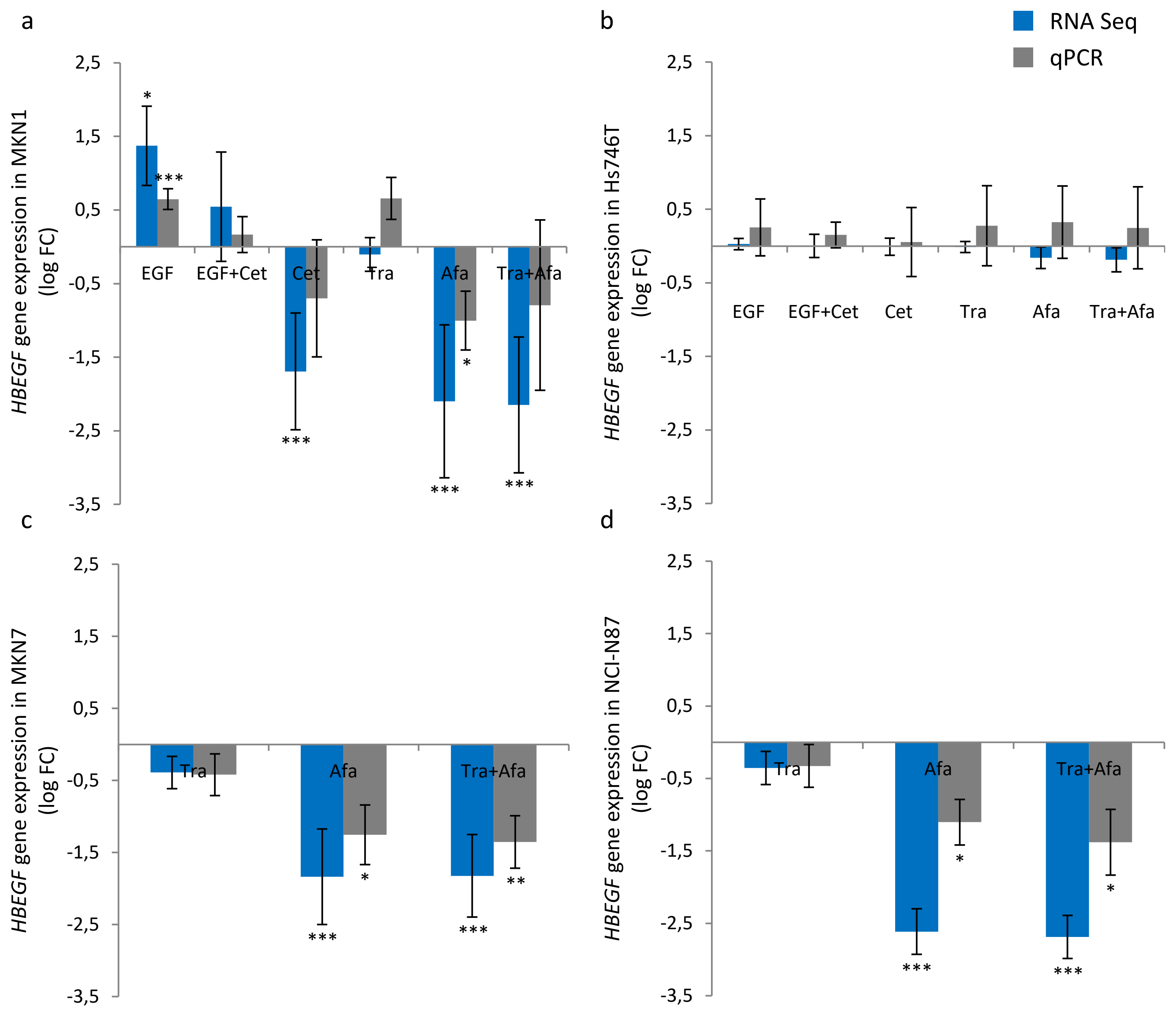

Figure S4: ***HBEGF*** **gene expression measured by RNA Sequencing and qPCR.**
MKN1 (a) and Hs746T (b) cells were treated with EGF, EGF + cetuximab (EGF+Cet), cetuximab (Cet), trastuzumab (Tra), afatinib (Afa) or trastuzumab + afatinib (Tra+Afa) for 24 h. MKN7 (c) and NCI-N87 (d) were treated with trastuzumab (Tra), afatinib (Afa) or trastuzumab + afatinib (Tra+Afa) for 24 h. *HBEGF* gene expression was measured by RNA Sequencing and qPCR. The mean of three biological experiments with standard deviation is shown. Statistically significant effects compared to untreated are indicated by *p<0.05, **p<0.01 or ***p<0.001 (one-sample t-test).

Figure S5

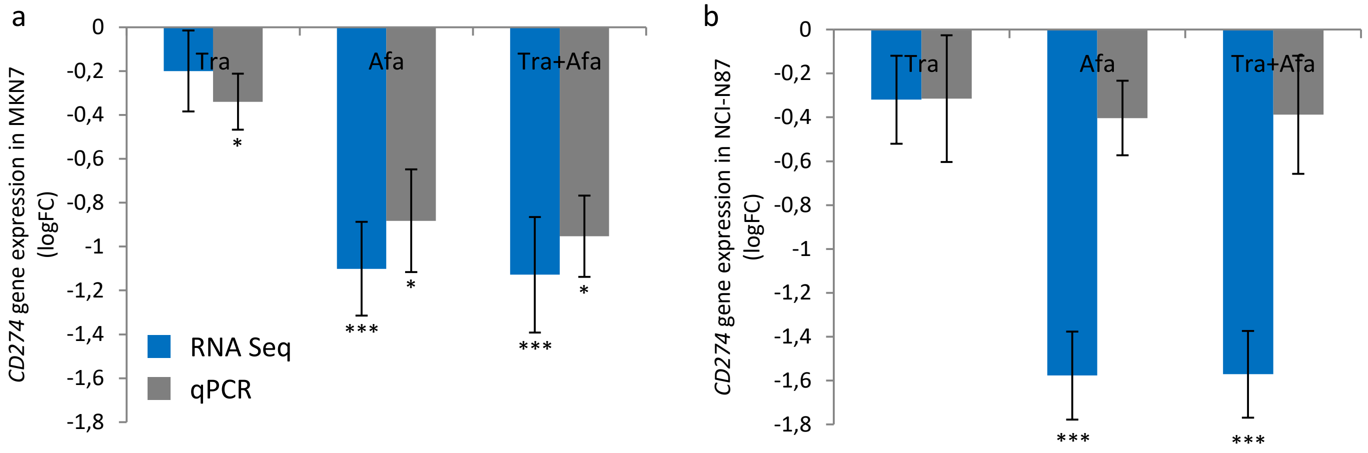

Figure S5: CD274 gene expression measured by RNA Sequencing and qPCR.
MKN7 (a) and NCI-N87 (b) were treated with trastuzumab (Tra), afatinib (Afa) or trastuzumab + afatinib (Tra+Afa) for 24 h. *CD274* gene expression was measured by RNA Sequencing and qPCR. The mean of three biological experiments with standard deviation is shown. Statistically significant effects compared to untreated are indicated by *p<0.05 or ***p<0.001 (one-sample t-test).

Figure S6

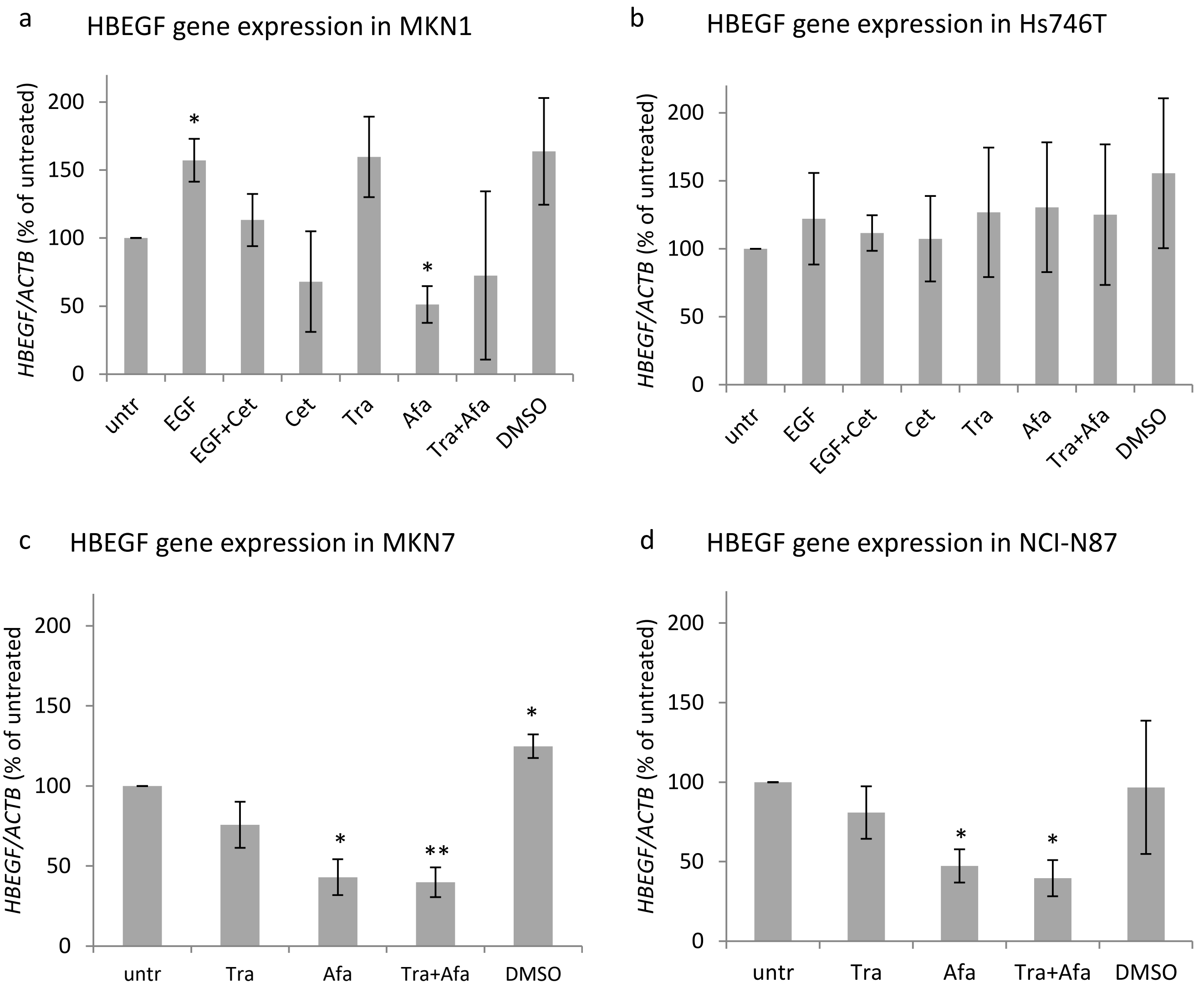

Figure S6: *HBEGF* gene expression measured by qPCR.
MKN1 (a) and Hs746T (b) cells were treated with EGF, EGF + cetuximab (EGF+Cet), cetuximab (Cet), trastuzumab (Tra), afatinib (Afa), trastuzumab + afatinib (Tra+Afa) or DMSO for 24 h. MKN7 (c) and NCI-N87 (d) were treated with trastuzumab (Tra), afatinib (Afa), trastuzumab + afatinib (Tra+Afa) or DMSO for 24 h. *HBEGF* gene expression was measured by qPCR. The mean of three biological experiments with standard deviation is shown. Statistically significant effects compared to untreated are indicated by *p<0.05 or **p<0.01 (one-sample t-test).

Figure S7

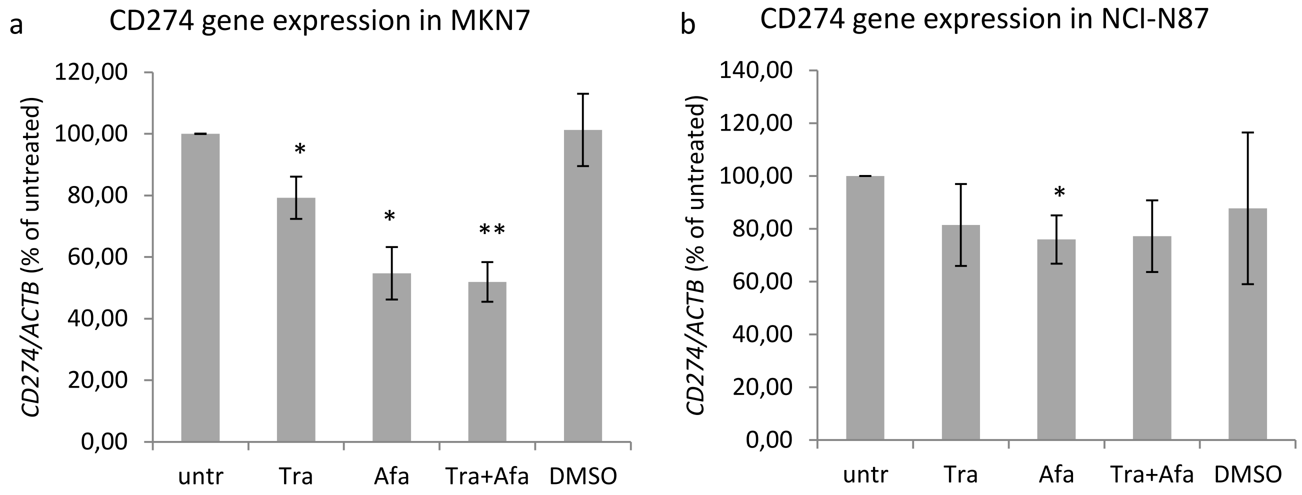

Figure S7: CD274 gene expression measured by qPCR.
MKN7 (a) and NCI-N87 (b) were treated with trastuzumab (Tra), afatinib (Afa), trastuzumab + afatinib (Tra+Afa) or DMSO for 24 h. *CD274* gene expression was measured by qPCR. The mean of three biological experiments with standard deviation is shown. Statistically significant effects compared to untreated are indicated by *p<0.05 or **p<0.01 (one-sample t-test).

Figure S8

a

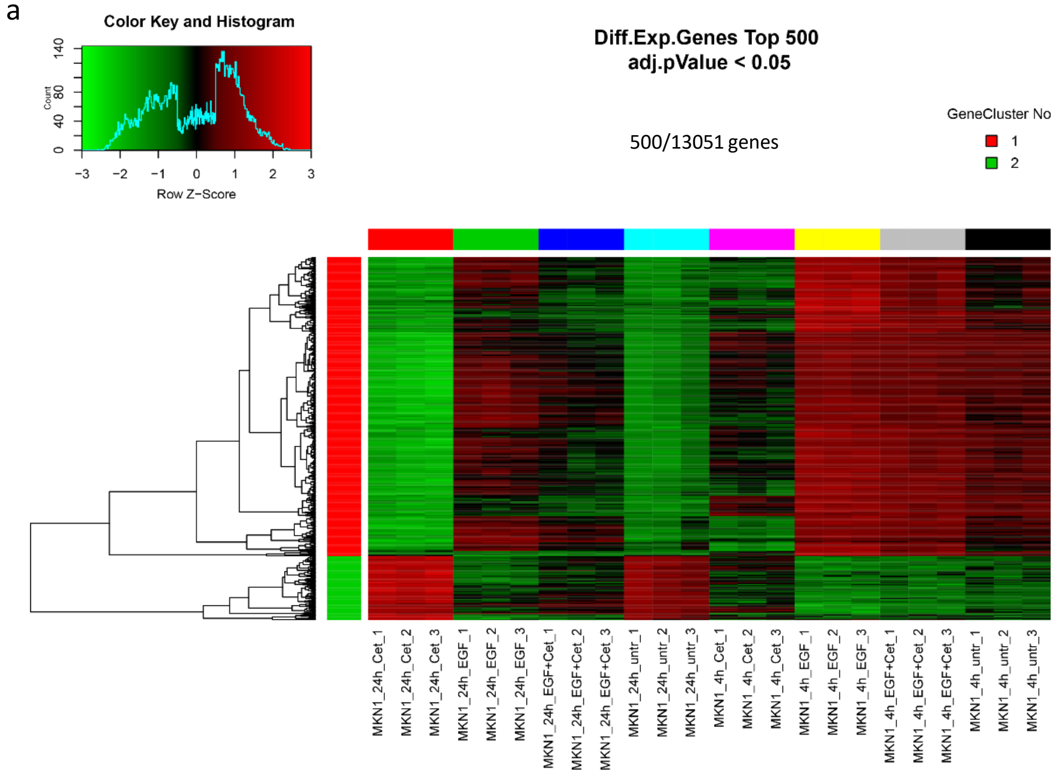

b

Cluster	Description	GeneRatio	p.adjust
1	TNF signaling pathway	17/165	4.24E-07
1	Cellular senescence	17/165	3.75E-05
1	Cytokine-cytokine receptor interaction	19/165	5.44E-05
1	AGE-RAGE signaling pathway in diabetic complications	12/165	0.000571
1	NF-kappa B signaling pathway	11/165	0.000601
1	HTLV-I infection	19/165	0.000679
1	MAPK signaling pathway	20/165	0.00196
1	Rheumatoid arthritis	9/165	0.003243
1	Hippo signaling pathway	13/165	0.00454
1	Transcriptional misregulation in cancer	14/165	0.004674

c

Cluster	Description	GeneRatio	p.adjust
1	Interleukin-10 signaling	8/236	0.00136
1	Interleukin-4 and 13 signaling	12/236	0.001928
1	RAF-independent MAPK1/3 activation	6/236	0.004358
1	SMAD2/SMAD3:SMAD4 heterotrimer regulates transcription	6/236	0.008702
1	Transcriptional activity of SMAD2/SMAD3:SMAD4 heterotrimer	7/236	0.008702
1	RIPK1-mediated regulated necrosis	5/236	0.008702
1	Regulated Necrosis	5/236	0.008702
1	Dissolution of Fibrin Clot	4/236	0.018535
1	Signaling by TGF-beta Receptor Complex	8/236	0.025978
1	Signaling by NOTCH	10/236	0.039259

d

Cluster	Description	GeneRatio	p.adjust
1	endoderm development	13/353	1.6E-05
1	endoderm formation	11/353	1.6E-05
1	leukocyte differentiation	31/353	1.6E-05
1	positive regulation of peptidyl-tyrosine phosphorylation	18/353	1.6E-05
1	kidney development	24/353	1.6E-05
1	kidney epithelium development	17/353	1.71E-05
1	renal system development	24/353	2.91E-05
1	formation of primary germ layer	15/353	2.91E-05
1	regulation of protein serine/threonine kinase activity	32/353	2.91E-05
1	regulation of peptidyl-tyrosine phosphorylation	20/353	4.11E-05

Figure S8: Cluster Profiler analysis of genes regulated by EGF and/or cetuximab in MKN1 cells (Top 500).
MKN1 cells were treated for 4 h or 24 h with cetuximab (Cet), EGF, EGF + cetuximab (EGF+Cet). Untreated cells (untr) were used as control. Three biological experiments were indicated by numbers 1-3. Gene expression was measured by RNA sequencing and differential gene expression was calculated by R package “edgeR”. Illustrated are the Top 500 of 13051 genes with p.adjust <0.05. The expression level is color-coded according to the Color Key (green: low expression, black: medium expression, red: high expression) (a). Functional analysis of Top 500 genes was performed by R package “clusterProfiler” using the KEGG (b), Reactome (c) or GO-term (d) databases. The Top 10 significantly enriched pathways of each cluster with p.adjust <0.05 are depicted.

Figure S9

a

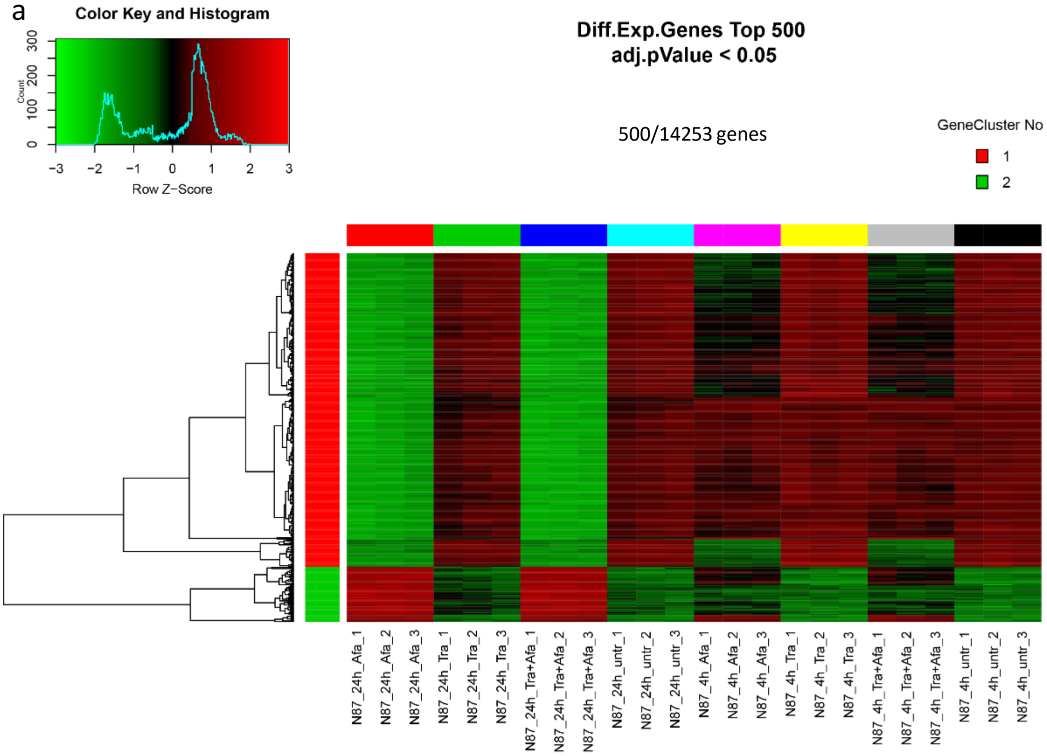

b

Cluster	Description	GeneRatio	p.adjust
1	Cell cycle	25/197	1.37E-11
1	DNA replication	11/197	5.72E-07
1	Pyrimidine metabolism	13/197	0.000561
1	Homologous recombination	8/197	0.000911
1	Purine metabolism	16/197	0.00316
1	MicroRNAs in cancer	14/197	0.017707
1	Cellular senescence	13/197	0.033098
1	Bladder cancer	6/197	0.05132

c

Cluster	Description	GeneRatio	p.adjust
1	Cell Cycle	72/206	7.07E-26
1	Cell Cycle, Mitotic	63/206	7.58E-24
1	Mitotic G1-G1/S phases	26/206	4.31E-12
1	Activation of the pre-replicative complex	14/206	2.03E-11
1	Unwinding of DNA	9/206	6.14E-10
1	Activation of ATR in response to replication stress	14/206	6.14E-10
1	G2/M Checkpoints	16/206	6.14E-10
1	G1/S Transition	21/206	9.22E-10
1	DNA strand elongation	13/206	1.18E-09
1	S Phase	22/206	2.43E-09

d

Cluster	Description	GeneRatio	p.adjust
1	DNA replication	49/377	2.11E-23
1	mitotic cell cycle phase transition	55/377	6.71E-19
1	cell cycle phase transition	56/377	1.1E-18
1	DNA-dependent DNA replication	29/377	4.48E-16
1	G1/S transition of mitotic cell cycle	35/377	8.49E-15
1	DNA replication initiation	17/377	1.24E-14
1	cell cycle G1/S phase transition	35/377	2.32E-14
1	cell cycle checkpoint	27/377	4.67E-09
1	DNA integrity checkpoint	23/377	4.67E-09
1	regulation of cell cycle process	42/377	3.48E-08
2	arachidonic acid secretion	3/49	0.039265
2	arachidonate transport	3/49	0.039265
2	positive regulation of anion transport	3/49	0.039265
2	response to iron ion	3/49	0.039265
2	dopamine metabolic process	3/49	0.039265
2	locomotory behavior	5/49	0.044427
2	icosanoid secretion	3/49	0.044427
2	regulation of neurotransmitter levels	5/49	0.044427
2	response to nicotine	3/49	0.044427
2	response to cocaine	3/49	0.044427

Figure S9: Cluster Profiler analysis of genes regulated by trastuzumab and/or afatinib in NCI-N87 cells (Top 500).
NCI-N87 cells were treated for 4 h or 24 h with trastuzumab (Tra), afatinib (Afa) or trastuzumab + afatinib (Tra+Afa). Untreated cells (untr) were used as control. Three biological experiments were indicated by numbers 1-3. Gene expression was measured by RNA sequencing and differential gene expression was calculated by R package “edgeR”. Illustrated are the Top 500 of 14253 genes with p.adjust <0.05. The expression level is color-coded according to the Color Key (green: low expression, black: medium expression, red: high expression) (a). Functional analysis of Top 500 genes was performed by R package “clusterProfiler” using the KEGG (b), Reactome (c) or GO-term (d) databases. The Top 10 significantly enriched pathways of each cluster with p.adjust <0.05 are depicted.

Figure S10

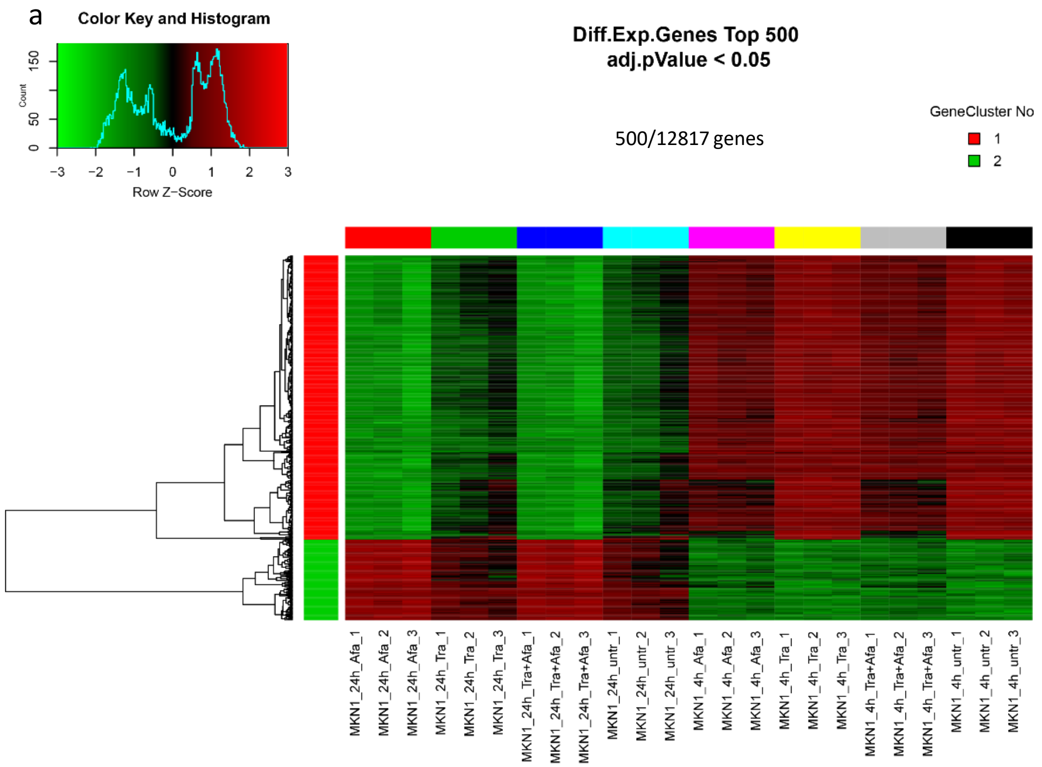

b

Cluster	Description	GeneRatio	p.adjust
1	Ribosome biogenesis in eukaryotes	15/157	4E-08

c

Cluster	Description	GeneRatio	p.adjust
1	rRNA modification in the nucleus and cytosol	14/231	2.65E-08
1	rRNA processing in the nucleus and cytosol	23/231	2.65E-08
1	rRNA processing	23/231	5.17E-08
1	Major pathway of rRNA processing in the nucleolus and cytosol	20/231	1.22E-06
1	tRNA modification in the nucleus and cytosol	7/231	0.010145

d

Cluster	Description	GeneRatio	p.adjust
1	ribosome biogenesis	34/347	1.45E-10
1	ribonucleoprotein complex biogenesis	38/347	5.66E-09
1	ncRNA processing	35/347	5.66E-09
1	rRNA metabolic process	28/347	6.47E-08
1	rRNA processing	25/347	4.23E-07
1	RNA modification	13/347	0.007458
1	heterochromatin assembly	4/347	0.0442
1	maturation of SSU-rRNA	7/347	0.0442
1	maturation of SSU-rRNA from tricistronic rRNA transcript (SSU-rRNA, 5.8S rRNA, LSU-rRNA)	6/347	0.0442
1	ribosomal small subunit biogenesis	8/347	0.0442

Figure S10: Cluster Profiler analysis of genes regulated by trastuzumab and/or afatinib in MKN1 cells (Top 500).
MKN1 cells were treated for 4 h or 24 h with trastuzumab (Tra), afatinib (Afa) or trastuzumab + afatinib (Tra+Afa). Untreated cells (untr) were used as control. Three biological experiments were indicated by numbers 1-3. Gene expression was measured by RNA sequencing and differential gene expression was calculated by R package “edgeR”. Illustrated are the Top 500 of 12817 genes with p.adjust <0.05. The expression level is color-coded according to the Color Key (green: low expression, black: medium expression, red: high expression) (a). Functional analysis of Top 500 genes was performed by R package “clusterProfiler” using the KEGG (b), Reactome (c) or GO-term (d) databases. The Top 10 significantly enriched pathways of each cluster with p.adjust <0.05 are depicted.

Figure S11

a

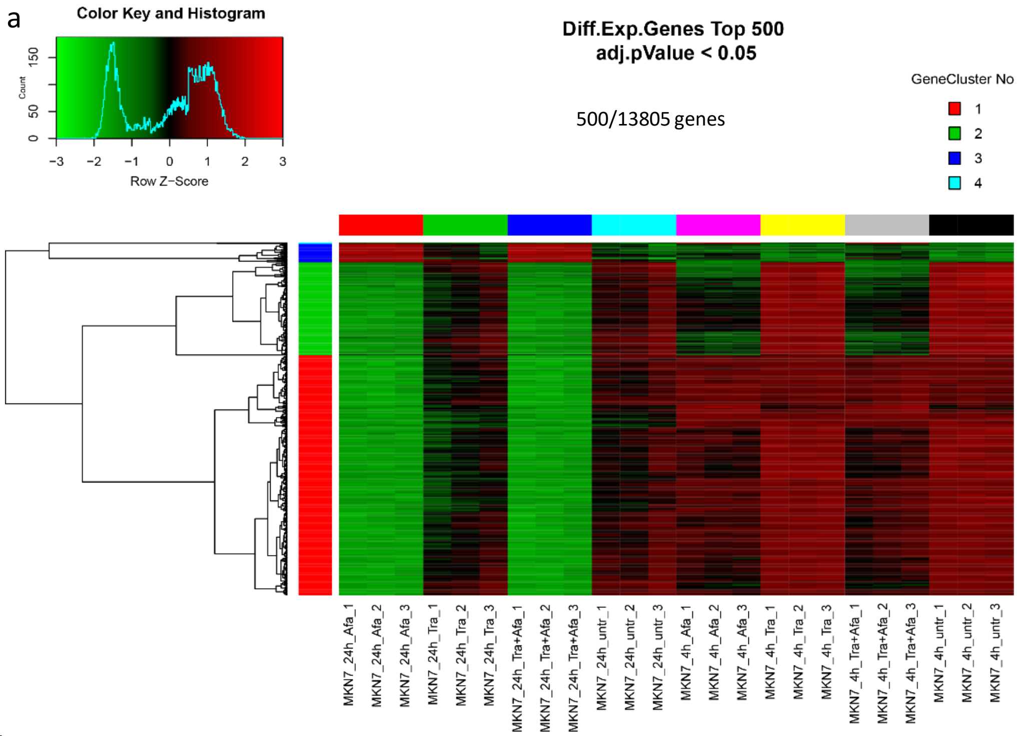

b

Cluster	Description	GeneRatio	p.adjust
1	RNA transport	13/149	0.020616
2	Ribosome biogenesis in eukaryotes	6/47	0.001892
2	Hepatocellular carcinoma	7/47	0.008016
2	Pancreatic cancer	5/47	0.008016
2	Chronic myeloid leukemia	5/47	0.008016
2	HTLV-I infection	7/47	0.053196
3	Phagosome	2/7	0.080888
3	Phenylalanine metabolism	1/7	0.080888
3	Steroid biosynthesis	1/7	0.080888
3	One carbon pool by folate	1/7	0.080888
3	Histidine metabolism	1/7	0.080888
3	Glyoxylate and dicarboxylate metabolism	1/7	0.080888
3	Tyrosine metabolism	1/7	0.080888
3	Nicotinate and nicotinamide metabolism	1/7	0.080888
3	beta-Alanine metabolism	1/7	0.080888
3	Malaria	1/7	0.080888

c

Cluster	Description	GeneRatio	p.adjust
1	Cell Cycle, Mitotic	48/159	2.5E-17
1	M Phase	37/159	2.5E-17
1	Cell Cycle	52/159	4.78E-17
1	Mitotic Prometaphase	23/159	3.01E-14
1	Mitotic Metaphase and Anaphase	28/159	4.32E-14
1	Mitotic Anaphase	27/159	2.8E-13
1	Resolution of Sister Chromatid Cohesion	21/159	5.54E-13
1	Separation of Sister Chromatids	26/159	5.54E-13
1	RHO GTPases Activate Formins	18/159	5.67E-09
1	Kinesins	9/159	1.15E-07
2	Downregulation of TGF-beta receptor signaling	4/40	0.006446
2	TGF-beta receptor signaling activates SMADs	4/40	0.006446
2	Signaling by TGF-beta Receptor Complex	5/40	0.00742
2	Signaling by NOTCH	5/40	0.013927
2	Pre-NOTCH Transcription and Translation	3/40	0.013927
2	Transcriptional activation of mitochondrial biogenesis	3/40	0.013927
2	Pre-NOTCH Expression and Processing	3/40	0.024324
2	Mitochondrial biogenesis	3/40	0.030304
3	O-glycosylation of TSR domain-containing proteins	1/6	0.059147
3	Defective EXT2 causes exostoses 2	1/6	0.059147
3	Defective EXT1 causes exostoses 1, TRPS2 and CHDS	1/6	0.059147
3	Defective B4GALT7 causes EDS, progeroid type	1/6	0.059147
3	Defective B3GAT3 causes JDSSDHD	1/6	0.059147
3	HS-GAG degradation	1/6	0.059147
3	Cholesterol biosynthesis	1/6	0.059147
3	TRP channels	1/6	0.059147
3	Diseases associated with glycosaminoglycan metabolism	1/6	0.059147
3	Diseases of glycosylation	1/6	0.059147
4	HATs acetylate histones	1/1	0.036627
4	Chromatin modifying enzymes	1/1	0.036627
4	Chromatin organization	1/1	0.036627

d

Cluster	Description	GeneRatio	p.adjust
1	sister chromatid segregation	35/316	4.2E-18
1	chromosome segregation	41/316	9.57E-18
1	nuclear chromosome segregation	37/316	4.69E-17
1	mitotic nuclear division	46/316	4.69E-17
1	sister chromatid cohesion	23/316	8.54E-13
1	mitotic sister chromatid segregation	23/316	2.77E-12
1	mitotic spindle organization	13/316	9.37E-09
1	microtubule cytoskeleton organization	33/316	2.83E-08
1	regulation of cell cycle process	38/316	2.86E-08
1	spindle organization	16/316	1.47E-07
2	ncRNA processing	13/118	0.026724
2	inactivation of MAPK activity	4/118	0.02869
2	negative regulation of MAPK cascade	7/118	0.047385
2	negative regulation of transforming growth factor beta receptor signaling pathway	5/118	0.047385
2	negative regulation of cellular response to transforming growth factor beta stimulus	5/118	0.047385
2	ribosome biogenesis	10/118	0.047385
2	rRNA processing	9/118	0.047385
2	rRNA metabolic process	9/118	0.049651
2	ribonucleoprotein complex biogenesis	12/118	0.049651
2	regulation of protein serine/threonine kinase activity	12/118	0.049651
4	histone H2A acetylation	1/2	0.032144
4	regulation of glutamate receptor signaling pathway	1/2	0.032144
4	regulation of neurotransmitter receptor activity	1/2	0.032144
4	histone H4 acetylation	1/2	0.038832
4	glutamate receptor signaling pathway	1/2	0.038832
4	regulation of receptor activity	1/2	0.038832
4	histone acetylation	1/2	0.038832
4	internal peptidyl-lysine acetylation	1/2	0.038832
4	peptidyl-lysine acetylation	1/2	0.038832
4	internal protein amino acid acetylation	1/2	0.038832

Figure S11: Cluster Profiler analysis of genes regulated by trastuzumab and/or afatinib in MKN7 cells (Top 500).
MKN7 cells were treated for 4 h or 24 h with trastuzumab (Tra), afatinib (Afa) or trastuzumab + afatinib (Tra+Afa). Untreated cells (untr) were used as control. Three biological experiments were indicated by numbers 1-3. Gene expression was measured by RNA sequencing and differential gene expression was calculated by R package “edgeR”. Illustrated are the Top 500 of 12817 genes with p.adjust <0.05. The expression level is color-coded according to the Color Key (green: low expression, black: medium expression, red: high expression) (a). Functional analysis of Top 500 genes was performed by R package “clusterProfiler” using the KEGG (b), Reactome (c) or GO-term (d) databases. The Top 10 significantly enriched pathways of each cluster with p.adjust <0.05 are depicted.

Figure S12

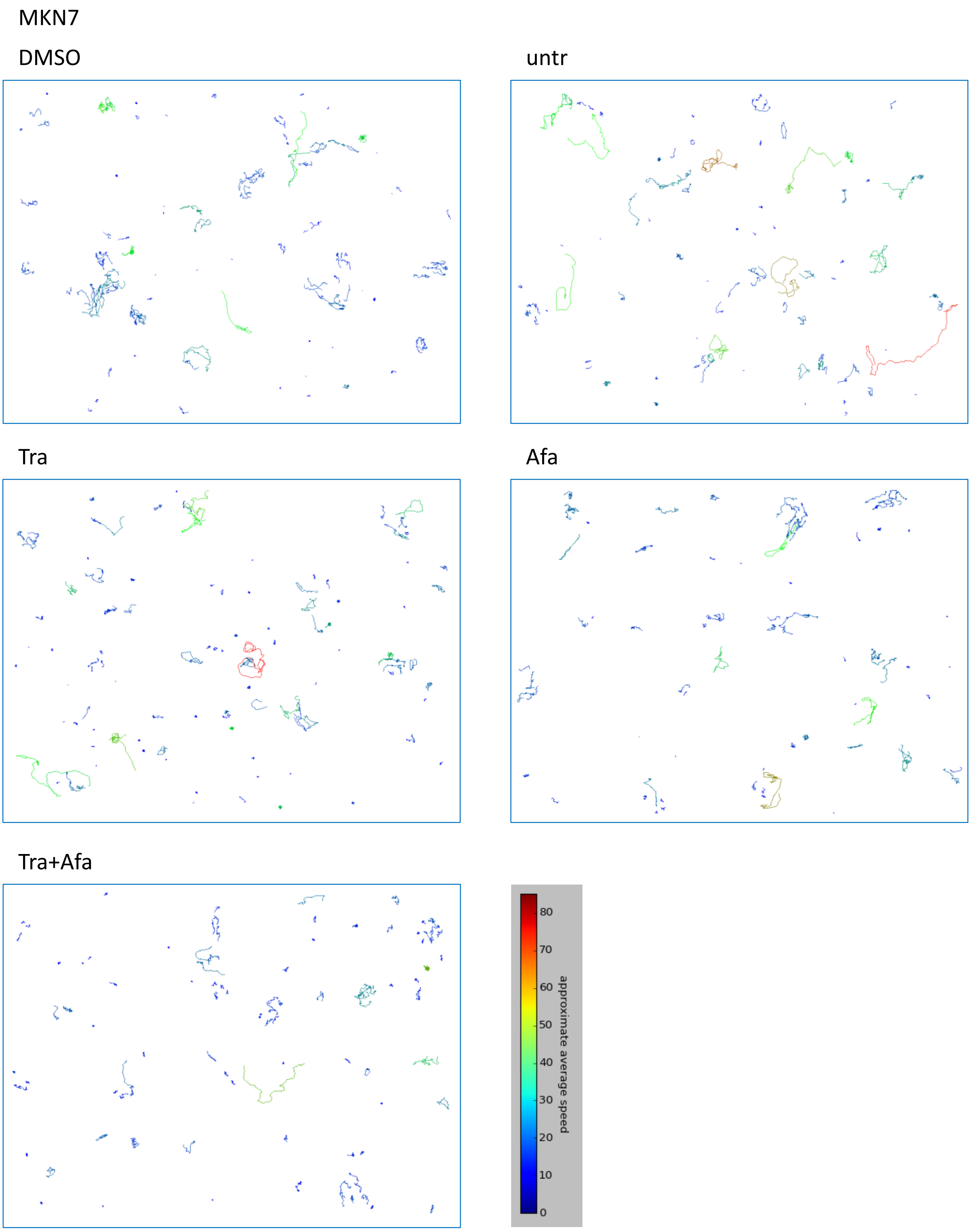

Figure S12: Trajectories of MKN7 cells treated with trastuzumab or afatinib.
MKN7 cells were treated with 5 µg/ml trastuzumab (Tra), 0.5 µM afatinib (Afa), 5 µg/ml trastuzumab + 0.5 µM afatinib (Tra+Afa) or afatinib solvent DMSO (0.05%). Untreated (untr) cells were used as control. Cell movement was tracked for 7 hours to assess approximate average speed. The trajectories of one exemplary film for each condition are shown. The trajectories were color-coded for approximate average speed.

Figure S13

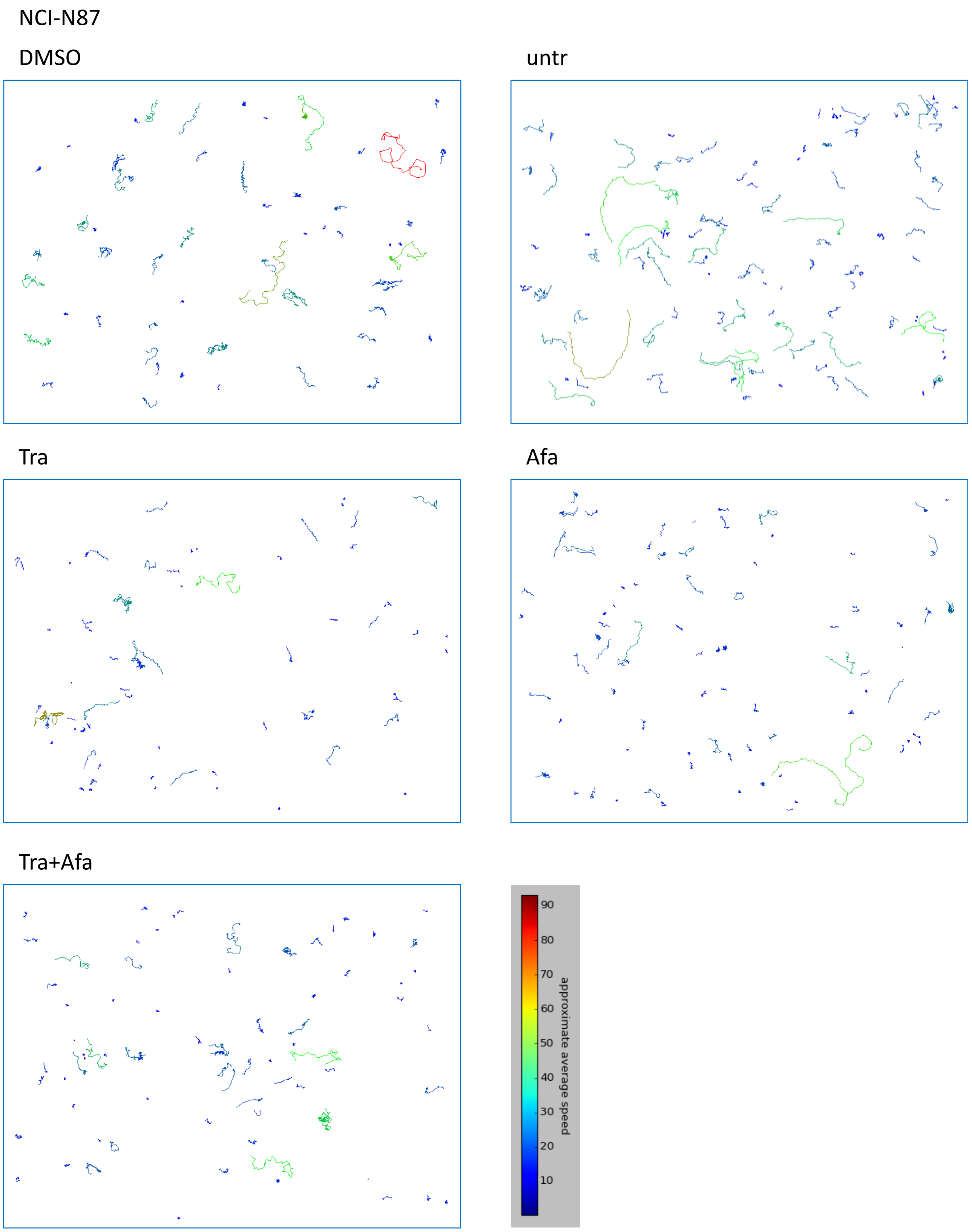

Figure S13: Trajectories of NCI-N87 cells treated with trastuzumab or afatinib.
NCI-N87 cells were treated with 5 µg/ml trastuzumab (Tra), 0.5 µM afatinib (Afa), 5 µg/ml trastuzumab + 0.5 µM afatinib (Tra+Afa) or afatinib solvent DMSO (0.05%). Untreated (untr) cells were used as control. Cell movement was tracked for 7 hours to assess approximate average speed. The trajectories of one exemplary film for each condition are shown. The trajectories were color-coded for approximate average speed.

Figure S14

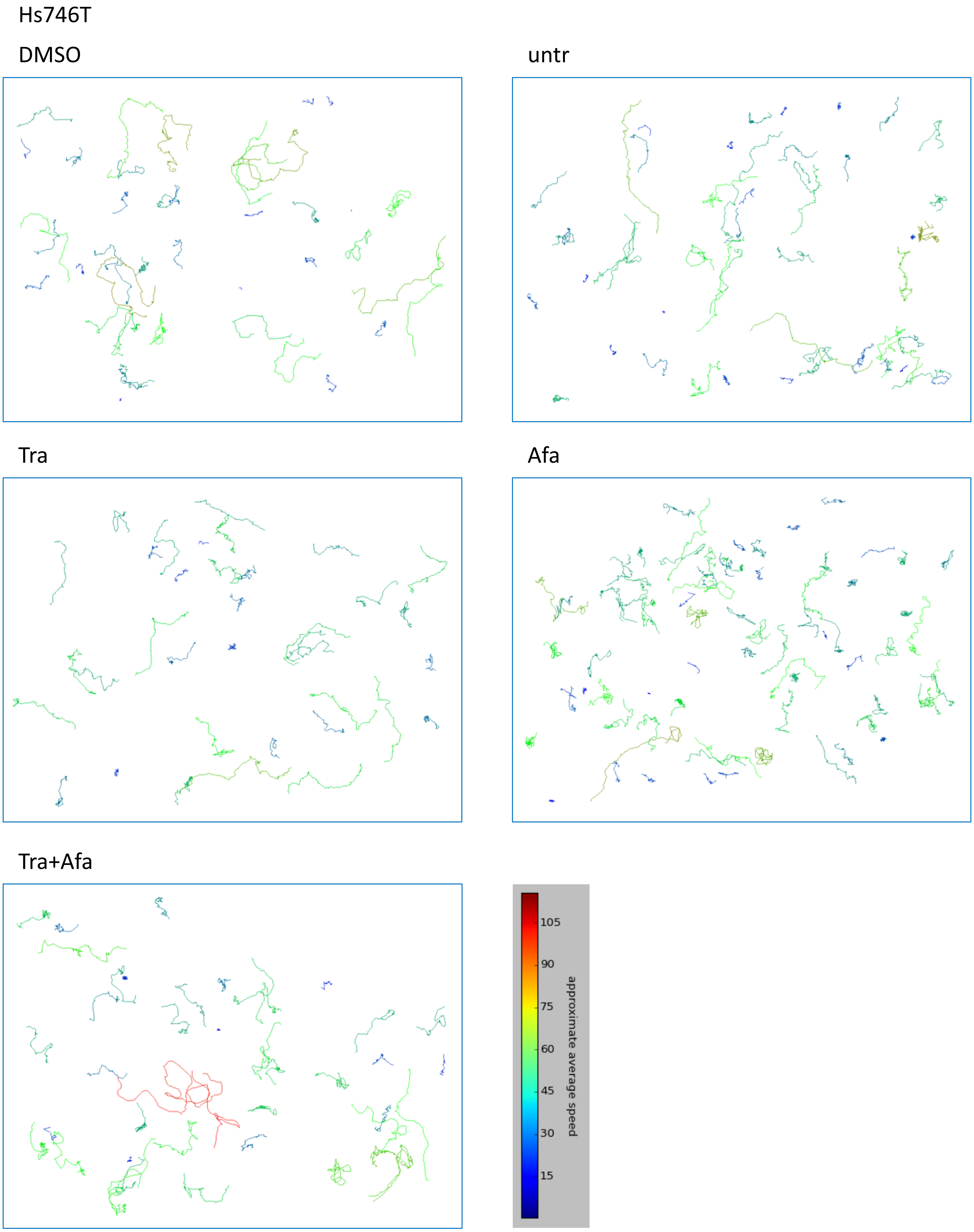

Figure S14: Trajectories of Hs746T cells treated with trastuzumab or afatinib.

Hs746T cells were treated with 5 µg/ml trastuzumab (Tra), 0.5 µM afatinib (Afa), 5 µg/ml trastuzumab + 0.5 µM afatinib (Tra+Afa) or afatinib solvent DMSO (0.05%). Untreated (untr) cells were used as control. Cell movement was tracked for 7 hours to assess approximate average speed. The trajectories of one exemplary film for each condition are shown. The trajectories were color-coded for approximate average speed.
