# Supplementary material for: Determining the effects of trastuzumab, cetuximab and afatinib by phosphoprotein, gene expression and phenotypic analysis in gastric cancer cell lines
Source: BMC Cancer. 2020 Oct 28;20:1039. doi: 10.1186/s12885-020-07540-7 (PMC7594334; doi:10.1186/s12885-020-07540-7)
Supplement: Supplementary file 3 — Additional file 3. Supplemental Results. [file 12885_2020_7540_MOESM3_ESM.pdf]

Supplemental Results

Confirmation of regulated genes by qPCR including DMSO as solvent control

Two selected genes were validated by qPCR. The gene expression levels of *HBEGF* and *CD274 (PD-L1)* were qualitatively confirmed. The Pearson correlation coefficient ranged from 0.9008 to 0.9969 whilst the adjusted p-value ranged from 0.0152 to 0.0699 in NCI-N87, MKN1 and MKN7 cells. Due to the absence of any treatment effects, no correlations were observed in Hs746T cells **(Additional file 2, Figures S4 and S5 and Additional file 4, Table S1)**. DMSO as afatinib solvent was included in the validation experiments. Except for minor effects of DMSO on *HBEGF* expression in MKN7 cells, we observed no changes in gene expression following DMSO treatment. Of note, the mentioned effects of DMSO were in opposite direction than that of afatinib. Consequently, the effects we observed after afatinib treatment are caused by afatinib itself and not by its solvent DMSO **(Additional file 2, Figures S6 and S7)**.

Cetuximab – Cluster Profiler analysis

Cluster Profiler analysis of the top 500 genes with p.adjust <0.05 in MKN1 cells after cetuximab and/or EGF treatment resulted in two clusters. Cluster 1 (red) was downregulated after 4 h cetuximab treatment, cluster 2 (green) was upregulated. EGF caused an opposite reaction (**Figure S8 a, Additional file 2**). Functional enrichment analysis revealed significantly enriched pathways in cluster 1. Enriched KEGG pathways in cluster 1 included for example TNF signaling pathway, Cytokine cytokine receptor interaction, NF-kappa B signaling pathway, MAPK signaling pathway and Transcriptional misregulation in cancer **(Figure S8 b, Additional file 2)**. Using Reactome pathways, these were amongst others signaling pathways such as Interleukin-10, Interleukin-4 and interleukin-13 signaling, RAF-independent MAPK1/3 activation, Signaling by TGF-beta Receptor Complex and Signaling by Notch (**Figure S8 c, Additional file 2**). With regard to the GO terms, cluster 1 included e.g. developmental processes (endoderm development, kidney development) and the GO-terms regulation of protein serine/theronine kinase activity and regulation of peptidyl-tyrosine phosphorylation (**Figure S8 d, Additional file 2**). Listed are the top 10 significantly enriched pathways for each cluster.

Trastuzumab and afatinib – Cluster Profiler analysis

The Cluster Profiler analysis of the top 500 genes with p.adjust <0.05 in NCI-N87 cells resulted in 2 clusters. Cluster 1 was downregulated and cluster 2 was upregulated by afatinib after 24 h. After 4 hours of afatinib treatment, only part of cluster 1 was downregulated and only part of cluster 2 was

upregulated. Since more genes were regulated following 24 h than following 4 h treatment, the results are mainly driven by the 24 h timepoint. (**Figure S9 a, Additional file 2**). The KEGG pathways in cluster 1 affected cell cycle processes (e.g. Cell Cycle, DNA replication) as well as the nucleobase metabolism (Pyrimidine metabolism, Purine metabolism), and the pathway Micro-RNAs in cancer (**Figure S9 b, Additional file 2**). Cluster 1 contained Reactome pathways that are involved in the cell cycle (e.g. Cell Cycle, Mitotic G1-G1/S phases, DNA strand elongation) (**Figure S9 c, Additional file 2**). The biological functions (GO terms) enriched in cluster 1 also have a function in the cell cycle (e.g. DNA replication, G1/S transition of mitotic cell cyle, cell cycle checkpoint). In cluster 2, various processes such as arachidonate transport, positive regulation of anion transport, locomotive behavior and icosanoid secretion were enriched (**Figure S9 d, Additional file 2**). Listed are the top 10 significantly enriched pathways for each cluster.

The Cluster Profiler analysis of top 500 genes with p.adjust <0.05 identified after trastuzumab/afatinib treatment in MKN1 cells resulted in 2 clusters. Cluster 1 was downregulated by afatinib after 4 h and 24 h, cluster 2 was upregulated (**Figure S10 a, Additional file 2**). The KEGG pathway Ribosome biogenesis in eukaryotes was enriched in cluster 1 (**Figure S10 b, Additional file 2**). Reactome pathways enriched in cluster 1 were RNA pathways (e.g. rRNA modification in the nucleus and cytosol, rRNA processing, tRNA modification in the nucleus and cytosol) (**Figure S10 c, Additional file 2**). Biological functions (GO terms) in cluster 1 are also involved in RNA processes (ribosome biogenesis, rRNA processing, RNA modification). Afatinib thus seems to inhibit the function of ribosomes and therefore protein synthesis (**Figure S10 d, Additional file 2**). Listed are the top 10 significantly enriched pathways for each cluster.

The Cluster Profiler analysis of top 500 genes with p.adjust <0.05 identified after trastuzumab/afatinib treatment in MKN7 cells resulted in 4 clusters. Cluster 1 was strongly downregulated by afatinib after 24 h and slightly downregulated after 4 h. Cluster 2 was downregulated after 4 h and 24 h. Thus, cluster 2 contained genes that mediate a rapid response to afatinib. Cluster 3 was strongly upregulated by afatinib after 24 h and slightly upregulated after 4 h (**Figure S11 a, Additional file 2**). The KEGG pathway analysis revealed one enriched pathway in cluster 1 (RNA transport) and various pathways in cluster 2 (e.g. Ribosome biogenesis in eukaryotes, Hepatocellular carcinoma, Pancreatic cancer). Cluster 3 included metabolic pathways (e.g. Phenylalanine metabolism, Histidine metabolism, Tyrosine metabolism, Steroid biosynthesis) (**Figure S11 b, Additional file 2**). Cell cycle pathways (e.g. Cell Cycle, M Phase, Mitotic Anaphase) were overrepresented in cluster 1 (Reactome). Cluster 2 contained genes involved in signaling pathways (e.g. Downregulation of TGF-beta receptor signaling, Signaling by NOTCH). Cluster 3 contained glycosylation-related pathways (HS-GAG degradation, Diseases associated with glycosaminoglycan metabolism, Diseases of glycosylation, O-glycosylation of TSR domain-containing proteins) and the

pathways Cholesterol biosynthesis and TRP channels (**Figure S11 c, Additional file 2**). Biological functions enriched in cluster 1 were cell cycle processes (e.g. mitotic nuclear division, regulation of cell cycle process, spindle organization) and RNA processes (ribosomal biogenesis, rRNA processing, rRNA metabolic process). The mentioned RNA processes were also enriched in cluster 2. In addition, signaling pathways were also overrepresented in cluster 2 (e.g. inactivation of MAPK activity, negative regulation of transforming growth factor beta receptor signaling, regulation of protein serine/threonine kinase activity) (**Figure S11 d, Additional file 2**). Listed are the top 10 significantly enriched pathways for each cluster.
