# Supplementary material for: Determining the effects of trastuzumab, cetuximab and afatinib by phosphoprotein, gene expression and phenotypic analysis in gastric cancer cell lines
Source: BMC Cancer. 2020 Oct 28;20:1039. doi: 10.1186/s12885-020-07540-7 (PMC7594334; doi:10.1186/s12885-020-07540-7)
Supplement: Supplementary file 4 — Additional file 4. Supplemental Tables. [file 12885_2020_7540_MOESM4_ESM.pdf]

Table S1: Correlation of gene expression measured by RNA sequencing and qPCR.

RNA Seq /qPCR			
**Gene**	**Cell line**	**Pearson**	**p adjusted**
HBEGF	MKN1	0.9008	0.0152
HBEGF	MKN7	0.9936	0.0509
HBEGF	Hs746T	-0.4851	0.8875
HBEGF	NCI-N87	0.9859	0.0699
CD274	NCI-N87	0.9969	0.0472
CD274	MKN7	0.9955	0.0509

Table S2: Significantly enriched KEGG pathways in MKN1 cells after 4 h cetuximab treatment.

ID	Description	GeneRatio	BgRatio	p.adjust
hsa05323	Rheumatoid arthritis	6/43	70/5844	0.00120571
hsa04657	IL-17 signaling pathway	6/43	79/5844	0.00121677
hsa04060	Cytokine-cytokine receptor interaction	8/43	186/5844	0.00198674
hsa04668	TNF signaling pathway	6/43	102/5844	0.00259938
hsa04010	MAPK signaling pathway	8/43	272/5844	0.01366521
hsa04062	Chemokine signaling pathway	6/43	145/5844	0.01366521
hsa05202	Transcriptional misregulation in cancer	6/43	168/5844	0.02194309
hsa05161	Hepatitis B	5/43	125/5844	0.0277262
hsa05132	Salmonella infection	4/43	75/5844	0.0277262
hsa05224	Breast cancer	5/43	139/5844	0.03890197
hsa04660	T cell receptor signaling pathway	4/43	89/5844	0.04230156
hsa05167	Kaposi's sarcoma-associated herpesvirus infection	5/43	155/5844	0.04767251
hsa04933	AGE-RAGE signaling pathway in diabetic complications	4/43	95/5844	0.04767251

Table S3: Significantly enriched KEGG pathways in MKN1 cells after 24 h cetuximab treatment.

ID	Description	GeneRatio	BgRatio	p.adjust
hsa05202	Transcriptional misregulation in cancer	10/72	168/5844	0.00617194

Table S4: Significantly enriched Reactome pathways in MKN1 cells after 4 h cetuximab treatment.

ID	Description	GeneRatio	BgRatio	p.adjust
380108	Chemokine receptors bind chemokines	5/39	31/5594	0.00039879
375276	Peptide ligand-binding receptors	6/39	119/5594	0.01591128

Table S5: Significantly enriched KEGG pathways in MKN1 cells after 4 h EGF treatment.

ID	Description	GeneRatio	BgRatio	p.adjust
hsa04657	IL-17 signaling pathway	11/103	79/5844	1.7557E-05
hsa04060	Cytokine-cytokine receptor interaction	15/103	186/5844	6.1425E-05
hsa04668	TNF signaling pathway	11/103	102/5844	8.2434E-05
hsa05323	Rheumatoid arthritis	8/103	70/5844	0.0011995
hsa05205	Proteoglycans in cancer	12/103	186/5844	0.00325511
hsa04621	NOD-like receptor signaling pathway	10/103	140/5844	0.00469904
hsa04390	Hippo signaling pathway	10/103	146/5844	0.00569143
hsa05132	Salmonella infection	7/103	75/5844	0.00707786
hsa05200	Pathways in cancer	19/103	469/5844	0.00883296
hsa05217	Basal cell carcinoma	6/103	58/5844	0.00883296
hsa05219	Bladder cancer	5/103	39/5844	0.00887918
hsa04012	ErbB signaling pathway	7/103	84/5844	0.00946041
hsa04062	Chemokine signaling pathway	9/103	145/5844	0.0130742
hsa04933	AGE-RAGE signaling pathway in diabetic complications	7/103	95/5844	0.01689209
hsa05161	Hepatitis B	8/103	125/5844	0.0179504
hsa05224	Breast cancer	8/103	139/5844	0.03198886
hsa05146	Amoebiasis	6/103	82/5844	0.03198886
hsa01522	Endocrine resistance	6/103	90/5844	0.04798049

Table S6: Significantly enriched KEGG pathways in MKN1 cells after 24 h EGF treatment.

ID	Description	GeneRatio	BgRatio	p.adjust
hsa04060	Cytokine-cytokine receptor interaction	31/389	186/5844	0.00038465
hsa05202	Transcriptional misregulation in cancer	26/389	168/5844	0.00537984
hsa04630	Jak-STAT signaling pathway	20/389	125/5844	0.01567177
hsa04974	Protein digestion and absorption	14/389	72/5844	0.01567177
hsa04510	Focal adhesion	26/389	190/5844	0.01718496
hsa04151	PI3K-Akt signaling pathway	35/389	296/5844	0.02233751
hsa04610	Complement and coagulation cascades	12/389	61/5844	0.02233751
hsa04512	ECM-receptor interaction	13/389	74/5844	0.03600776
hsa05205	Proteoglycans in cancer	24/389	186/5844	0.03787779

Table S7: Significantly enriched Reactome pathways in MKN1 cells after 4 h EGF treatment.

ID	Description	GeneRatio	BgRatio	p.adjust
380108	Chemokine receptors bind chemokines	7/98	31/5594	0.00027319
375276	Peptide ligand-binding receptors	9/98	119/5594	0.03119512
372790	Signaling by GPCR	19/98	453/5594	0.03119512
500792	GPCR ligand binding	13/98	253/5594	0.03754235

Table S8: Significantly enriched Reactome pathways in MKN1 cells after 24 h EGF treatment.

ID	Description	GeneRatio	BgRatio	p.adjust
373076	Class A/1 (Rhodopsin-like receptors)	39/379	202/5594	7.9336E-07
500792	GPCR ligand binding	44/379	253/5594	1.0274E-06
375276	Peptide ligand-binding receptors	26/379	119/5594	1.3179E-05
1474228	Degradation of the extracellular matrix	21/379	99/5594	0.00032029
1442490	Collagen degradation	15/379	57/5594	0.00046803
372790	Signaling by GPCR	56/379	453/5594	0.00048009
3000178	ECM proteoglycans	15/379	59/5594	0.00048009
2022090	Assembly of collagen fibrils and other multimeric structures	12/379	39/5594	0.00048009
388396	GPCR downstream signaling	48/379	387/5594	0.00157328
1474244	Extracellular matrix organization	32/379	227/5594	0.00322589
380108	Chemokine receptors bind chemokines	9/379	31/5594	0.00876351
446107	Type I hemidesmosome assembly	5/379	10/5594	0.01465555
879518	Transport of organic anions	5/379	11/5594	0.0230509
216083	Integrin cell surface interactions	14/379	76/5594	0.0230509
1474290	Collagen formation	13/379	69/5594	0.0271921
418594	G alpha (i) signalling events	18/379	116/5594	0.02980547
140875	Common Pathway of Fibrin Clot Formation	6/379	19/5594	0.04380188
2022870	Chondroitin sulfate biosynthesis	6/379	19/5594	0.04380188

Table S9: Significantly enriched KEGG pathways in NCI-N87 cells after 4 h afatinib treatment.

**ID**	**Description**	**GeneRatio**	**BgRatio**	**p.adjust**
**hsa04668**	TNF signaling pathway	11/147	102/5844	0.00964085
**hsa05132**	Salmonella infection	9/147	75/5844	0.01059343
**hsa04060**	Cytokine-cytokine receptor interaction	14/147	186/5844	0.01599995

Table S10: Significantly enriched KEGG pathways in NCI-N87 cells after 24 h afatinib treatment.

**ID**	**Description**	**GeneRatio**	**BgRatio**	**p.adjust**
**hsa03030**	DNA replication	32/1970	33/5844	4.4939E-12
**hsa03008**	Ribosome biogenesis in eukaryotes	51/1970	69/5844	1.2039E-09
**hsa04110**	Cell cycle	75/1970	118/5844	2.2175E-09
**hsa00240**	Pyrimidine metabolism	51/1970	89/5844	0.00022402
**hsa03430**	Mismatch repair	17/1970	20/5844	0.00022402
**hsa03013**	RNA transport	76/1970	150/5844	0.00054916
**hsa03050**	Proteasome	26/1970	40/5844	0.00215398
**hsa03440**	Homologous recombination	23/1970	35/5844	0.00402141
**hsa00230**	Purine metabolism	75/1970	157/5844	0.00524042
**hsa03460**	Fanconi anemia pathway	28/1970	47/5844	0.00659236
**hsa03410**	Base excision repair	20/1970	30/5844	0.00659236
**hsa03020**	RNA polymerase	17/1970	26/5844	0.02392305
**hsa03040**	Spliceosome	57/1970	120/5844	0.02583331

Table S11: Significantly enriched Reactome pathways in NCI-N87 cells after 24 h afatinib treatment.

ID	Description	GeneRatio	BgRatio	p.adjust
**1640170**	Cell Cycle	262/1891	462/5594	4.5934E-23
**69278**	Cell Cycle, Mitotic	221/1891	380/5594	3.1704E-21
**453279**	Mitotic G1-G1/S phases	81/1891	114/5594	8.6401E-14
**69242**	S Phase	78/1891	110/5594	2.8328E-13
**69306**	DNA Replication	68/1891	92/5594	5.238E-13
**69206**	G1/S Transition	68/1891	94/5594	2.6703E-12
**69239**	Synthesis of DNA	64/1891	88/5594	9.1325E-12
**2500257**	Resolution of Sister Chromatid Cohesion	62/1891	85/5594	1.4512E-11
**2555396**	Mitotic Metaphase and Anaphase	94/1891	149/5594	1.4512E-11
**68877**	Mitotic Prometaphase	65/1891	91/5594	1.508E-11
**2467813**	Separation of Sister Chromatids	90/1891	141/5594	1.508E-11
**5663220**	RHO GTPases Activate Formins	67/1891	95/5594	1.5703E-11
**68882**	Mitotic Anaphase	93/1891	148/5594	1.9281E-11
**68886**	M Phase	123/1891	216/5594	7.1954E-11
**69190**	DNA strand elongation	29/1891	31/5594	2.6593E-10
**69620**	Cell Cycle Checkpoints	75/1891	116/5594	4.376E-10
**69481**	G2/M Checkpoints	39/1891	49/5594	3.0847E-09
**68874**	M/G1 Transition	51/1891	72/5594	6.2252E-09
**69002**	DNA Replication Pre-Initiation	51/1891	72/5594	6.2252E-09
**68962**	Activation of the pre-replicative complex	25/1891	28/5594	7.605E-08
**180786**	Extension of Telomeres	22/1891	24/5594	2.2875E-07
**73886**	Chromosome Maintenance	47/1891	69/5594	2.2875E-07
**113510**	E2F mediated regulation of DNA replication	24/1891	28/5594	8.3859E-07
**176187**	Activation of ATR in response to replication stress	28/1891	35/5594	1.018E-06
**162909**	Host Interactions of HIV factors	69/1891	118/5594	1.018E-06
**174417**	Telomere C-strand (Lagging Strand) Synthesis	20/1891	22/5594	1.4433E-06
**69300**	Removal of licensing factors from origins	42/1891	64/5594	5.8719E-06
**69304**	Regulation of DNA replication	42/1891	64/5594	5.8719E-06
**195258**	RHO GTPase Effectors	108/1891	213/5594	5.8719E-06
**69186**	Lagging Strand Synthesis	18/1891	20/5594	9.1619E-06
**68867**	Assembly of the pre-replicative complex	39/1891	59/5594	1.1432E-05
**68949**	Orc1 removal from chromatin	40/1891	62/5594	2.0015E-05
**69052**	Switching of origins to a post-replicative state	40/1891	62/5594	2.0015E-05
**174411**	Polymerase switching on the C-strand of the telomere	13/1891	13/5594	2.0098E-05
**69091**	Polymerase switching	13/1891	13/5594	2.0098E-05
**69109**	Leading Strand Synthesis	13/1891	13/5594	2.0098E-05
**177243**	Interactions of Rev with host cellular proteins	24/1891	32/5594	5.7049E-05
**176974**	Unwinding of DNA	11/1891	11/5594	0.00017
**109977**	Repair synthesis for gap-filling by DNA polymerase in TC-NER	13/1891	14/5594	0.00017
**74967**	Repair synthesis of patch ~27-30 bases long by DNA polymerase	13/1891	14/5594	0.00017
**73894**	DNA Repair	70/1891	134/5594	0.00017
**165054**	Rev-mediated nuclear export of HIV RNA	22/1891	30/5594	0.00024

**180746**	Nuclear import of Rev protein	22/1891	30/5594	0.00024
**72203**	Processing of Capped Intron-Containing Pre-mRNA	60/1891	112/5594	0.00024
**162906**	HIV Infection	97/1891	201/5594	0.00024
**194315**	Signaling by Rho GTPases	143/1891	317/5594	0.00024
**191859**	snRNP Assembly	17/1891	21/5594	0.00025
**194441**	Metabolism of non-coding RNA	17/1891	21/5594	0.00025
**109979**	Gap-filling DNA repair synthesis and ligation in TC-NER	14/1891	16/5594	0.00028
**74969**	Gap-filling DNA repair synthesis and ligation in GG-NER	14/1891	16/5594	0.00028
**69202**	Cyclin E associated events during G1/S transition	34/1891	55/5594	0.00035
**69166**	Removal of the Flap Intermediate	12/1891	13/5594	0.00036
**69205**	G1/S-Specific Transcription	12/1891	13/5594	0.00036
**68827**	CDT1 association with the CDC6:ORC:origin complex	33/1891	53/5594	0.00036
**390466**	Chaperonin-mediated protein folding	20/1891	27/5594	0.00039
**72163**	mRNA Splicing - Major Pathway	57/1891	108/5594	0.00056
**72172**	mRNA Splicing	57/1891	108/5594	0.00056
**69183**	Processive synthesis on the lagging strand	13/1891	15/5594	0.00062
**174143**	APC/C-mediated degradation of cell cycle proteins	42/1891	74/5594	0.00063
**453276**	Regulation of mitotic cell cycle	42/1891	74/5594	0.00063
**389957**	Prefoldin mediated transfer of substrate to CCT/TriC	19/1891	26/5594	0.00076
**389958**	Cooperation of Prefoldin and TriC/CCT in actin and tubulin folding	19/1891	26/5594	0.00076
**157579**	Telomere Maintenance	27/1891	42/5594	0.00078
**72165**	mRNA Splicing - Minor Pathway	25/1891	38/5594	0.00082
**5357801**	Programmed Cell Death	70/1891	141/5594	0.00093
**389960**	Formation of tubulin folding intermediates by CCT/TriC	15/1891	19/5594	0.00106
**210991**	Basigin interactions	17/1891	23/5594	0.00140
**379716**	Cytosolic tRNA aminoacylation	17/1891	23/5594	0.00140
**189200**	Hexose transport	25/1891	39/5594	0.00145
**70153**	Glucose transport	24/1891	37/5594	0.00149
**176033**	Interactions of Vpr with host cellular proteins	22/1891	33/5594	0.00156
**5389840**	Mitochondrial translation elongation	39/1891	70/5594	0.00172
**5651801**	PCNA-Dependent Long Patch Base Excision Repair	13/1891	16/5594	0.00181
**77588**	SLBP Dependent Processing of Replication-Dependent Histone Pre-mRNAs	10/1891	11/5594	0.00195
**15869**	Metabolism of nucleotides	41/1891	75/5594	0.00197
**176814**	Activation of APC/C and APC/C:Cdc20 mediated degradation of mitotic proteins	37/1891	66/5594	0.00203
**110373**	Resolution of AP sites via the multiple-nucleotide patch replacement pathway	14/1891	18/5594	0.00215
**5607761**	Dectin-1 mediated noncanonical NF-kB signaling	31/1891	53/5594	0.00234
**69656**	Cyclin A:Cdk2-associated events at S phase entry	31/1891	53/5594	0.00234
**109581**	Apoptosis	67/1891	138/5594	0.00252
**176408**	Regulation of APC/C activators between G1/S and early anaphase	38/1891	69/5594	0.00258
**168325**	Viral Messenger RNA Synthesis	18/1891	26/5594	0.00276
**170822**	Regulation of Glucokinase by Glucokinase Regulatory Protein	19/1891	28/5594	0.00276
**180910**	Vpr-mediated nuclear import of PICs	20/1891	30/5594	0.00276
**5358565**	Mismatch repair (MMR) directed by MSH2:MSH6 (MutSalpha)	11/1891	13/5594	0.00278
**69601**	Ubiquitin Mediated Degradation of Phosphorylated Cdc25A	28/1891	47/5594	0.00278

**69610**	p53-Independent DNA Damage Response	28/1891	47/5594	0.00278
**69613**	p53-Independent G1/S DNA damage checkpoint	28/1891	47/5594	0.00278
**169911**	Regulation of Apoptosis	27/1891	45/5594	0.00291
**69229**	Ubiquitin-dependent degradation of Cyclin D1	27/1891	45/5594	0.00291
**75815**	Ubiquitin-dependent degradation of Cyclin D	27/1891	45/5594	0.00291
**176409**	APC/C:Cdc20 mediated degradation of mitotic proteins	36/1891	65/5594	0.00291
**179419**	APC:Cdc20 mediated degradation of cell cycle proteins prior to satisfation of the cell cycle checkpoint	35/1891	63/5594	0.00316
**74159**	Transcription	70/1891	147/5594	0.00324
**69473**	G2/M DNA damage checkpoint	12/1891	15/5594	0.00338
**69615**	G1/S DNA Damage Checkpoints	30/1891	52/5594	0.00340
**111367**	SLBP independent Processing of Histone Pre-mRNAs	9/1891	10/5594	0.00404
**180585**	Vif-mediated degradation of APOBEC3G	28/1891	48/5594	0.00408
**1538133**	G0 and Early G1	14/1891	19/5594	0.00432
**2151201**	Transcriptional activation of mitochondrial biogenesis	14/1891	19/5594	0.00432
**3301854**	Nuclear Pore Complex (NPC) Disassembly	20/1891	31/5594	0.00432
**187577**	SCF(Skp2)-mediated degradation of p27/p21	27/1891	46/5594	0.00432
**450531**	Regulation of mRNA stability by proteins that bind AU-rich elements	41/1891	78/5594	0.00437
**983189**	Kinesins	15/1891	21/5594	0.00446
**211733**	Regulation of activated PAK-2p34 by proteasome mediated degradation	26/1891	44/5594	0.00454
**350562**	Regulation of ornithine decarboxylase (ODC)	26/1891	44/5594	0.00454
**69017**	CDK-mediated phosphorylation and removal of Cdc6	26/1891	44/5594	0.00454
**174184**	Cdc20:Phospho-APC/C mediated degradation of Cyclin A	34/1891	62/5594	0.00458
**5368287**	Mitochondrial translation	40/1891	76/5594	0.00467
**1236978**	Cross-presentation of soluble exogenous antigens (endosomes)	24/1891	40/5594	0.00531
**111447**	Activation of BAD and translocation to mitochondria	10/1891	12/5594	0.00538
**75035**	Chk1/Chk2(Cds1) mediated inactivation of Cyclin B:Cdk1 complex	10/1891	12/5594	0.00538
**174113**	SCF-beta-TrCP mediated degradation of Emi1	28/1891	49/5594	0.00562
**5419276**	Mitochondrial translation termination	37/1891	70/5594	0.00644
**5358508**	Mismatch Repair	11/1891	14/5594	0.00670
**391251**	Protein folding	20/1891	32/5594	0.00688
**73847**	Purine metabolism	20/1891	32/5594	0.00688
**69231**	Cyclin D associated events in G1	18/1891	28/5594	0.00765
**69236**	G1 Phase	18/1891	28/5594	0.00765
**419524**	Fanconi Anemia pathway	16/1891	24/5594	0.00822
**114452**	Activation of BH3-only proteins	15/1891	22/5594	0.00829
**174178**	APC/C:Cdh1 mediated degradation of Cdc20 and other APC/C:Cdh1 targeted proteins in late mitosis/early G1	34/1891	64/5594	0.00867
**180534**	Vpu mediated degradation of CD4	26/1891	46/5594	0.00999
**425374**	Amino acid and oligopeptide SLC transporters	25/1891	44/5594	0.01107
**202733**	Cell surface interactions at the vascular wall	42/1891	84/5594	0.01162
**174414**	Processive synthesis on the C-strand of the telomere	9/1891	11/5594	0.01180
**73817**	Purine ribonucleoside monophosphate biosynthesis	9/1891	11/5594	0.01180
**73884**	Base Excision Repair	19/1891	31/5594	0.01180
**425407**	SLC-mediated transmembrane transport	99/1891	229/5594	0.01180

**352230**	Amino acid transport across the plasma membrane	18/1891	29/5594	0.01228
**450408**	AUF1 (hnRNP D0) destabilizes mRNA	27/1891	49/5594	0.01228
**69563**	p53-Dependent G1 DNA Damage Response	27/1891	49/5594	0.01228
**69580**	p53-Dependent G1/S DNA damage checkpoint	27/1891	49/5594	0.01228
**3108214**	SUMOylation of DNA damage response and repair proteins	36/1891	70/5594	0.01228
**3108232**	SUMO E3 ligases SUMOylate target proteins	36/1891	70/5594	0.01228
**5368286**	Mitochondrial translation initiation	36/1891	70/5594	0.01228
**1236974**	ER-Phagosome pathway	30/1891	56/5594	0.01256
**2990846**	SUMOylation	38/1891	75/5594	0.01279
**5358606**	Mismatch repair (MMR) directed by MSH2:MSH3 (MutSbeta)	10/1891	13/5594	0.01308
**110314**	Recognition of DNA damage by PCNA-containing replication complex	16/1891	25/5594	0.01325
**606279**	Deposition of new CENPA-containing nucleosomes at the centromere	22/1891	38/5594	0.01325
**774815**	Nucleosome assembly	22/1891	38/5594	0.01325
**69541**	Stabilization of p53	26/1891	47/5594	0.01325
**1169408**	ISG15 antiviral mechanism	32/1891	61/5594	0.01332
**1169410**	Antiviral mechanism by IFN-stimulated genes	32/1891	61/5594	0.01332
**73780**	RNA Polymerase III Chain Elongation	11/1891	15/5594	0.01365
**73980**	RNA Polymerase III Transcription Termination	11/1891	15/5594	0.01365
**3371453**	Regulation of HSF1-mediated heat shock response	28/1891	52/5594	0.01482
**109688**	Cleavage of Growing Transcript in the Termination Region	24/1891	43/5594	0.01568
**73856**	RNA Polymerase II Transcription Termination	24/1891	43/5594	0.01568
**76044**	Post-Elongation Processing of the Transcript	24/1891	43/5594	0.01568
**4641258**	degradation of DVL	27/1891	50/5594	0.01630
**109606**	Intrinsic Pathway for Apoptosis	19/1891	32/5594	0.01680
**2980766**	Nuclear Envelope Breakdown	23/1891	41/5594	0.01724
**1268020**	Mitochondrial protein import	18/1891	30/5594	0.01801
**73933**	Resolution of Abasic Sites (AP sites)	18/1891	30/5594	0.01801
**983168**	Antigen processing: Ubiquitination & Proteasome degradation	26/1891	48/5594	0.01801
**379724**	tRNA Aminoacylation	22/1891	39/5594	0.01884
**68875**	Mitotic Prophase	36/1891	72/5594	0.01941
**349425**	Autodegradation of the E3 ubiquitin ligase COP1	25/1891	46/5594	0.01970
**5610783**	Degradation of GLI2 by the proteasome	28/1891	53/5594	0.01970
**1236975**	Antigen processing-Cross presentation	33/1891	65/5594	0.02045
**70263**	Gluconeogenesis	16/1891	26/5594	0.02071
**113507**	E2F-enabled inhibition of pre-replication complex formation	8/1891	10/5594	0.02250
**983169**	Class I MHC mediated antigen processing & presentation	39/1891	80/5594	0.02280
**4641257**	degradation of AXIN	26/1891	49/5594	0.02451
**73937**	Transcription-coupled NER (TC-NER)	22/1891	40/5594	0.02713
**174154**	APC/C:Cdc20 mediated degradation of Securin	30/1891	59/5594	0.02884
**73857**	RNA Polymerase II Transcription	47/1891	101/5594	0.02942
**5610780**	Degradation of GLI1 by the proteasome	27/1891	52/5594	0.03002
**1592230**	Mitochondrial biogenesis	16/1891	27/5594	0.03340
**5362768**	Hh mutants that don't undergo autocatalytic processing are degraded by ERAD	25/1891	48/5594	0.03802
**5387390**	Hh mutants abrogate ligand secretion	25/1891	48/5594	0.03802

**112297**	Post-Elongation Processing of Intronless pre-mRNA	14/1891	23/5594	0.03964
**75067**	Processing of Capped Intronless Pre-mRNA	14/1891	23/5594	0.03964
**5610785**	GLI3 is processed to GLI3R by the proteasome	27/1891	53/5594	0.04038
**109970**	Global Genomic NER (GG-NER)	18/1891	32/5594	0.04118
**73890**	Double-Strand Break Repair	13/1891	21/5594	0.04259
**162587**	HIV Life Cycle	56/1891	126/5594	0.04295

Table S12: Significantly enriched KEGG pathways in MKN1 cells after 4 h afatinib treatment.

ID	Description	GeneRatio	BgRatio	p.adjust
**hsa04668**	TNF signaling pathway	10/66	102/5844	2.3777E-05
**hsa05323**	Rheumatoid arthritis	8/66	70/5844	7.028E-05
**hsa04657**	IL-17 signaling pathway	8/66	79/5844	0.00011952
**hsa04060**	Cytokine-cytokine receptor interaction	11/66	186/5844	0.00022227
**hsa04933**	AGE-RAGE signaling pathway in diabetic complications	8/66	95/5844	0.00028978
**hsa04010**	MAPK signaling pathway	11/66	272/5844	0.00427566
**hsa05132**	Salmonella infection	6/66	75/5844	0.00427566
**hsa05142**	Chagas disease (American trypanosomiasis)	6/66	81/5844	0.00519986
**hsa05144**	Malaria	4/66	35/5844	0.01002006
**hsa05206**	MicroRNAs in cancer	7/66	149/5844	0.02029889
**hsa05167**	Kaposi's sarcoma-associated herpesvirus infection	7/66	155/5844	0.02319695
**hsa05200**	Pathways in cancer	13/66	469/5844	0.02329389
**hsa04064**	NF-kappa B signaling pathway	5/66	82/5844	0.02329389
**hsa05146**	Amoebiasis	5/66	82/5844	0.02329389
**hsa05134**	Legionellosis	4/66	50/5844	0.02329389
**hsa05202**	Transcriptional misregulation in cancer	7/66	168/5844	0.024013
**hsa05161**	Hepatitis B	6/66	125/5844	0.024013
**hsa05166**	HTLV-I infection	8/66	231/5844	0.03368798
**hsa05140**	Leishmaniasis	4/66	59/5844	0.03368798
**hsa04621**	NOD-like receptor signaling pathway	6/66	140/5844	0.0343651
**hsa04610**	Complement and coagulation cascades	4/66	61/5844	0.0343651
**hsa04062**	Chemokine signaling pathway	6/66	145/5844	0.03858289
**hsa05133**	Pertussis	4/66	65/5844	0.03936097
**hsa04640**	Hematopoietic cell lineage	4/66	69/5844	0.04657383

Table S13: Significantly enriched KEGG pathways in MKN1 cells after 24 h afatinib treatment.

ID	Description	GeneRatio	BgRatio	p.adjust
**hsa04640**	Hematopoietic cell lineage	11/206	69/5844	0.00617019

Table S14: Significantly enriched Reactome pathways in MKN1 cells after 4 h afatinib treatment.

ID	Description	GeneRatio	BgRatio	p.adjust
**380108**	Chemokine receptors bind chemokines	5/60	31/5594	0.00376562
**937061**	TRIF-mediated TLR3/TLR4 signaling	6/60	76/5594	0.00709969
**450282**	MAPK targets/ Nuclear events mediated by MAP kinases	4/60	26/5594	0.00709969
**166166**	MyD88-independent TLR3/TLR4 cascade	6/60	77/5594	0.00709969
**168164**	Toll Like Receptor 3 (TLR3) Cascade	6/60	77/5594	0.00709969
**166054**	Activated TLR4 signalling	6/60	89/5594	0.01312667
**166016**	Toll Like Receptor 4 (TLR4) Cascade	6/60	92/5594	0.01346718
**198753**	ERK/MAPK targets	3/60	17/5594	0.0159905
**168898**	Toll-Like Receptors Cascades	6/60	105/5594	0.0159905
**166058**	MyD88:Mal cascade initiated on plasma membrane	5/60	71/5594	0.0159905
**168179**	Toll Like Receptor TLR1:TLR2 Cascade	5/60	71/5594	0.0159905
**168188**	Toll Like Receptor TLR6:TLR2 Cascade	5/60	71/5594	0.0159905
**181438**	Toll Like Receptor 2 (TLR2) Cascade	5/60	71/5594	0.0159905
**198725**	Nuclear Events (kinase and transcription factor activation)	3/60	20/5594	0.01917469
**373076**	Class A/1 (Rhodopsin-like receptors)	8/60	202/5594	0.01917469
**500792**	GPCR ligand binding	9/60	253/5594	0.01917469
**375276**	Peptide ligand-binding receptors	6/60	119/5594	0.02157578
**450294**	MAP kinase activation in TLR cascade	4/60	49/5594	0.02248863
**168180**	TRAF6 Mediated Induction of proinflammatory cytokines	4/60	56/5594	0.03492941
**3000178**	ECM proteoglycans	4/60	59/5594	0.04014859
**975138**	TRAF6 mediated induction of NFkB and MAP kinases upon TLR7/8 or 9 activation	4/60	64/5594	0.04380237
**168142**	Toll Like Receptor 10 (TLR10) Cascade	4/60	65/5594	0.04380237
**168176**	Toll Like Receptor 5 (TLR5) Cascade	4/60	65/5594	0.04380237
**168181**	Toll Like Receptor 7/8 (TLR7/8) Cascade	4/60	65/5594	0.04380237
**975155**	MyD88 dependent cascade initiated on endosome	4/60	65/5594	0.04380237
**975871**	MyD88 cascade initiated on plasma membrane	4/60	65/5594	0.04380237
**168138**	Toll Like Receptor 9 (TLR9) Cascade	4/60	67/5594	0.04699983
**381042**	PERK regulates gene expression	2/60	11/5594	0.04751258
**1989781**	PPARA activates gene expression	3/60	35/5594	0.04751258

Table S15: Significantly enriched Reactome pathways in MKN1 cells after 24 h afatinib treatment.

ID	Description	GeneRatio	BgRatio	p.adjust
**1989781**	PPARA activates gene expression	7/180	35/5594	0.04242702

Table S16: Significantly enriched KEGG pathways in MKN7 cells after 4 h afatinib treatment.

ID	Description	GeneRatio	BgRatio	p.adjust
**hsa01522**	Endocrine resistance	9/157	90/5844	0.0337614
**hsa04668**	TNF signaling pathway	10/157	102/5844	0.02646242
**hsa04060**	Cytokine-cytokine receptor interaction	16/157	186/5844	0.00355486
**hsa04630**	Jak-STAT signaling pathway	14/157	125/5844	0.00112593

Table S17: Significantly enriched KEGG pathways in MKN7 cells after 24 h afatinib treatment.

ID	Description	GeneRatio	BgRatio	p.adjust
**hsa03008**	Ribosome biogenesis in eukaryotes	25/785	69/5844	0.00039694
**hsa04110**	Cell cycle	33/785	118/5844	0.0030143
**hsa03030**	DNA replication	13/785	33/5844	0.01815882

Table S18: Significantly enriched Reactome pathways in MKN7 cells after 24 h afatinib treatment.

ID	Description	GeneRatio	BgRatio	p.adjust
**453279**	Mitotic G1-G1/S phases	40/764	114/5594	3.6681E-06
**69278**	Cell Cycle, Mitotic	91/764	380/5594	6.4007E-06
**69206**	G1/S Transition	34/764	94/5594	7.6198E-06
**113510**	E2F mediated regulation of DNA replication	16/764	28/5594	1.4565E-05
**1640170**	Cell Cycle	103/764	462/5594	1.4565E-05
**69306**	DNA Replication	32/764	92/5594	2.8265E-05
**69205**	G1/S-Specific Transcription	10/764	13/5594	5.269E-05
**69190**	DNA strand elongation	16/764	31/5594	5.6795E-05
**69242**	S Phase	35/764	110/5594	6.0213E-05
**176187**	Activation of ATR in response to replication stress	17/764	35/5594	6.1092E-05
**69481**	G2/M Checkpoints	20/764	49/5594	0.00018433
**69239**	Synthesis of DNA	29/764	88/5594	0.000189
**68962**	Activation of the pre-replicative complex	14/764	28/5594	0.00029987
**68877**	Mitotic Prometaphase	29/764	91/5594	0.00034588
**2500257**	Resolution of Sister Chromatid Cohesion	27/764	85/5594	0.00072655
**69620**	Cell Cycle Checkpoints	33/764	116/5594	0.00105427
**180786**	Extension of Telomeres	12/764	24/5594	0.00113179
**68874**	M/G1 Transition	23/764	72/5594	0.0022782
**69002**	DNA Replication Pre-Initiation	23/764	72/5594	0.0022782
**73886**	Chromosome Maintenance	22/764	69/5594	0.00325081
**2555396**	Mitotic Metaphase and Anaphase	37/764	149/5594	0.0064384
**176974**	Unwinding of DNA	7/764	11/5594	0.00668213
**73817**	Purine ribonucleoside monophosphate biosynthesis	7/764	11/5594	0.00668213
**2467813**	Separation of Sister Chromatids	35/764	141/5594	0.00851589
**174417**	Telomere C-strand (Lagging Strand) Synthesis	10/764	22/5594	0.00996357
**68882**	Mitotic Anaphase	36/764	148/5594	0.00996357
**69186**	Lagging Strand Synthesis	9/764	20/5594	0.02130541

**15869**	Metabolism of nucleotides	21/764	75/5594	0.02459538
**113507**	E2F-enabled inhibition of pre-replication complex formation	6/764	10/5594	0.02513251
**68886**	M Phase	46/764	216/5594	0.03263143
**69300**	Removal of licensing factors from origins	18/764	64/5594	0.04742088
**69304**	Regulation of DNA replication	18/764	64/5594	0.04742088

Table S19: Regulated genes after afatinib and trastuzumab + afatinib treatment related to the biological function "positive regulation of cell motility". In MKN1 cells regulated genes are colored in blue.

MKN1 4h Afa vs. MKN1 4h untr	MKN1 4h Tra+Afa vs. MKN1 4h untr	MKN7 4h Afa vs. MKN7 4h untr	MKN7 4h Tra+Afa vs. MKN7 4h untr	NCI-N87 4h Afa vs. NCI-N87 4h untr	NCI-N87 4h Tra+Afa vs. NCI-N87 4h untr
CCR7	*CXCL1*	*ACKR3*	*SEMA7A*	*ARHGEF2*	*ARHGEF2*
CXCL1	*CXCL8*	*ADAM8*	*ACKR3*	*ATOH8*	*ATOH8*
CXCL8	*CYR61*	*ARHGEF2*	*ADAM8*	*CXCL1*	*BCL2*
CYR61	*ETS1*	*BCL2*	*ARHGEF2*	*CXCL2*	*CXCL1*
ETS1	*F2RL1*	*CCL20*	*BCL2*	*CXCL3*	*CXCL2*
F2RL1	*F3*	*CD274*	*CCL20*	*CXCL8*	*CXCL3*
F3	*HAS2*	*DRD1*	*CD274*	*EDN2*	*CXCL8*
HAS2	*HBEGF*	*ETS1*	*CXCL12*	*F3*	*EDN2*
HBEGF	*ITGA2*	*FLT1*	*DRD1*	*GRB7*	*ENPP2*
IL1B	*PLPP3*	*HBEGF*	*ETS1*	*HAS2*	*F3*
ITGA2	*PTGS2*	*ITGA2*	*HBEGF*	*HBEGF*	*GRB7*
PDGFB	*SEMA6D*	*NRP1*	*ITGA2*	*HMCN2*	*HAS2*
PLPP3	*SERPINE1*	*PTAFR*	*LEF1*	*NOS3*	*HBEGF*
PTGS2	*SPRY2*	*PTGS2*	*MADCAM1*	*RHOB*	*ITGA2*
SEMA6D	*TNFSF14*	*PTK2B*	*NRP1*	*SNAI1*	*JCAD*
SERPINE1		*SEMA4B*	*PTGS2*	*SPRY2*	*NOS3*
SPRY2		*SEMA7A*	*PTK2B*	*ZC3H12A*	*RHOB*
VEGFC		*SERPINE1*	*SEMA4B*		*SNAI1*
		SMURF2	*SERPINE1*		*SPRY2*
		SPRY2	*SMURF2*		
		VEGFD	*SPRY2*		
			TERT		

Table S20: Regulated genes after afatinib and trastuzumab + afatinib treatment related to the biological function "positive regulation of apoptotic signaling pathway". In NCI-N87 cells exclusively regulated genes are colored in blue.

MKN1 24h Cet vs. MKN1 24h untr	MKN1 24h Afa vs. MKN1 24h untr	MKN1 24h Tra+Afa vs. MKN1 24h untr	MKN7 24h Afa vs. MKN7 24h untr	MKN7 24h Tra+Afa vs. MKN7 24h untr	NCI-N87 24h Afa vs. NCI-N87 24h untr	NCI-N87 24h Tra+Afa vs. NCI-N87 24h untr
BMF	*AGT*	*ATF3*	*BCL2*	*BCL2*	*BAX*	*BAX*
TNFSF10	*ATF3*	*BCL2*	*BCL2L1*	*BCL2L1*	*BBC3*	*BBC3*
	BCL2	*BMF*	*BID*	*BID*	*BCL2*	*BCL2*
	BMF	*TNFRSF12A*	*CTSC*	*CTSC*	*BCL2L1*	*BCL2L1*
	TNFSF10	*TNFSF10*	*FAM162A*	*FAM162A*	*BCLAF1*	*BCLAF1*
			HRK	*HRK*	*BID*	*BID*
			MMP9	*MMP9*	*BMF*	*BMF*
			PLA2G6	*NOX1*	*CAV1*	*CAV1*
			PLAUR	*PLA2G6*	*CTSC*	*CTSC*
			S100A9	*PLAUR*	*E2F1*	*DYNLL1*
			SFRP1	*PMAIP1*	*FADD*	*DYNLL2*
			TFAP4	*S100A9*	*FAF1*	*E2F1*
			TFDP1	*SFRP1*	*FAS*	*FADD*
			TNFRSF10A	*TFAP4*	*GSN*	*FAF1*
			TNFRSF10B	*TFDP1*	*HYAL2*	*FAM162A*
				TNFRSF10A	*IL19*	*FAS*
				TNFRSF10B	*IL20RA*	*GSK3A*
					INHBB	*GSN*
					LCK	*HYAL2*
					LGALS9	*IFNB1*
					MMP9	*IL19*
					NACC2	*IL20RA*
					NF1	*INHBB*
					NFATC4	*LCK*
					NKX3-1	*LGALS9*
					PAK2	*LTBR*
					PARK7	*MAL*
					PDCD5	*NACC2*
					PDIA3	*NF1*
					PEA15	*NFATC4*
					PINK1	*NKX3-1*
					PLA2G6	*NMT1*
					PLAUR	*NOX1*
					PMAIP1	*PAK2*
					PPIF	*PARK7*
					PPP2R1B	*PDCD5*
					PPP3CC	*PDIA3*
					PRKRA	*PEA15*
					RIPK3	*PLA2G6*

	SFN	*PLAUR*
	SFPQ	*PMAIP1*
	SKIL	*PPIF*
	SLC9A3R1	*PPP1CA*
	SMAD3	*PPP2R1B*
	STK4	*PPP3CC*
	TFAP4	*PRKRA*
	TFDP1	*S100A9*
	TGFBR1	*SFN*
	TNFRSF10A	*SFPQ*
	TNFRSF10B	*SIRT1*
	TNFRSF12A	*SKIL*
	TNFSF10	*SLC9A3R1*
	TP73	*SMAD3*
	TPD52L1	*STK4*
	WDR35	*TFAP4*
	YWHAB	*TFDP1*
	YWHAE	*TGFBR1*
	YWHAG	*TNFRSF10A*
	YWHAH	*TNFRSF10B*
	YWHAQ	*TNFRSF12A*
	YWHAZ	*TNFSF10*
	ZNF205	*TP63*
		TP73
		TPD52L1
		WDR35
		YWHAB
		YWHAE
		YWHAG
		YWHAH
		YWHAQ
		YWHAZ
		ZNF205
