# Supplementary material for: Determining the effects of trastuzumab, cetuximab and afatinib by phosphoprotein, gene expression and phenotypic analysis in gastric cancer cell lines
Source: BMC Cancer. 2020 Oct 28;20:1039. doi: 10.1186/s12885-020-07540-7 (PMC7594334; doi:10.1186/s12885-020-07540-7)
Supplement: Supplementary file 5 — Additional file 5. Supplemental full-length blots corresponding to Fig. 8. [file 12885_2020_7540_MOESM5_ESM.pdf]

Experiment 1

Supplemental full-length blots corresponding to Figure 8

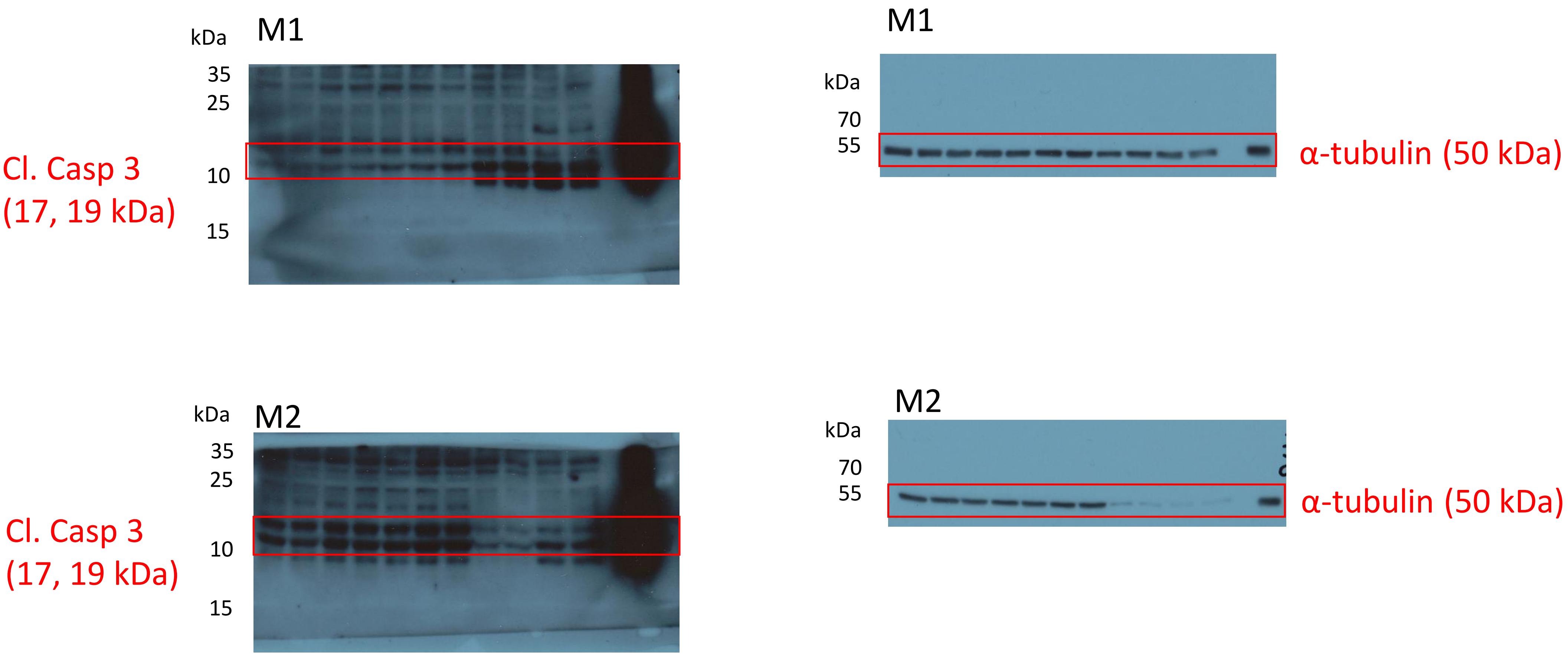

Membranes were cut before staining

Experiment 2

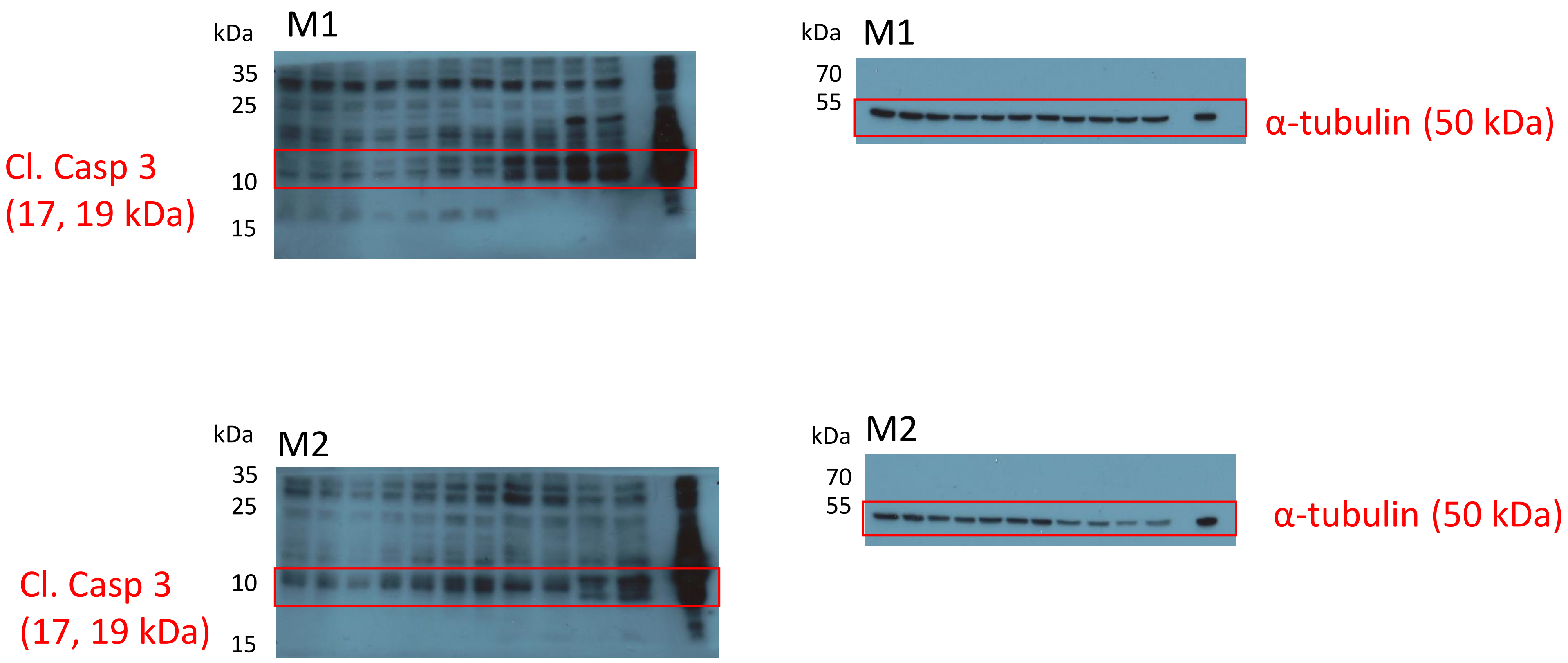

Membranes were cut before staining

Experiment 3

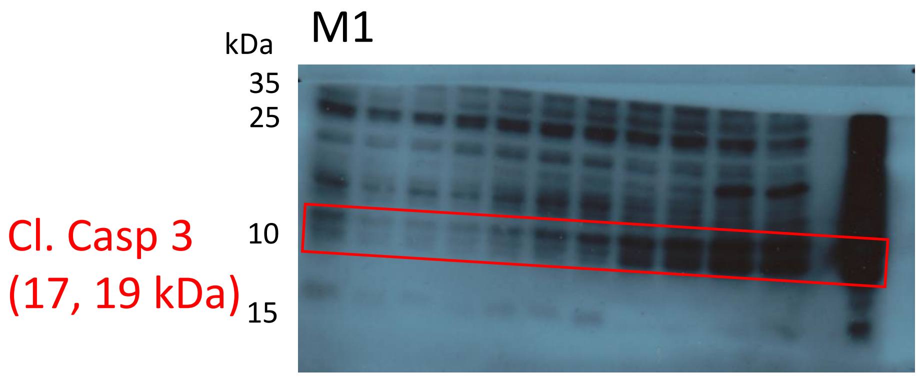

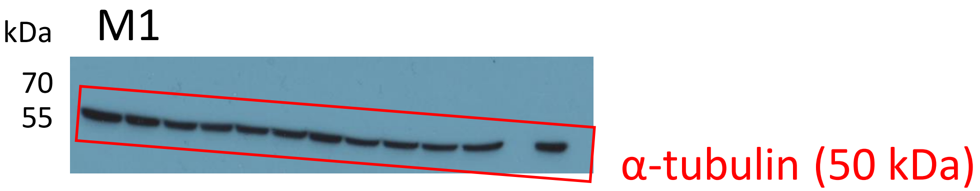

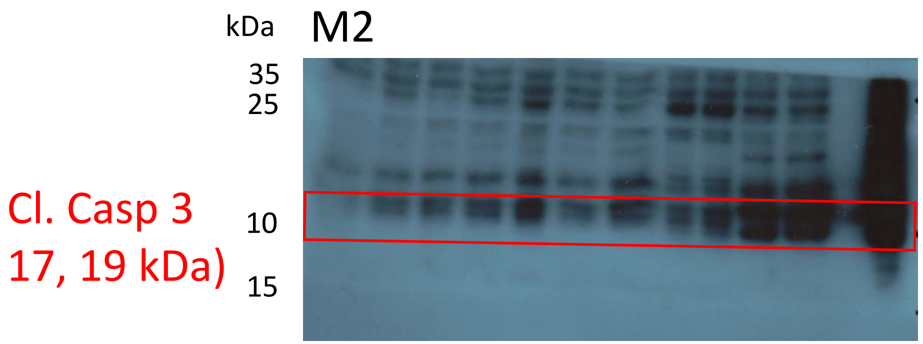

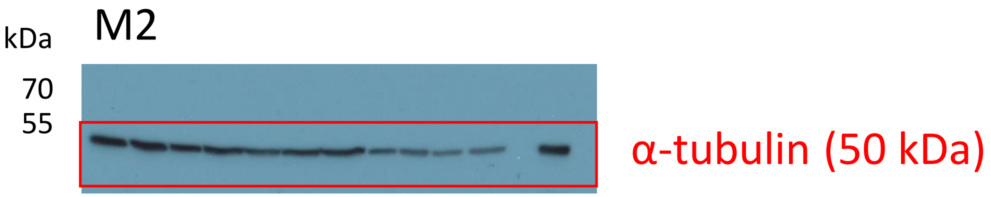

Membranes were cut before staining

M1

1	2	3	4	5	6	7	8	9	10	11	12	13	14	15
L	MKN1 untr	MKN1 EGF	MKN1 Cet	MKN1 EGF+Cet	MKN1 Tra	MKN1 Afa	MKN1 Tra+Afa	MKN7 untr	MKN7 Tra	MKN7 Afa	MKN7 TraAfa		Jurkat Cytochro me c	L

M2

1	2	3	4	5	6	7	8	9	10	11	12	13	14	15
L	Hs746T untr	Hs746T EGF	Hs746T Cet	Hs746T EGF+Cet	Hs746T Tra	Hs746T Afa	Hs746T Tra+Afa	N87 untr	N87 Tra	N87 Afa	N87 Tra+Afa		Jurkat Cytochro me c	L

Jurkat cell extracts in treated with Cytochrome c in vitro were used as positive control (Cell Signaling #9663S)
