# Supplementary material for: Determining the effects of trastuzumab, cetuximab and afatinib by phosphoprotein, gene expression and phenotypic analysis in gastric cancer cell lines
Source: BMC Cancer. 2020 Oct 28;20:1039. doi: 10.1186/s12885-020-07540-7 (PMC7594334; doi:10.1186/s12885-020-07540-7)

Experiment 1 Supplemental full-length blots corresponding to Additional File 2, Figure S3

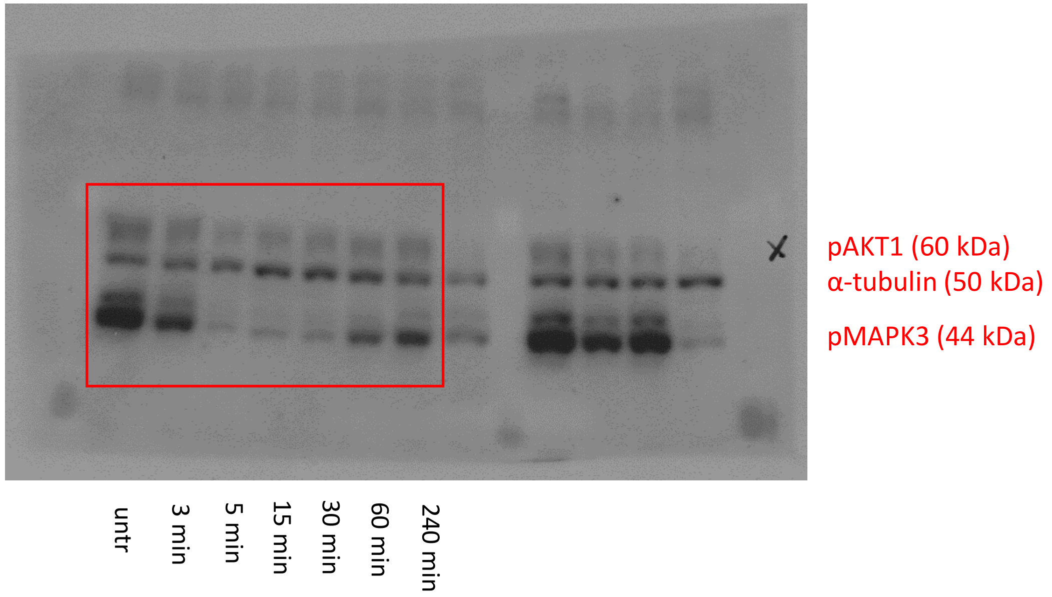

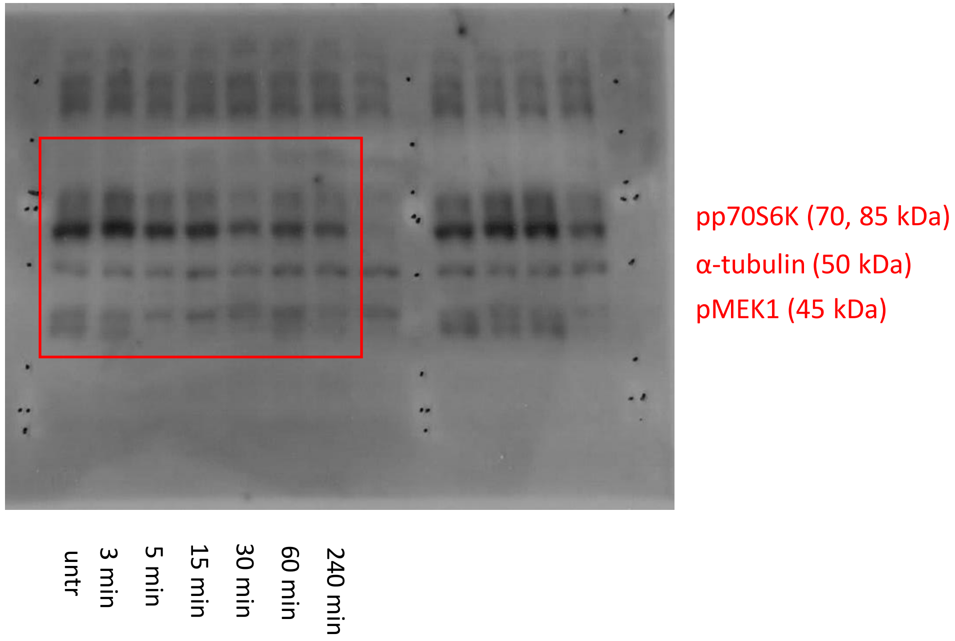

Experiment 2

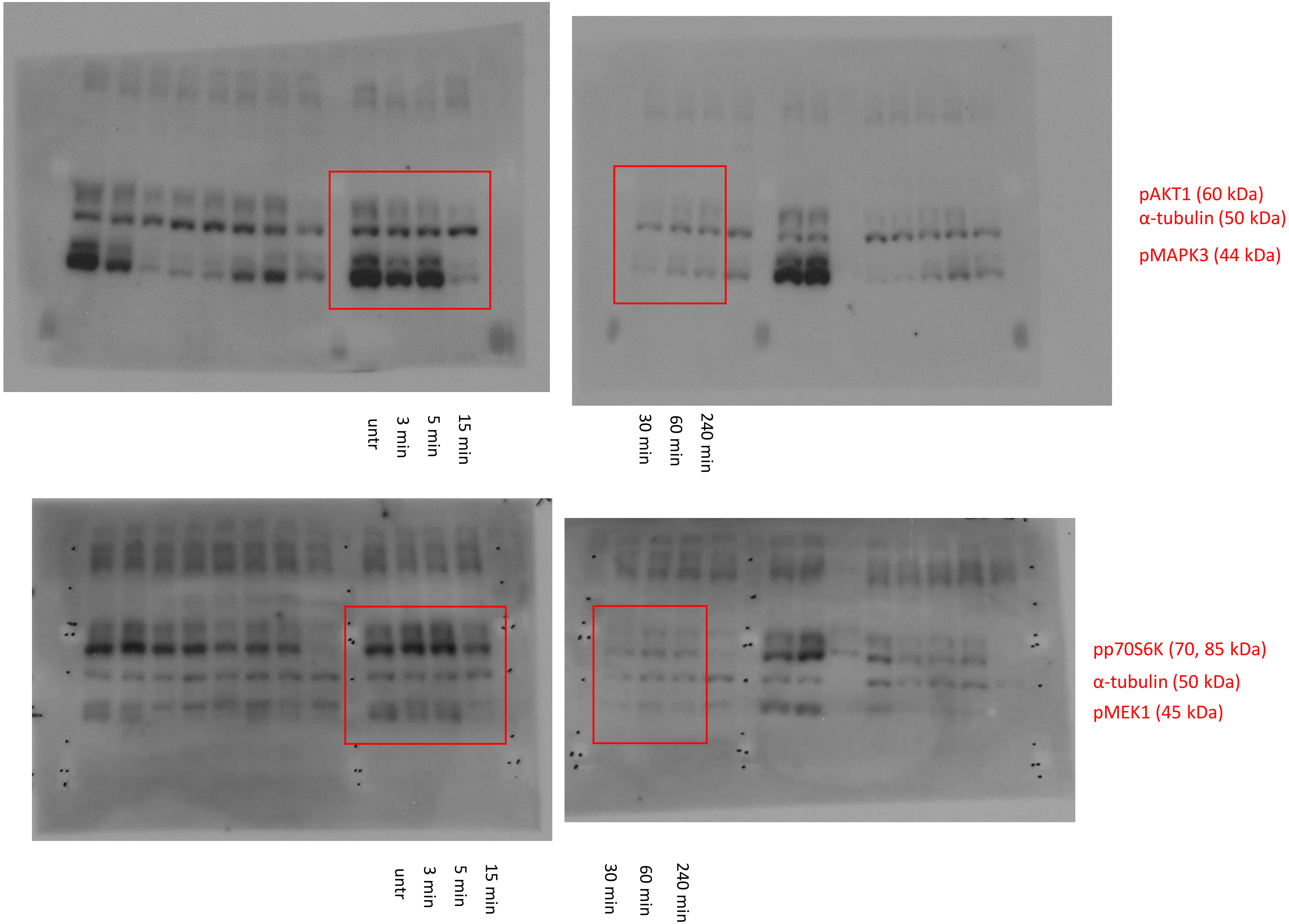

Experiment 3

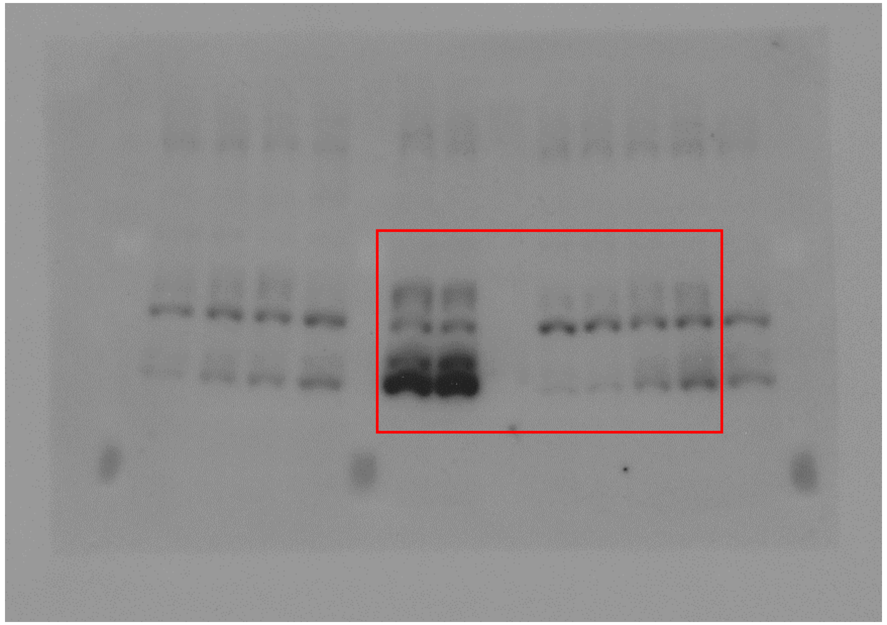

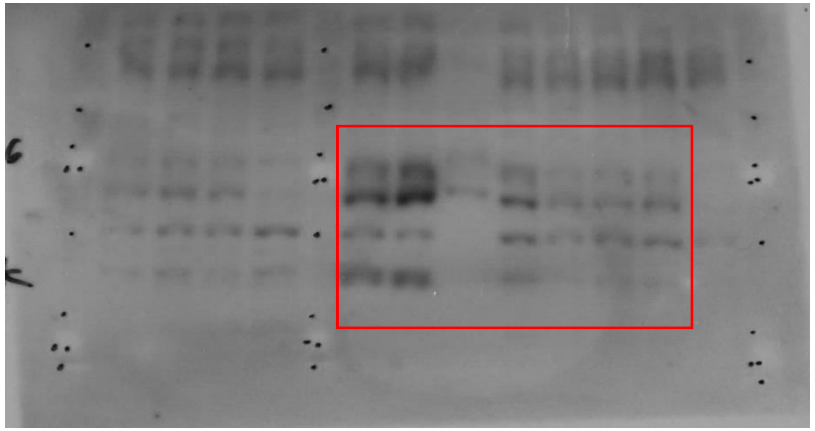

Supplement: Supplementary file 6 — Additional file 6. Supplemental full-length blots corresponding to Additional file 2, Fig. S3. [file 12885_2020_7540_MOESM6_ESM.pdf]
